# Supplementary material for: Antioxidant Activities of Alkyl Substituted Pyrazine Derivatives of Chalcones—In Vitro and In Silico Study
Source: Antioxidants (Basel). 2019 Apr 5;8(4):90. doi: 10.3390/antiox8040090 (PMC6523444; doi:10.3390/antiox8040090)
Supplement: Supplementary file 1 [file antioxidants-08-00090-s001.pdf]

Supplementary Materials

Antioxidant Activities of Alkyl Substituted Pyrazine Derivatives of Chalcones—In Vitro and In Silico Study

Višnja Stepanić [1,*], Mario Matijašić [2], Tea Horvat [1], Donatella Verbanac [2,3], Marta Kučerová-Chlupáčová [4], Luciano Saso [5] and Neven Žarković [1,*]

[1] Division of Molecular Medicine, Ruđer Bošković Institute, Bijenička cesta 54, HR-10000 Zagreb, Croatia; Tea.Horvat@irb.hr (T.H.)
[2] Department for Intercellular Communication, Centre for Translational and Clinical Research and Croatian Centre of Excellence for Reproductive and Regenerative Medicine, School of Medicine, University of Zagreb, Šalata 2, HR-10000 Zagreb, Croatia; mario.matijasic@mef.hr (M.M.)
[3] Department of Medical Biochemistry and Haematology, Faculty of Pharmacy and Biochemistry, University of Zagreb, Domagojeva 2, HR-10000 Zagreb, Croatia; dverbanac@pharma.hr (D.V.)
[4] Department of Pharmaceutical Chemistry and Pharmaceutical Analysis, Faculty of Pharmacy, Charles University, Heyrovského 1203, 500 05 Hradec Králové, Czech Republic; kucerom@faf.cuni.cz
[5] Department of Physiology and Pharmacology Vittorio Erspamer, Sapienza University, P.le Aldo Moro 5, 00185 Rome, Italy; luciano.saso@uniroma1.it
* Correspondence: stepanic@irb.hr (V.S.); zarkovic@irb.hr (N.Ž.); Tel.: +385-1-457-1248 (V.S.); +385-1-457-1234 (N.Ž.)

Table S1. SMILES, simple structural and physicochemical properties as well as toxicity risk alerts of studied compounds calculated by SwissADME and DataWarrior (DW).

Cmpd	Canonical SMILES	MW	#Heavy atoms	#Aromatic heavy atoms	Fraction Csp³	#Rotatable bonds	#H-bond acceptors	#H-bond donors	TPSA
1	O=C(c1ccccc1)/C=C/c1ccccc1O	224.25	17	12	0	3	2	1	37.3
1a	O=C(c1cnccn1)/C=C/c1ccccc1O	226.23	17	12	0	3	4	1	63.08
1b	CCCc1cnc(cn1)C(=O)/C=C/c1ccccc1O	268.31	20	12	0.19	5	4	1	63.08
1c	O=C(c1cnc(cn1)C(C)C)/C=C/c1ccccc1O	268.31	20	12	0.19	4	4	1	63.08
1d	CCCCc1cnc(cn1)C(=O)/C=C/c1ccccc1O	282.34	21	12	0.24	6	4	1	63.08
1e	CC(Cc1cnc(cn1)C(=O)/C=C/c1ccccc1O)C	282.34	21	12	0.24	5	4	1	63.08

1f	O=C(c1cnc(cn1)C(C)(C)C)/C=C/c1ccccc1O	282.34	21	12	0.24	4	4	1	63.08
2	Oc1cccc(c1)/C=C/C(=O)c1ccccc1	224.25	17	12	0	3	2	1	37.3
2a	Oc1cccc(c1)/C=C/C(=O)c1cnccn1	226.23	17	12	0	3	4	1	63.08
2b	CCCc1cnc(cn1)C(=O)/C=C/c1cccc(c1)O	268.31	20	12	0.19	5	4	1	63.08
2c	Oc1cccc(c1)/C=C/C(=O)c1cnc(cn1)C(C)C	268.31	20	12	0.19	4	4	1	63.08
2d	CCCCc1cnc(cn1)C(=O)/C=C/c1cccc(c1)O	282.34	21	12	0.24	6	4	1	63.08
2e	CC(Cc1cnc(cn1)C(=O)/C=C/c1cccc(c1)O)C	282.34	21	12	0.24	5	4	1	63.08
2f	Oc1cccc(c1)/C=C/C(=O)c1cnc(cn1)C(C)(C)C	282.34	21	12	0.24	4	4	1	63.08
3	Oc1ccc(cc1)/C=C/C(=O)c1ccccc1	224.25	17	12	0	3	2	1	37.3
3a	Oc1ccc(cc1)/C=C/C(=O)c1cnccn1	226.23	17	12	0	3	4	1	63.08
3b	CCCc1ncc(nc1)C(=O)/C=C/c1ccc(cc1)O	268.31	20	12	0.19	5	4	1	63.08
3c	Oc1ccc(cc1)/C=C/C(=O)c1cnc(cn1)C(C)C	268.31	20	12	0.19	4	4	1	63.08
3d	CCCCc1cnc(cn1)C(=O)/C=C/c1ccc(cc1)O	282.34	21	12	0.24	6	4	1	63.08
3e	CC(Cc1ncc(nc1)C(=O)/C=C/c1ccc(cc1)O)C	282.34	21	12	0.24	5	4	1	63.08
3f	Oc1ccc(cc1)/C=C/C(=O)c1cnc(cn1)C(C)(C)C	282.34	21	12	0.24	4	4	1	63.08
4	COc1cc(/C=C/C(=O)c2ccccc2)ccc1O	254.28	19	12	0.06	4	3	1	46.53
4a	COc1cc(/C=C/C(=O)c2cnccn2)ccc1O	256.26	19	12	0.07	4	5	1	72.31

4c	COc1cc(/C=C/C(=O)c2cnc(cn 2)C(C)C)ccc1O	298.34	22	12	0.24	5	5	1	72.31
4e	COc1cc(/C=C/C(=O)c2cnc(cn 2)CC(C)C)ccc1O	312.36	23	12	0.28	6	5	1	72.31
4f	COc1cc(/C=C/C(=O)c2cnc(cn 2)C(C)(C)C)ccc1O	312.36	23	12	0.28	5	5	1	72.31

TPSA-total polar surface area

Cont.

Cmpd	XLOGP 3	WLOG P	Solubility_class	Lipinski #violations	GI absorption	Bioavailability Score	BBB permeant	Pgp substrate
1	3.85	3.18	Moderately soluble	0	High	0.55	Yes	No
1a	1.53	1.97	Soluble	0	High	0.55	Yes	No
1b	2.72	2.92	Soluble	0	High	0.55	Yes	No
1c	2.69	3.09	Soluble	0	High	0.55	Yes	No
1d	3.26	3.31	Moderately soluble	0	High	0.55	Yes	No
1e	3.16	3.17	Moderately soluble	0	High	0.55	Yes	No
1f	3.24	3.27	Moderately soluble	0	High	0.55	Yes	No
2	2.72	3.18	Soluble	0	High	0.55	Yes	No
2a	1.53	1.97	Soluble	0	High	0.55	Yes	No
2b	2.72	2.92	Soluble	0	High	0.55	Yes	No
2c	2.69	3.09	Soluble	0	High	0.55	Yes	No
2d	3.26	3.31	Moderately soluble	0	High	0.55	Yes	No
2e	3.16	3.17	Moderately soluble	0	High	0.55	Yes	No
2f	3.24	3.27	Moderately soluble	0	High	0.55	Yes	No
3	2.72	3.18	Soluble	0	High	0.55	Yes	No
3a	1.53	1.97	Soluble	0	High	0.55	Yes	No

3b	2.72	2.92	Soluble	0	High	0.55	Yes	No
3c	2.69	3.09	Soluble	0	High	0.55	Yes	No
3d	3.26	3.31	Moderately soluble	0	High	0.55	Yes	No
3e	3.16	3.17	Moderately soluble	0	High	0.55	Yes	No
3f	3.24	3.27	Moderately soluble	0	High	0.55	Yes	No
4	3.39	3.19	Moderately soluble	0	High	0.55	Yes	No
4a	1.5	1.98	Soluble	0	High	0.55	Yes	No
4c	2.66	3.1	Soluble	0	High	0.55	Yes	No
4e	3.13	3.18	Moderately soluble	0	High	0.55	Yes	No
4f	3.21	3.28	Moderately soluble	0	High	0.55	Yes	No

Bioavailability Score – Probability of F > 10% in rat, implemented from Martin Y. C. A Bioavailability Score. J. Med. Chem. 48, 3164–3170 (2005). This semi-quantitative rule-based score relying on total charge, TPSA, and violation to the Lipinski filter defines four classes of compounds with probabilities of 11%, 17%, 56% or 85%.

Cont.

Cmpd	cLogP_DW	cLogS_DW	Druglikeness_DW	Shape Index_DW	Molecular Flexibility_DW	Mutagenic_DW	Tumorigenic_DW	Reproductive Effective_DW	Irritant_DW
1	2.9581	-3.544	0.1336	0.6471	0.3815	none	none	none	none
1a	1.0103	-1.978	0.3094	0.6471	0.2701	none	none	none	none
1b	2.2782	-2.775	-1.98	0.7	0.344	none	none	none	none
1c	2.2513	-2.873	0.0732	0.65	0.2968	none	none	none	none
1d	2.7326	-3.045	-5.25	0.7143	0.3653	none	none	none	none
1e	2.4965	-2.935	0.1589	0.6667	0.3703	none	none	none	none
1f	2.6462	-3.162	-2.846	0.6191	0.3303	none	none	none	none
2	2.9581	-3.544	0.1336	0.6471	0.4231	none	none	none	none
2a	1.0103	-1.978	0.3094	0.6471	0.3204	none	none	none	none
2b	2.2782	-2.775	-1.98	0.7	0.3807	none	none	none	none
2c	2.2513	-2.873	0.0732	0.65	0.3367	none	none	none	none
2d	2.7326	-3.045	-5.25	0.7143	0.3983	none	none	none	none
2e	2.4965	-2.935	0.1589	0.6667	0.403	none	none	none	none
2f	2.6462	-3.162	-2.846	0.6191	0.3657	none	none	none	none
3	2.9581	-3.544	0.1336	0.7059	0.4231	none	none	none	none
3a	1.0103	-1.978	0.3094	0.7059	0.3204	none	none	none	none
3b	2.2782	-2.775	-1.98	0.75	0.3807	none	none	none	none
3c	2.2513	-2.873	0.0732	0.7	0.3367	none	none	none	none
3d	2.7326	-3.045	-5.25	0.7619	0.3983	none	none	none	none
3e	2.4965	-2.935	0.1589	0.7143	0.403	none	none	none	none
3f	2.6462	-3.162	-2.846	0.6667	0.3657	none	none	none	none
4	2.8881	-3.562	0.219	0.6316	0.4056	none	none	none	none
4a	0.9403	-1.996	0.3928	0.6316	0.3144	none	none	none	none
4c	2.1813	-2.891	0.216	0.6364	0.3328	none	none	none	none
4e	2.4265	-2.953	0.3105	0.6522	0.3932	none	none	none	none
4f	2.5762	-3.18	-2.7	0.6087	0.3593	none	none	none	none

Drug-likeness (http://www.openmolecules.org/propertyexplorer/druglikeness.html): for most od drugs have this parameter is within range [-1,3]. Molecular flexibility (low < 0.5 < high) and shape index (spherical < 0.5 <linear).

DFT EQILIBRIUM STRUCTURES in sdf FORMAT

4

```
 33 34  0  0  0  0  0  0  0  0  0 V2000
   -3.5845   -0.2684    0.0115 C   0  0  0  0  0  0  0  0  0  0  0  0
   -2.2379   -0.9214   -0.0446 C   0  0  0  0  0  0  0  0  0  0  0  0
   -2.1524   -2.1341   -0.0774 O   0  0  0  0  0  0  0  0  0  0  0  0
   -1.0394   -0.0528   -0.0840 C   0  0  0  0  0  0  0  0  0  0  0  0
    0.1758   -0.6102   -0.0042 C   0  0  0  0  0  0  0  0  0  0  0  0
    1.4701    0.0618   -0.0528 C   0  0  0  0  0  0  0  0  0  0  0  0
   -4.6881   -1.0184   -0.3958 C   0  0  0  0  0  0  0  0  0  0  0  0
   -5.9593   -0.4678   -0.3590 C   0  0  0  0  0  0  0  0  0  0  0  0
   -6.1411    0.8320    0.1044 C   0  0  0  0  0  0  0  0  0  0  0  0
   -5.0493    1.5775    0.5303 C   0  0  0  0  0  0  0  0  0  0  0  0
   -3.7723    1.0325    0.4767 C   0  0  0  0  0  0  0  0  0  0  0  0
    1.6139    1.4380   -0.2295 C   0  0  0  0  0  0  0  0  0  0  0  0
    2.8710    2.0213   -0.2683 C   0  0  0  0  0  0  0  0  0  0  0  0
    4.0049    1.2374   -0.1323 C   0  0  0  0  0  0  0  0  0  0  0  0
    5.2321    1.8031   -0.1715 O   0  0  0  0  0  0  0  0  0  0  0  0
    3.8770   -0.1468    0.0432 C   0  0  0  0  0  0  0  0  0  0  0  0
    5.0684   -0.8042    0.1598 O   0  0  0  0  0  0  0  0  0  0  0  0
    5.0259   -2.2163    0.3095 C   0  0  0  0  0  0  0  0  0  0  0  0
    2.6236   -0.7267    0.0823 C   0  0  0  0  0  0  0  0  0  0  0  0
   -1.1732    1.0116   -0.2045 H   0  0  0  0  0  0  0  0  0  0  0  0
    0.1989   -1.6897    0.1073 H   0  0  0  0  0  0  0  0  0  0  0  0
   -4.5233   -2.0303   -0.7377 H   0  0  0  0  0  0  0  0  0  0  0  0
   -6.8099   -1.0494   -0.6869 H   0  0  0  0  0  0  0  0  0  0  0  0
   -7.1336    1.2601    0.1382 H   0  0  0  0  0  0  0  0  0  0  0  0
   -5.1908    2.5818    0.9048 H   0  0  0  0  0  0  0  0  0  0  0  0
   -2.9326    1.6164    0.8259 H   0  0  0  0  0  0  0  0  0  0  0  0
    0.7419    2.0656   -0.3416 H   0  0  0  0  0  0  0  0  0  0  0  0
    2.9963    3.0857   -0.4067 H   0  0  0  0  0  0  0  0  0  0  0  0
```

5.8864	1.1025	-0.0700	H	0	0	0	0	0	0	0	0	0	0	0	0
6.0584	-2.5429	0.3738	H	0	0	0	0	0	0	0	0	0	0	0	0
4.5453	-2.6777	-0.5542	H	0	0	0	0	0	0	0	0	0	0	0	0
4.4954	-2.4884	1.2229	H	0	0	0	0	0	0	0	0	0	0	0	0
2.5140	-1.7932	0.2169	H	0	0	0	0	0	0	0	0	0	0	0	0

1	2	1	0	0	0	0
1	7	4	0	0	0	0
1	11	4	0	0	0	0
2	3	2	0	0	0	0
2	4	1	0	0	0	0
4	5	2	0	0	0	0
4	20	1	0	0	0	0
5	6	1	0	0	0	0
5	21	1	0	0	0	0
6	12	4	0	0	0	0
6	19	4	0	0	0	0
7	8	4	0	0	0	0
7	22	1	0	0	0	0
8	9	4	0	0	0	0
8	23	1	0	0	0	0
9	10	4	0	0	0	0
9	24	1	0	0	0	0
10	11	4	0	0	0	0
10	25	1	0	0	0	0
11	26	1	0	0	0	0
12	13	4	0	0	0	0
12	27	1	0	0	0	0
13	14	4	0	0	0	0
13	28	1	0	0	0	0
14	15	1	0	0	0	0
14	16	4	0	0	0	0
15	29	1	0	0	0	0
16	17	1	0	0	0	0
16	19	4	0	0	0	0

```
 17 18  1  0  0  0  0
 18 30  1  0  0  0  0
 18 31  1  0  0  0  0
 18 32  1  0  0  0  0
 19 33  1  0  0  0  0
M  END
$$$$
4_A


 32 33  0  0  0  0  0  0  0  0  0 V2000
   -3.5950   -0.2608    0.0308 C   0  0  0  0  0  0  0  0  0  0  0  0
   -2.2495   -0.9457    0.1296 C   0  0  0  0  0  0  0  0  0  0  0  0
   -2.2426   -2.1718    0.2562 O   0  0  0  0  0  0  0  0  0  0  0  0
   -1.0752   -0.1201    0.0443 C   0  0  0  0  0  0  0  0  0  0  0  0
    0.1709   -0.6769    0.1814 C   0  0  0  0  0  0  0  0  0  0  0  0
    1.4387   -0.0543    0.1044 C   0  0  0  0  0  0  0  0  0  0  0  0
   -4.6862   -1.0287   -0.3751 C   0  0  0  0  0  0  0  0  0  0  0  0
   -5.9496   -0.4650   -0.4802 C   0  0  0  0  0  0  0  0  0  0  0  0
   -6.1441    0.8753   -0.1605 C   0  0  0  0  0  0  0  0  0  0  0  0
   -5.0668    1.6433    0.2648 C   0  0  0  0  0  0  0  0  0  0  0  0
   -3.7991    1.0796    0.3558 C   0  0  0  0  0  0  0  0  0  0  0  0
    1.6260    1.3273   -0.1551 C   0  0  0  0  0  0  0  0  0  0  0  0
    2.8711    1.8832   -0.2174 C   0  0  0  0  0  0  0  0  0  0  0  0
    4.0853    1.1212   -0.0126 C   0  0  0  0  0  0  0  0  0  0  0  0
    5.2201    1.6378   -0.0399 O   0  0  0  0  0  0  0  0  0  0  0  0
    3.8660   -0.2969    0.2307 C   0  0  0  0  0  0  0  0  0  0  0  0
    4.9498   -1.1172    0.4730 O   0  0  0  0  0  0  0  0  0  0  0  0
    5.8452   -1.2106   -0.6286 C   0  0  0  0  0  0  0  0  0  0  0  0
    2.6135   -0.8330    0.2863 C   0  0  0  0  0  0  0  0  0  0  0  0
   -1.1930    0.9324   -0.1632 H   0  0  0  0  0  0  0  0  0  0  0  0
    0.1734   -1.7458    0.3769 H   0  0  0  0  0  0  0  0  0  0  0  0
   -4.5115   -2.0723   -0.5970 H   0  0  0  0  0  0  0  0  0  0  0  0
   -6.7855   -1.0698   -0.8071 H   0  0  0  0  0  0  0  0  0  0  0  0
```

```
  -7.1295    1.3161   -0.2359 H   0  0  0  0  0  0  0  0  0  0  0  0
  -5.2132    2.6821    0.5304 H   0  0  0  0  0  0  0  0  0  0  0  0
  -2.9718    1.6806    0.7056 H   0  0  0  0  0  0  0  0  0  0  0  0
   0.7581    1.9573   -0.3075 H   0  0  0  0  0  0  0  0  0  0  0  0
   3.0048    2.9407   -0.4070 H   0  0  0  0  0  0  0  0  0  0  0  0
   6.6489   -1.8758   -0.3179 H   0  0  0  0  0  0  0  0  0  0  0  0
   6.2424   -0.2290   -0.8784 H   0  0  0  0  0  0  0  0  0  0  0  0
   5.3293   -1.6413   -1.4924 H   0  0  0  0  0  0  0  0  0  0  0  0
   2.5237   -1.8945    0.4884 H   0  0  0  0  0  0  0  0  0  0  0  0
  1  2  1  0  0  0  0
  1  7  4  0  0  0  0
  1 11  4  0  0  0  0
  2  3  2  0  0  0  0
  2  4  1  0  0  0  0
  4  5  2  0  0  0  0
  4 20  1  0  0  0  0
  5  6  1  0  0  0  0
  5 21  1  0  0  0  0
  6 12  4  0  0  0  0
  6 19  4  0  0  0  0
  7  8  4  0  0  0  0
  7 22  1  0  0  0  0
  8  9  4  0  0  0  0
  8 23  1  0  0  0  0
  9 10  4  0  0  0  0
  9 24  1  0  0  0  0
 10 11  4  0  0  0  0
 10 25  1  0  0  0  0
 11 26  1  0  0  0  0
 12 13  4  0  0  0  0
 12 27  1  0  0  0  0
 13 14  4  0  0  0  0
 13 28  1  0  0  0  0
 14 15  1  0  0  0  0
```

```
 14 16  4  0  0  0  0
 16 17  1  0  0  0  0
 16 19  4  0  0  0  0
 17 18  1  0  0  0  0
 18 29  1  0  0  0  0
 18 30  1  0  0  0  0
 18 31  1  0  0  0  0
 19 32  1  0  0  0  0
M  END
$$$$
4_NR


 32 33  0  0  0  0  0  0  0  0  0 V2000
   -3.6205   -0.2632    0.0125 C   0  0  0  0  0  0  0  0  0  0  0  0
   -2.3012   -0.9638   -0.0417 C   0  0  0  0  0  0  0  0  0  0  0  0
   -2.2499   -2.1778   -0.0563 O   0  0  0  0  0  0  0  0  0  0  0  0
   -1.0665   -0.1427   -0.1013 C   0  0  0  0  0  0  0  0  0  0  0  0
    0.1303   -0.7465    0.0044 C   0  0  0  0  0  0  0  0  0  0  0  0
    1.4289   -0.1122   -0.0542 C   0  0  0  0  0  0  0  0  0  0  0  0
   -4.7569   -0.9915   -0.3415 C   0  0  0  0  0  0  0  0  0  0  0  0
   -6.0078   -0.3977   -0.2980 C   0  0  0  0  0  0  0  0  0  0  0  0
   -6.1360    0.9245    0.1177 C   0  0  0  0  0  0  0  0  0  0  0  0
   -5.0111    1.6499    0.4885 C   0  0  0  0  0  0  0  0  0  0  0  0
   -3.7544    1.0608    0.4296 C   0  0  0  0  0  0  0  0  0  0  0  0
    1.5905    1.2906   -0.2874 C   0  0  0  0  0  0  0  0  0  0  0  0
    2.8240    1.8437   -0.3304 C   0  0  0  0  0  0  0  0  0  0  0  0
    4.0348    1.0615   -0.1456 C   0  0  0  0  0  0  0  0  0  0  0  0
    5.1541    1.5874   -0.1816 O   0  0  0  0  0  0  0  0  0  0  0  0
    3.8420   -0.3750    0.0839 C   0  0  0  0  0  0  0  0  0  0  0  0
    4.8368   -1.2393    0.2687 O   0  0  0  0  0  0  0  0  0  0  0  0
    6.2266   -0.8694    0.2308 C   0  0  0  0  0  0  0  0  0  0  0  0
    2.5605   -0.9046    0.1223 C   0  0  0  0  0  0  0  0  0  0  0  0
   -1.1586    0.9215   -0.2540 H   0  0  0  0  0  0  0  0  0  0  0  0
```

```
   0.1204   -1.8211    0.1507 H   0  0  0  0  0  0  0  0  0  0  0  0
  -4.6336   -2.0208   -0.6470 H   0  0  0  0  0  0  0  0  0  0  0  0
  -6.8843   -0.9626   -0.5835 H   0  0  0  0  0  0  0  0  0  0  0  0
  -7.1129    1.3868    0.1560 H   0  0  0  0  0  0  0  0  0  0  0  0
  -5.1114    2.6725    0.8247 H   0  0  0  0  0  0  0  0  0  0  0  0
  -2.8900    1.6310    0.7389 H   0  0  0  0  0  0  0  0  0  0  0  0
   0.7195    1.9122   -0.4352 H   0  0  0  0  0  0  0  0  0  0  0  0
   2.9761    2.8994   -0.5059 H   0  0  0  0  0  0  0  0  0  0  0  0
   6.7522   -1.8048    0.3918 H   0  0  0  0  0  0  0  0  0  0  0  0
   6.4534   -0.1550    1.0157 H   0  0  0  0  0  0  0  0  0  0  0  0
   6.4814   -0.4411   -0.7336 H   0  0  0  0  0  0  0  0  0  0  0  0
   2.4602   -1.9675    0.2963 H   0  0  0  0  0  0  0  0  0  0  0  0
  1  2  1  0  0  0  0
  1  7  4  0  0  0  0
  1 11  4  0  0  0  0
  2  3  2  0  0  0  0
  2  4  1  0  0  0  0
  4  5  2  0  0  0  0
  4 20  1  0  0  0  0
  5  6  1  0  0  0  0
  5 21  1  0  0  0  0
  6 12  4  0  0  0  0
  6 19  4  0  0  0  0
  7  8  4  0  0  0  0
  7 22  1  0  0  0  0
  8  9  4  0  0  0  0
  8 23  1  0  0  0  0
  9 10  4  0  0  0  0
  9 24  1  0  0  0  0
 10 11  4  0  0  0  0
 10 25  1  0  0  0  0
 11 26  1  0  0  0  0
 12 13  4  0  0  0  0
 12 27  1  0  0  0  0
```

```
 13 14  4  0  0  0  0
 13 28  1  0  0  0  0
 14 15  1  0  0  0  0
 14 16  4  0  0  0  0
 16 17  1  0  0  0  0
 16 19  4  0  0  0  0
 17 18  1  0  0  0  0
 18 29  1  0  0  0  0
 18 30  1  0  0  0  0
 18 31  1  0  0  0  0
 19 32  1  0  0  0  0
M  END
$$$$
4_PR


 33 34  0  0  0  0  0  0  0  0  0 V2000
    3.5768    0.2655   -0.0101 C   0  0  0  0  0  0  0  0  0  0  0  0
    2.2423    0.8956   -0.0860 C   0  0  0  0  0  0  0  0  0  0  0  0
    2.0839    2.0949   -0.1574 O   0  0  0  0  0  0  0  0  0  0  0  0
    1.0182    0.0192   -0.1081 C   0  0  0  0  0  0  0  0  0  0  0  0
   -0.1966    0.6175   -0.0119 C   0  0  0  0  0  0  0  0  0  0  0  0
   -1.4652   -0.0335   -0.0452 C   0  0  0  0  0  0  0  0  0  0  0  0
    4.6860    1.0732   -0.2786 C   0  0  0  0  0  0  0  0  0  0  0  0
    5.9616    0.5421   -0.2098 C   0  0  0  0  0  0  0  0  0  0  0  0
    6.1403   -0.7933    0.1427 C   0  0  0  0  0  0  0  0  0  0  0  0
    5.0436   -1.5981    0.4256 C   0  0  0  0  0  0  0  0  0  0  0  0
    3.7618   -1.0737    0.3438 C   0  0  0  0  0  0  0  0  0  0  0  0
   -1.5975   -1.4535   -0.2022 C   0  0  0  0  0  0  0  0  0  0  0  0
   -2.8213   -2.0483   -0.2310 C   0  0  0  0  0  0  0  0  0  0  0  0
   -3.9723   -1.2472   -0.1057 C   0  0  0  0  0  0  0  0  0  0  0  0
   -5.1515   -1.8045   -0.1316 O   0  0  0  0  0  0  0  0  0  0  0  0
   -3.8630    0.1810    0.0486 C   0  0  0  0  0  0  0  0  0  0  0  0
   -5.0457    0.7766    0.1452 O   0  0  0  0  0  0  0  0  0  0  0  0
```

-5.0959	2.2103	0.2938	C	0	0	0	0	0	0	0	0	0	0	0	0
-2.6231	0.7655	0.0788	C	0	0	0	0	0	0	0	0	0	0	0	0
1.1317	-1.0460	-0.2351	H	0	0	0	0	0	0	0	0	0	0	0	0
-0.1895	1.6965	0.0959	H	0	0	0	0	0	0	0	0	0	0	0	0
4.5237	2.1086	-0.5413	H	0	0	0	0	0	0	0	0	0	0	0	0
6.8182	1.1640	-0.4274	H	0	0	0	0	0	0	0	0	0	0	0	0
7.1380	-1.2060	0.1997	H	0	0	0	0	0	0	0	0	0	0	0	0
5.1875	-2.6303	0.7105	H	0	0	0	0	0	0	0	0	0	0	0	0
2.9247	-1.7129	0.5864	H	0	0	0	0	0	0	0	0	0	0	0	0
-0.7134	-2.0639	-0.3008	H	0	0	0	0	0	0	0	0	0	0	0	0
-2.9489	-3.1146	-0.3485	H	0	0	0	0	0	0	0	0	0	0	0	0
-5.8496	-1.1354	-0.0386	H	0	0	0	0	0	0	0	0	0	0	0	0
-6.1486	2.4584	0.3435	H	0	0	0	0	0	0	0	0	0	0	0	0
-4.6349	2.6811	-0.5724	H	0	0	0	0	0	0	0	0	0	0	0	0
-4.5941	2.4976	1.2158	H	0	0	0	0	0	0	0	0	0	0	0	0
-2.5130	1.8335	0.1944	H	0	0	0	0	0	0	0	0	0	0	0	0

1	2	1	0	0	0	0
1	7	4	0	0	0	0
1	11	4	0	0	0	0
2	3	2	0	0	0	0
2	4	1	0	0	0	0
4	5	2	0	0	0	0
4	20	1	0	0	0	0
5	6	1	0	0	0	0
5	21	1	0	0	0	0
6	12	4	0	0	0	0
6	19	4	0	0	0	0
7	8	4	0	0	0	0
7	22	1	0	0	0	0
8	9	4	0	0	0	0
8	23	1	0	0	0	0
9	10	4	0	0	0	0
9	24	1	0	0	0	0
10	11	4	0	0	0	0

```
 10 25  1  0  0  0  0
 11 26  1  0  0  0  0
 12 13  4  0  0  0  0
 12 27  1  0  0  0  0
 13 14  4  0  0  0  0
 13 28  1  0  0  0  0
 14 15  1  0  0  0  0
 14 16  4  0  0  0  0
 15 29  1  0  0  0  0
 16 17  1  0  0  0  0
 16 19  4  0  0  0  0
 17 18  1  0  0  0  0
 18 30  1  0  0  0  0
 18 31  1  0  0  0  0
 18 32  1  0  0  0  0
 19 33  1  0  0  0  0
M  END
$$$$
4a


 31 32  0  0  0  0  0  0  0  0  0 V2000
    3.5946    0.2543   -0.0000 C   0  0  0  0  0  0  0  0  0  0  0  0
    2.2491    0.9327   -0.0000 C   0  0  0  0  0  0  0  0  0  0  0  0
    2.2027    2.1474    0.0000 O   0  0  0  0  0  0  0  0  0  0  0  0
    1.0617    0.0661   -0.0001 C   0  0  0  0  0  0  0  0  0  0  0  0
   -0.1543    0.6316    0.0001 C   0  0  0  0  0  0  0  0  0  0  0  0
   -1.4438   -0.0473    0.0001 C   0  0  0  0  0  0  0  0  0  0  0  0
    3.6483   -1.0747   -0.0004 N   0  0  0  0  0  0  0  0  0  0  0  0
    4.8596   -1.6166   -0.0003 C   0  0  0  0  0  0  0  0  0  0  0  0
    6.0107   -0.8352    0.0000 C   0  0  0  0  0  0  0  0  0  0  0  0
    5.9638    0.4937    0.0003 N   0  0  0  0  0  0  0  0  0  0  0  0
    4.7519    1.0356    0.0002 C   0  0  0  0  0  0  0  0  0  0  0  0
   -1.5734   -1.4368    0.0000 C   0  0  0  0  0  0  0  0  0  0  0  0
```

-2.8253	-2.0315	0.0000	C	0	0	0	0	0	0	0	0	0	0	0	0
-3.9671	-1.2467	0.0001	C	0	0	0	0	0	0	0	0	0	0	0	0
-5.1887	-1.8243	0.0002	O	0	0	0	0	0	0	0	0	0	0	0	0
-3.8531	0.1502	0.0002	C	0	0	0	0	0	0	0	0	0	0	0	0
-5.0518	0.8045	0.0002	O	0	0	0	0	0	0	0	0	0	0	0	0
-5.0240	2.2249	-0.0005	C	0	0	0	0	0	0	0	0	0	0	0	0
-2.6051	0.7423	0.0002	C	0	0	0	0	0	0	0	0	0	0	0	0
1.2233	-1.0000	-0.0003	H	0	0	0	0	0	0	0	0	0	0	0	0
-0.1826	1.7170	0.0003	H	0	0	0	0	0	0	0	0	0	0	0	0
4.9216	-2.6972	-0.0006	H	0	0	0	0	0	0	0	0	0	0	0	0
6.9903	-1.2954	0.0001	H	0	0	0	0	0	0	0	0	0	0	0	0
4.6710	2.1134	0.0004	H	0	0	0	0	0	0	0	0	0	0	0	0
-0.6932	-2.0630	-0.0000	H	0	0	0	0	0	0	0	0	0	0	0	0
-2.9410	-3.1059	-0.0000	H	0	0	0	0	0	0	0	0	0	0	0	0
-5.8504	-1.1236	-0.0001	H	0	0	0	0	0	0	0	0	0	0	0	0
-6.0604	2.5457	-0.0007	H	0	0	0	0	0	0	0	0	0	0	0	0
-4.5220	2.5970	-0.8946	H	0	0	0	0	0	0	0	0	0	0	0	0
-4.5222	2.5979	0.8933	H	0	0	0	0	0	0	0	0	0	0	0	0
-2.5052	1.8183	0.0002	H	0	0	0	0	0	0	0	0	0	0	0	0

1	2	1	0	0	0	0
1	7	4	0	0	0	0
1	11	4	0	0	0	0
2	3	2	0	0	0	0
2	4	1	0	0	0	0
4	5	2	0	0	0	0
4	20	1	0	0	0	0
5	6	1	0	0	0	0
5	21	1	0	0	0	0
6	12	4	0	0	0	0
6	19	4	0	0	0	0
7	8	4	0	0	0	0
8	9	4	0	0	0	0
8	22	1	0	0	0	0
9	10	4	0	0	0	0

```
  9 23  1  0  0  0  0
 10 11  4  0  0  0  0
 11 24  1  0  0  0  0
 12 13  4  0  0  0  0
 12 25  1  0  0  0  0
 13 14  4  0  0  0  0
 13 26  1  0  0  0  0
 14 15  1  0  0  0  0
 14 16  4  0  0  0  0
 15 27  1  0  0  0  0
 16 17  1  0  0  0  0
 16 19  4  0  0  0  0
 17 18  1  0  0  0  0
 18 28  1  0  0  0  0
 18 29  1  0  0  0  0
 18 30  1  0  0  0  0
 19 31  1  0  0  0  0
M  END
$$$$
4a_A


 30 31  0  0  0  0  0  0  0  0  0 V2000
   -3.6004   -0.2261    0.0235 C   0  0  0  0  0  0  0  0  0  0  0  0
   -2.2615   -0.9365   -0.0366 C   0  0  0  0  0  0  0  0  0  0  0  0
   -2.2944   -2.1699   -0.1042 O   0  0  0  0  0  0  0  0  0  0  0  0
   -1.0964   -0.1141   -0.0198 C   0  0  0  0  0  0  0  0  0  0  0  0
    0.1493   -0.6947   -0.0895 C   0  0  0  0  0  0  0  0  0  0  0  0
    1.4150   -0.0739   -0.1000 C   0  0  0  0  0  0  0  0  0  0  0  0
   -3.6634    1.0943    0.1731 N   0  0  0  0  0  0  0  0  0  0  0  0
   -4.8779    1.6319    0.2174 C   0  0  0  0  0  0  0  0  0  0  0  0
   -6.0290    0.8649    0.1090 C   0  0  0  0  0  0  0  0  0  0  0  0
   -5.9812   -0.4579   -0.0439 N   0  0  0  0  0  0  0  0  0  0  0  0
   -4.7680   -0.9943   -0.0831 C   0  0  0  0  0  0  0  0  0  0  0  0
```

```
  1.6037    1.3324   -0.0423 C   0  0  0  0  0  0  0  0  0  0  0  0
  2.8487    1.8886   -0.0623 C   0  0  0  0  0  0  0  0  0  0  0  0
  4.0616    1.1011   -0.1572 C   0  0  0  0  0  0  0  0  0  0  0  0
  5.1956    1.6137   -0.2108 O   0  0  0  0  0  0  0  0  0  0  0  0
  3.8416   -0.3389   -0.1918 C   0  0  0  0  0  0  0  0  0  0  0  0
  4.9244   -1.1848   -0.3175 O   0  0  0  0  0  0  0  0  0  0  0  0
  5.8288   -1.1168    0.7792 C   0  0  0  0  0  0  0  0  0  0  0  0
  2.5891   -0.8745   -0.1692 C   0  0  0  0  0  0  0  0  0  0  0  0
 -1.2355    0.9530    0.0421 H   0  0  0  0  0  0  0  0  0  0  0  0
  0.1474   -1.7800   -0.1456 H   0  0  0  0  0  0  0  0  0  0  0  0
 -4.9383    2.7065    0.3422 H   0  0  0  0  0  0  0  0  0  0  0  0
 -7.0085    1.3255    0.1459 H   0  0  0  0  0  0  0  0  0  0  0  0
 -4.6781   -2.0642   -0.2028 H   0  0  0  0  0  0  0  0  0  0  0  0
  0.7352    1.9767    0.0141 H   0  0  0  0  0  0  0  0  0  0  0  0
  2.9859    2.9619   -0.0308 H   0  0  0  0  0  0  0  0  0  0  0  0
  6.6240   -1.8292    0.5675 H   0  0  0  0  0  0  0  0  0  0  0  0
  5.3167   -1.4059    1.7021 H   0  0  0  0  0  0  0  0  0  0  0  0
  6.2362   -0.1124    0.8738 H   0  0  0  0  0  0  0  0  0  0  0  0
  2.4956   -1.9536   -0.2174 H   0  0  0  0  0  0  0  0  0  0  0  0
1  2  1  0  0  0  0
1  7  4  0  0  0  0
1 11  4  0  0  0  0
2  3  2  0  0  0  0
2  4  1  0  0  0  0
4  5  2  0  0  0  0
4 20  1  0  0  0  0
5  6  1  0  0  0  0
5 21  1  0  0  0  0
6 12  4  0  0  0  0
6 19  4  0  0  0  0
7  8  4  0  0  0  0
8  9  4  0  0  0  0
8 22  1  0  0  0  0
9 10  4  0  0  0  0
```

9 23 1 0 0 0 0
10 11 4 0 0 0 0
11 24 1 0 0 0 0
12 13 4 0 0 0 0
12 25 1 0 0 0 0
13 14 4 0 0 0 0
13 26 1 0 0 0 0
14 15 1 0 0 0 0
14 16 4 0 0 0 0
16 17 1 0 0 0 0
16 19 4 0 0 0 0
17 18 1 0 0 0 0
18 27 1 0 0 0 0
18 28 1 0 0 0 0
18 29 1 0 0 0 0
19 30 1 0 0 0 0
M END
$$$$
4a_NR

30 31 0 0 0 0 0 0 0 0 0 V2000
3.6307 -0.2503 -0.0000 C 0 0 0 0 0 0 0 0 0 0 0 0
2.3123 -0.9742 0.0000 C 0 0 0 0 0 0 0 0 0 0 0 0
2.2984 -2.1884 0.0000 O 0 0 0 0 0 0 0 0 0 0 0 0
1.0901 -0.1506 0.0000 C 0 0 0 0 0 0 0 0 0 0 0 0
-0.1084 -0.7642 0.0000 C 0 0 0 0 0 0 0 0 0 0 0 0
-1.4023 -0.1220 0.0000 C 0 0 0 0 0 0 0 0 0 0 0 0
3.6349 1.0800 -0.0000 N 0 0 0 0 0 0 0 0 0 0 0 0
4.8253 1.6659 -0.0000 C 0 0 0 0 0 0 0 0 0 0 0 0
6.0039 0.9258 -0.0000 C 0 0 0 0 0 0 0 0 0 0 0 0
6.0053 -0.4036 0.0000 N 0 0 0 0 0 0 0 0 0 0 0 0
4.8143 -0.9900 0.0000 C 0 0 0 0 0 0 0 0 0 0 0 0
-1.5505 1.3021 0.0000 C 0 0 0 0 0 0 0 0 0 0 0 0

```
-2.7799    1.8648    0.0000 C   0  0  0  0  0  0  0  0  0  0  0  0
-3.9990    1.0726    0.0000 C   0  0  0  0  0  0  0  0  0  0  0  0
-5.1146    1.6057   -0.0000 O   0  0  0  0  0  0  0  0  0  0  0  0
-3.8187   -0.3847   -0.0000 C   0  0  0  0  0  0  0  0  0  0  0  0
-4.8222   -1.2585   -0.0000 O   0  0  0  0  0  0  0  0  0  0  0  0
-6.2091   -0.8755   -0.0000 C   0  0  0  0  0  0  0  0  0  0  0  0
-2.5413   -0.9242   -0.0000 C   0  0  0  0  0  0  0  0  0  0  0  0
 1.2134    0.9204    0.0000 H   0  0  0  0  0  0  0  0  0  0  0  0
-0.1044   -1.8489    0.0000 H   0  0  0  0  0  0  0  0  0  0  0  0
 4.8481    2.7478   -0.0000 H   0  0  0  0  0  0  0  0  0  0  0  0
 6.9663    1.4211   -0.0000 H   0  0  0  0  0  0  0  0  0  0  0  0
 4.7738   -2.0701    0.0000 H   0  0  0  0  0  0  0  0  0  0  0  0
-0.6714    1.9297    0.0000 H   0  0  0  0  0  0  0  0  0  0  0  0
-2.9230    2.9362    0.0000 H   0  0  0  0  0  0  0  0  0  0  0  0
-6.7426   -1.8202   -0.0000 H   0  0  0  0  0  0  0  0  0  0  0  0
-6.4449   -0.2952   -0.8865 H   0  0  0  0  0  0  0  0  0  0  0  0
-6.4449   -0.2952    0.8864 H   0  0  0  0  0  0  0  0  0  0  0  0
-2.4496   -2.0020   -0.0000 H   0  0  0  0  0  0  0  0  0  0  0  0
1  2  1  0  0  0  0
1  7  4  0  0  0  0
1 11  4  0  0  0  0
2  3  2  0  0  0  0
2  4  1  0  0  0  0
4  5  2  0  0  0  0
4 20  1  0  0  0  0
5  6  1  0  0  0  0
5 21  1  0  0  0  0
6 12  4  0  0  0  0
6 19  4  0  0  0  0
7  8  4  0  0  0  0
8  9  4  0  0  0  0
8 22  1  0  0  0  0
9 10  4  0  0  0  0
9 23  1  0  0  0  0
```

```
 10 11  4  0  0  0  0
 11 24  1  0  0  0  0
 12 13  4  0  0  0  0
 12 25  1  0  0  0  0
 13 14  4  0  0  0  0
 13 26  1  0  0  0  0
 14 15  1  0  0  0  0
 14 16  4  0  0  0  0
 16 17  1  0  0  0  0
 16 19  4  0  0  0  0
 17 18  1  0  0  0  0
 18 27  1  0  0  0  0
 18 28  1  0  0  0  0
 18 29  1  0  0  0  0
 19 30  1  0  0  0  0
M  END
$$$$
4a_PR


 31 32  0  0  0  0  0  0  0  0  0 V2000
   -3.5844    0.2860   -0.0001 C   0  0  0  0  0  0  0  0  0  0  0  0
   -2.2612    0.9695   -0.0001 C   0  0  0  0  0  0  0  0  0  0  0  0
   -2.1540    2.1731    0.0000 O   0  0  0  0  0  0  0  0  0  0  0  0
   -1.0466    0.0933   -0.0000 C   0  0  0  0  0  0  0  0  0  0  0  0
    0.1726    0.6873   -0.0000 C   0  0  0  0  0  0  0  0  0  0  0  0
    1.4291    0.0093    0.0000 C   0  0  0  0  0  0  0  0  0  0  0  0
   -3.5931   -1.0474   -0.0001 N   0  0  0  0  0  0  0  0  0  0  0  0
   -4.7844   -1.6250   -0.0001 C   0  0  0  0  0  0  0  0  0  0  0  0
   -5.9593   -0.8697   -0.0001 C   0  0  0  0  0  0  0  0  0  0  0  0
   -5.9503    0.4575   -0.0001 N   0  0  0  0  0  0  0  0  0  0  0  0
   -4.7566    1.0392   -0.0001 C   0  0  0  0  0  0  0  0  0  0  0  0
    1.5211   -1.4231   -0.0000 C   0  0  0  0  0  0  0  0  0  0  0  0
    2.7281   -2.0513    0.0000 C   0  0  0  0  0  0  0  0  0  0  0  0
```

3.9018	-1.2730	0.0000	C	0	0	0	0	0	0	0	0	0	0	0	0
5.0658	-1.8610	0.0000	O	0	0	0	0	0	0	0	0	0	0	0	0
3.8317	0.1669	0.0000	C	0	0	0	0	0	0	0	0	0	0	0	0
5.0299	0.7366	0.0001	O	0	0	0	0	0	0	0	0	0	0	0	0
5.1200	2.1766	0.0002	C	0	0	0	0	0	0	0	0	0	0	0	0
2.6074	0.7858	0.0000	C	0	0	0	0	0	0	0	0	0	0	0	0
-1.2076	-0.9732	-0.0000	H	0	0	0	0	0	0	0	0	0	0	0	0
0.1868	1.7719	-0.0000	H	0	0	0	0	0	0	0	0	0	0	0	0
-4.8184	-2.7061	-0.0001	H	0	0	0	0	0	0	0	0	0	0	0	0
-6.9256	-1.3570	-0.0001	H	0	0	0	0	0	0	0	0	0	0	0	0
-4.7140	2.1199	-0.0000	H	0	0	0	0	0	0	0	0	0	0	0	0
0.6179	-2.0137	-0.0000	H	0	0	0	0	0	0	0	0	0	0	0	0
2.8259	-3.1271	-0.0000	H	0	0	0	0	0	0	0	0	0	0	0	0
5.7825	-1.2055	0.0001	H	0	0	0	0	0	0	0	0	0	0	0	0
6.1797	2.3989	0.0003	H	0	0	0	0	0	0	0	0	0	0	0	0
4.6491	2.5692	0.8993	H	0	0	0	0	0	0	0	0	0	0	0	0
4.6492	2.5693	-0.8990	H	0	0	0	0	0	0	0	0	0	0	0	0
2.5278	1.8628	0.0000	H	0	0	0	0	0	0	0	0	0	0	0	0

1	2	1	0	0	0	0
1	7	4	0	0	0	0
1	11	4	0	0	0	0
2	3	2	0	0	0	0
2	4	1	0	0	0	0
4	5	2	0	0	0	0
4	20	1	0	0	0	0
5	6	1	0	0	0	0
5	21	1	0	0	0	0
6	12	4	0	0	0	0
6	19	4	0	0	0	0
7	8	4	0	0	0	0
8	9	4	0	0	0	0
8	22	1	0	0	0	0
9	10	4	0	0	0	0
9	23	1	0	0	0	0

```
 10 11  4  0  0  0  0
 11 24  1  0  0  0  0
 12 13  4  0  0  0  0
 12 25  1  0  0  0  0
 13 14  4  0  0  0  0
 13 26  1  0  0  0  0
 14 15  1  0  0  0  0
 14 16  4  0  0  0  0
 15 27  1  0  0  0  0
 16 17  1  0  0  0  0
 16 19  4  0  0  0  0
 17 18  1  0  0  0  0
 18 28  1  0  0  0  0
 18 29  1  0  0  0  0
 18 30  1  0  0  0  0
 19 31  1  0  0  0  0
M  END
$$$$
```

4e

```
 43 44  0  0  0  0  0  0  0  0  0 V2000
    1.9228   -0.8126    0.2727 C   0  0  0  0  0  0  0  0  0  0  0  0
    0.5245   -1.3277    0.0724 C   0  0  0  0  0  0  0  0  0  0  0  0
    0.3543   -2.5147   -0.1304 O   0  0  0  0  0  0  0  0  0  0  0  0
   -0.5696   -0.3452    0.1377 C   0  0  0  0  0  0  0  0  0  0  0  0
   -1.8308   -0.7702   -0.0226 C   0  0  0  0  0  0  0  0  0  0  0  0
   -3.0467    0.0335    0.0066 C   0  0  0  0  0  0  0  0  0  0  0  0
    2.1130    0.4864    0.4891 N   0  0  0  0  0  0  0  0  0  0  0  0
    3.3659    0.8819    0.6607 C   0  0  0  0  0  0  0  0  0  0  0  0
    4.4449   -0.0053    0.6195 C   0  0  0  0  0  0  0  0  0  0  0  0
    4.2476   -1.3068    0.4008 N   0  0  0  0  0  0  0  0  0  0  0  0
    2.9940   -1.7054    0.2304 C   0  0  0  0  0  0  0  0  0  0  0  0
   -3.0429    1.4185    0.1780 C   0  0  0  0  0  0  0  0  0  0  0  0
```

-4.2309	2.1325	0.2007	C	0	0	0	0	0	0	0	0	0	0	0	0
-5.4406	1.4734	0.0532	C	0	0	0	0	0	0	0	0	0	0	0	0
-6.6003	2.1673	0.0759	O	0	0	0	0	0	0	0	0	0	0	0	0
-5.4602	0.0831	-0.1213	C	0	0	0	0	0	0	0	0	0	0	0	0
-6.7141	-0.4420	-0.2554	O	0	0	0	0	0	0	0	0	0	0	0	0
-6.8204	-1.8486	-0.4217	C	0	0	0	0	0	0	0	0	0	0	0	0
-4.2759	-0.6278	-0.1453	C	0	0	0	0	0	0	0	0	0	0	0	0
5.8610	0.4574	0.7851	C	0	0	0	0	0	0	0	0	0	0	0	0
6.6595	0.3537	-0.5246	C	0	0	0	0	0	0	0	0	0	0	0	0
6.0767	1.2716	-1.5968	C	0	0	0	0	0	0	0	0	0	0	0	0
8.1254	0.6899	-0.2655	C	0	0	0	0	0	0	0	0	0	0	0	0
-0.3050	0.6847	0.3172	H	0	0	0	0	0	0	0	0	0	0	0	0
-1.9639	-1.8345	-0.1912	H	0	0	0	0	0	0	0	0	0	0	0	0
3.5340	1.9374	0.8363	H	0	0	0	0	0	0	0	0	0	0	0	0
5.8767	1.4916	1.1386	H	0	0	0	0	0	0	0	0	0	0	0	0
6.1094	2.3123	-1.2628	H	0	0	0	0	0	0	0	0	0	0	0	0
5.0403	1.0206	-1.8253	H	0	0	0	0	0	0	0	0	0	0	0	0
6.6517	1.1981	-2.5202	H	0	0	0	0	0	0	0	0	0	0	0	0
6.5851	-0.6817	-0.8651	H	0	0	0	0	0	0	0	0	0	0	0	0
8.5588	0.0217	0.4796	H	0	0	0	0	0	0	0	0	0	0	0	0
8.2222	1.7153	0.0994	H	0	0	0	0	0	0	0	0	0	0	0	0
8.7098	0.6042	-1.1821	H	0	0	0	0	0	0	0	0	0	0	0	0
6.3355	-0.1678	1.5437	H	0	0	0	0	0	0	0	0	0	0	0	0
2.8093	-2.7561	0.0551	H	0	0	0	0	0	0	0	0	0	0	0	0
-2.1080	1.9469	0.2929	H	0	0	0	0	0	0	0	0	0	0	0	0
-4.2430	3.2049	0.3323	H	0	0	0	0	0	0	0	0	0	0	0	0
-7.3244	1.5419	-0.0414	H	0	0	0	0	0	0	0	0	0	0	0	0
-7.8808	-2.0626	-0.5022	H	0	0	0	0	0	0	0	0	0	0	0	0
-6.4027	-2.3675	0.4421	H	0	0	0	0	0	0	0	0	0	0	0	0
-6.3095	-2.1658	-1.3318	H	0	0	0	0	0	0	0	0	0	0	0	0
-4.2791	-1.6999	-0.2800	H	0	0	0	0	0	0	0	0	0	0	0	0

1 2 1 0 0 0 0
1 7 4 0 0 0 0
1 11 4 0 0 0 0

2	3	2	0	0	0	0
2	4	1	0	0	0	0
4	5	2	0	0	0	0
4	24	1	0	0	0	0
5	6	1	0	0	0	0
5	25	1	0	0	0	0
6	12	4	0	0	0	0
6	19	4	0	0	0	0
7	8	4	0	0	0	0
8	9	4	0	0	0	0
8	26	1	0	0	0	0
9	10	4	0	0	0	0
9	20	1	0	0	0	0
10	11	4	0	0	0	0
11	36	1	0	0	0	0
12	13	4	0	0	0	0
12	37	1	0	0	0	0
13	14	4	0	0	0	0
13	38	1	0	0	0	0
14	15	1	0	0	0	0
14	16	4	0	0	0	0
15	39	1	0	0	0	0
16	17	1	0	0	0	0
16	19	4	0	0	0	0
17	18	1	0	0	0	0
18	40	1	0	0	0	0
18	41	1	0	0	0	0
18	42	1	0	0	0	0
19	43	1	0	0	0	0
20	21	1	0	0	0	0
20	27	1	0	0	0	0
20	35	1	0	0	0	0
21	22	1	0	0	0	0
21	23	1	0	0	0	0

```
 21 31  1  0  0  0  0
 22 28  1  0  0  0  0
 22 29  1  0  0  0  0
 22 30  1  0  0  0  0
 23 32  1  0  0  0  0
 23 33  1  0  0  0  0
 23 34  1  0  0  0  0
M  END
$$$$
4e_A


 42 43  0  0  0  0  0  0  0  0  0 V2000
    1.9216    0.7790   -0.3268 C   0  0  0  0  0  0  0  0  0  0  0  0
    0.5233    1.3420   -0.1655 C   0  0  0  0  0  0  0  0  0  0  0  0
    0.4371    2.5607    0.0203 O   0  0  0  0  0  0  0  0  0  0  0  0
   -0.5585    0.4147   -0.2434 C   0  0  0  0  0  0  0  0  0  0  0  0
   -1.8511    0.8638   -0.1047 C   0  0  0  0  0  0  0  0  0  0  0  0
   -3.0537    0.1274   -0.1421 C   0  0  0  0  0  0  0  0  0  0  0  0
    2.1170   -0.5226   -0.5170 N   0  0  0  0  0  0  0  0  0  0  0  0
    3.3751   -0.9277   -0.6460 C   0  0  0  0  0  0  0  0  0  0  0  0
    4.4575   -0.0544   -0.5887 C   0  0  0  0  0  0  0  0  0  0  0  0
    4.2643    1.2540   -0.3983 N   0  0  0  0  0  0  0  0  0  0  0  0
    3.0085    1.6607   -0.2707 C   0  0  0  0  0  0  0  0  0  0  0  0
   -3.1118   -1.2776   -0.3404 C   0  0  0  0  0  0  0  0  0  0  0  0
   -4.2992   -1.9474   -0.3690 C   0  0  0  0  0  0  0  0  0  0  0  0
   -5.5800   -1.2872   -0.2151 C   0  0  0  0  0  0  0  0  0  0  0  0
   -6.6646   -1.8976   -0.2720 O   0  0  0  0  0  0  0  0  0  0  0  0
   -5.4916    0.1499    0.0075 C   0  0  0  0  0  0  0  0  0  0  0  0
   -6.6515    0.8868    0.1329 O   0  0  0  0  0  0  0  0  0  0  0  0
   -7.4298    0.5292    1.2692 C   0  0  0  0  0  0  0  0  0  0  0  0
   -4.2947    0.8000    0.0347 C   0  0  0  0  0  0  0  0  0  0  0  0
    5.8771   -0.5305   -0.7057 C   0  0  0  0  0  0  0  0  0  0  0  0
    6.6549   -0.3754    0.6093 C   0  0  0  0  0  0  0  0  0  0  0  0
```

6.0524	-1.2475	1.7081	C	0	0	0	0	0	0	0	0	0	0	0	0
8.1254	-0.7228	0.3917	C	0	0	0	0	0	0	0	0	0	0	0	0
-0.3205	-0.6236	-0.4095	H	0	0	0	0	0	0	0	0	0	0	0	0
-1.9498	1.9339	0.0568	H	0	0	0	0	0	0	0	0	0	0	0	0
3.5380	-1.9888	-0.7993	H	0	0	0	0	0	0	0	0	0	0	0	0
5.8925	-1.5803	-1.0124	H	0	0	0	0	0	0	0	0	0	0	0	0
6.0918	-2.3014	1.4185	H	0	0	0	0	0	0	0	0	0	0	0	0
5.0110	-0.9882	1.8983	H	0	0	0	0	0	0	0	0	0	0	0	0
6.6069	-1.1338	2.6408	H	0	0	0	0	0	0	0	0	0	0	0	0
6.5747	0.6728	0.9063	H	0	0	0	0	0	0	0	0	0	0	0	0
8.5717	-0.0880	-0.3752	H	0	0	0	0	0	0	0	0	0	0	0	0
8.2258	-1.7633	0.0715	H	0	0	0	0	0	0	0	0	0	0	0	0
8.6964	-0.5993	1.3132	H	0	0	0	0	0	0	0	0	0	0	0	0
6.3723	0.0546	-1.4837	H	0	0	0	0	0	0	0	0	0	0	0	0
2.8189	2.7134	-0.1180	H	0	0	0	0	0	0	0	0	0	0	0	0
-2.1882	-1.8269	-0.4740	H	0	0	0	0	0	0	0	0	0	0	0	0
-4.3374	-3.0171	-0.5306	H	0	0	0	0	0	0	0	0	0	0	0	0
-8.3065	1.1743	1.2582	H	0	0	0	0	0	0	0	0	0	0	0	0
-6.8569	0.7084	2.1842	H	0	0	0	0	0	0	0	0	0	0	0	0
-7.7286	-0.5153	1.2106	H	0	0	0	0	0	0	0	0	0	0	0	0
-4.3018	1.8735	0.1874	H	0	0	0	0	0	0	0	0	0	0	0	0

1	2	1	0	0	0	0
1	7	4	0	0	0	0
1	11	4	0	0	0	0
2	3	2	0	0	0	0
2	4	1	0	0	0	0
4	5	2	0	0	0	0
4	24	1	0	0	0	0
5	6	1	0	0	0	0
5	25	1	0	0	0	0
6	12	4	0	0	0	0
6	19	4	0	0	0	0
7	8	4	0	0	0	0
8	9	4	0	0	0	0

```
  8 26  1  0  0  0  0
  9 10  4  0  0  0  0
  9 20  1  0  0  0  0
 10 11  4  0  0  0  0
 11 36  1  0  0  0  0
 12 13  4  0  0  0  0
 12 37  1  0  0  0  0
 13 14  4  0  0  0  0
 13 38  1  0  0  0  0
 14 15  1  0  0  0  0
 14 16  4  0  0  0  0
 16 17  1  0  0  0  0
 16 19  4  0  0  0  0
 17 18  1  0  0  0  0
 18 39  1  0  0  0  0
 18 40  1  0  0  0  0
 18 41  1  0  0  0  0
 19 42  1  0  0  0  0
 20 21  1  0  0  0  0
 20 27  1  0  0  0  0
 20 35  1  0  0  0  0
 21 22  1  0  0  0  0
 21 23  1  0  0  0  0
 21 31  1  0  0  0  0
 22 28  1  0  0  0  0
 22 29  1  0  0  0  0
 22 30  1  0  0  0  0
 23 32  1  0  0  0  0
 23 33  1  0  0  0  0
 23 34  1  0  0  0  0
M  END
$$$$
4e_NR
```

```
42 43  0  0  0  0  0  0  0  0  0 V2000
   1.9482    0.8779   -0.2501 C   0  0  0  0  0  0  0  0  0  0  0  0
   0.5692    1.4305   -0.0389 C   0  0  0  0  0  0  0  0  0  0  0  0
   0.4248    2.6138    0.1956 O   0  0  0  0  0  0  0  0  0  0  0  0
  -0.5582    0.4833   -0.1313 C   0  0  0  0  0  0  0  0  0  0  0  0
  -1.8108    0.9416    0.0490 C   0  0  0  0  0  0  0  0  0  0  0  0
  -3.0268    0.1630   -0.0042 C   0  0  0  0  0  0  0  0  0  0  0  0
   2.0941   -0.4222   -0.4955 N   0  0  0  0  0  0  0  0  0  0  0  0
   3.3325   -0.8559   -0.6749 C   0  0  0  0  0  0  0  0  0  0  0  0
   4.4412   -0.0058   -0.6138 C   0  0  0  0  0  0  0  0  0  0  0  0
   4.2871    1.2963   -0.3664 N   0  0  0  0  0  0  0  0  0  0  0  0
   3.0477    1.7335   -0.1870 C   0  0  0  0  0  0  0  0  0  0  0  0
  -3.0234   -1.2463   -0.2563 C   0  0  0  0  0  0  0  0  0  0  0  0
  -4.1838   -1.9393   -0.2985 C   0  0  0  0  0  0  0  0  0  0  0  0
  -5.4767   -1.3071   -0.0942 C   0  0  0  0  0  0  0  0  0  0  0  0
  -6.5270   -1.9588   -0.1353 O   0  0  0  0  0  0  0  0  0  0  0  0
  -5.4518    0.1384    0.1617 C   0  0  0  0  0  0  0  0  0  0  0  0
  -6.5398    0.8765    0.3694 O   0  0  0  0  0  0  0  0  0  0  0  0
  -7.8752    0.3417    0.3627 C   0  0  0  0  0  0  0  0  0  0  0  0
  -4.2417    0.8142    0.1983 C   0  0  0  0  0  0  0  0  0  0  0  0
   5.8403   -0.5121   -0.7898 C   0  0  0  0  0  0  0  0  0  0  0  0
   6.6406   -0.4678    0.5228 C   0  0  0  0  0  0  0  0  0  0  0  0
   6.0254   -1.3904    1.5725 C   0  0  0  0  0  0  0  0  0  0  0  0
   8.0935   -0.8500    0.2539 C   0  0  0  0  0  0  0  0  0  0  0  0
  -0.3230   -0.5476   -0.3422 H   0  0  0  0  0  0  0  0  0  0  0  0
  -1.9206    2.0012    0.2541 H   0  0  0  0  0  0  0  0  0  0  0  0
   3.4649   -1.9123   -0.8737 H   0  0  0  0  0  0  0  0  0  0  0  0
   5.8217   -1.5373   -1.1680 H   0  0  0  0  0  0  0  0  0  0  0  0
   6.0177   -2.4226    1.2121 H   0  0  0  0  0  0  0  0  0  0  0  0
   5.0000   -1.1062    1.8116 H   0  0  0  0  0  0  0  0  0  0  0  0
   6.6050   -1.3625    2.4956 H   0  0  0  0  0  0  0  0  0  0  0  0
   6.6034    0.5610    0.8881 H   0  0  0  0  0  0  0  0  0  0  0  0
   8.5493   -0.1827   -0.4784 H   0  0  0  0  0  0  0  0  0  0  0  0
```

8.1534 -1.8707 -0.1317 H 0 0 0 0 0 0 0 0 0 0 0 0
8.6820 -0.8034 1.1706 H 0 0 0 0 0 0 0 0 0 0 0 0
6.3366 0.1152 -1.5324 H 0 0 0 0 0 0 0 0 0 0 0 0
2.8995 2.7858 0.0118 H 0 0 0 0 0 0 0 0 0 0 0 0
-2.0849 -1.7574 -0.4129 H 0 0 0 0 0 0 0 0 0 0 0 0
-4.2125 -3.0035 -0.4860 H 0 0 0 0 0 0 0 0 0 0 0 0
-8.5048 1.2042 0.5548 H 0 0 0 0 0 0 0 0 0 0 0 0
-7.9913 -0.4056 1.1414 H 0 0 0 0 0 0 0 0 0 0 0 0
-8.1049 -0.1009 -0.6014 H 0 0 0 0 0 0 0 0 0 0 0 0
-4.2648 1.8785 0.3902 H 0 0 0 0 0 0 0 0 0 0 0 0
1 2 1 0 0 0 0
1 7 4 0 0 0 0
1 11 4 0 0 0 0
2 3 2 0 0 0 0
2 4 1 0 0 0 0
4 5 2 0 0 0 0
4 24 1 0 0 0 0
5 6 1 0 0 0 0
5 25 1 0 0 0 0
6 12 4 0 0 0 0
6 19 4 0 0 0 0
7 8 4 0 0 0 0
8 9 4 0 0 0 0
8 26 1 0 0 0 0
9 10 4 0 0 0 0
9 20 1 0 0 0 0
10 11 4 0 0 0 0
11 36 1 0 0 0 0
12 13 4 0 0 0 0
12 37 1 0 0 0 0
13 14 4 0 0 0 0
13 38 1 0 0 0 0
14 15 1 0 0 0 0
14 16 4 0 0 0 0

```
 16 17  1  0  0  0  0
 16 19  4  0  0  0  0
 17 18  1  0  0  0  0
 18 39  1  0  0  0  0
 18 40  1  0  0  0  0
 18 41  1  0  0  0  0
 19 42  1  0  0  0  0
 20 21  1  0  0  0  0
 20 27  1  0  0  0  0
 20 35  1  0  0  0  0
 21 22  1  0  0  0  0
 21 23  1  0  0  0  0
 21 31  1  0  0  0  0
 22 28  1  0  0  0  0
 22 29  1  0  0  0  0
 22 30  1  0  0  0  0
 23 32  1  0  0  0  0
 23 33  1  0  0  0  0
 23 34  1  0  0  0  0
M  END
$$$$
4e_PR


 43 44  0  0  0  0  0  0  0  0  0 V2000
    1.9176   -0.8883    0.2642 C   0  0  0  0  0  0  0  0  0  0  0  0
    0.5387   -1.3990    0.0701 C   0  0  0  0  0  0  0  0  0  0  0  0
    0.2951   -2.5669   -0.1304 O   0  0  0  0  0  0  0  0  0  0  0  0
   -0.5705   -0.3929    0.1349 C   0  0  0  0  0  0  0  0  0  0  0  0
   -1.8442   -0.8355   -0.0178 C   0  0  0  0  0  0  0  0  0  0  0  0
   -3.0186   -0.0261    0.0158 C   0  0  0  0  0  0  0  0  0  0  0  0
    2.0758    0.4198    0.4811 N   0  0  0  0  0  0  0  0  0  0  0  0
    3.3154    0.8398    0.6556 C   0  0  0  0  0  0  0  0  0  0  0  0
    4.4160   -0.0337    0.6182 C   0  0  0  0  0  0  0  0  0  0  0  0
```

4.2433	-1.3371	0.3974	N	0	0	0	0	0	0	0	0	0	0	0	0
3.0000	-1.7639	0.2224	C	0	0	0	0	0	0	0	0	0	0	0	0
-2.9612	1.3941	0.2125	C	0	0	0	0	0	0	0	0	0	0	0	0
-4.0942	2.1471	0.2404	C	0	0	0	0	0	0	0	0	0	0	0	0
-5.3393	1.5118	0.0728	C	0	0	0	0	0	0	0	0	0	0	0	0
-6.4346	2.2204	0.0978	O	0	0	0	0	0	0	0	0	0	0	0	0
-5.4196	0.0868	-0.1258	C	0	0	0	0	0	0	0	0	0	0	0	0
-6.6695	-0.3394	-0.2658	O	0	0	0	0	0	0	0	0	0	0	0	0
-6.9085	-1.7478	-0.4644	C	0	0	0	0	0	0	0	0	0	0	0	0
-4.2698	-0.6591	-0.1521	C	0	0	0	0	0	0	0	0	0	0	0	0
5.8170	0.4582	0.7872	C	0	0	0	0	0	0	0	0	0	0	0	0
6.6048	0.4053	-0.5360	C	0	0	0	0	0	0	0	0	0	0	0	0
5.9764	1.3186	-1.5861	C	0	0	0	0	0	0	0	0	0	0	0	0
8.0576	0.7947	-0.2795	C	0	0	0	0	0	0	0	0	0	0	0	0
-0.2954	0.6367	0.3028	H	0	0	0	0	0	0	0	0	0	0	0	0
-1.9724	-1.9007	-0.1780	H	0	0	0	0	0	0	0	0	0	0	0	0
3.4663	1.8972	0.8312	H	0	0	0	0	0	0	0	0	0	0	0	0
5.8120	1.4829	1.1654	H	0	0	0	0	0	0	0	0	0	0	0	0
5.9557	2.3514	-1.2283	H	0	0	0	0	0	0	0	0	0	0	0	0
4.9555	1.0205	-1.8305	H	0	0	0	0	0	0	0	0	0	0	0	0
6.5577	1.2973	-2.5076	H	0	0	0	0	0	0	0	0	0	0	0	0
6.5684	-0.6268	-0.8921	H	0	0	0	0	0	0	0	0	0	0	0	0
8.5212	0.1355	0.4547	H	0	0	0	0	0	0	0	0	0	0	0	0
8.1182	1.8191	0.0949	H	0	0	0	0	0	0	0	0	0	0	0	0
8.6378	0.7401	-1.2005	H	0	0	0	0	0	0	0	0	0	0	0	0
6.3117	-0.1777	1.5231	H	0	0	0	0	0	0	0	0	0	0	0	0
2.8423	-2.8194	0.0456	H	0	0	0	0	0	0	0	0	0	0	0	0
-2.0035	1.8746	0.3405	H	0	0	0	0	0	0	0	0	0	0	0	0
-4.0795	3.2172	0.3872	H	0	0	0	0	0	0	0	0	0	0	0	0
-7.2121	1.6526	-0.0303	H	0	0	0	0	0	0	0	0	0	0	0	0
-7.9829	-1.8488	-0.5541	H	0	0	0	0	0	0	0	0	0	0	0	0
-6.5456	-2.3023	0.3990	H	0	0	0	0	0	0	0	0	0	0	0	0
-6.4189	-2.0737	-1.3801	H	0	0	0	0	0	0	0	0	0	0	0	0
-4.3019	-1.7282	-0.2999	H	0	0	0	0	0	0	0	0	0	0	0	0

1	2	1	0	0	0	0
1	7	4	0	0	0	0
1	11	4	0	0	0	0
2	3	2	0	0	0	0
2	4	1	0	0	0	0
4	5	2	0	0	0	0
4	24	1	0	0	0	0
5	6	1	0	0	0	0
5	25	1	0	0	0	0
6	12	4	0	0	0	0
6	19	4	0	0	0	0
7	8	4	0	0	0	0
8	9	4	0	0	0	0
8	26	1	0	0	0	0
9	10	4	0	0	0	0
9	20	1	0	0	0	0
10	11	4	0	0	0	0
11	36	1	0	0	0	0
12	13	4	0	0	0	0
12	37	1	0	0	0	0
13	14	4	0	0	0	0
13	38	1	0	0	0	0
14	15	1	0	0	0	0
14	16	4	0	0	0	0
15	39	1	0	0	0	0
16	17	1	0	0	0	0
16	19	4	0	0	0	0
17	18	1	0	0	0	0
18	40	1	0	0	0	0
18	41	1	0	0	0	0
18	42	1	0	0	0	0
19	43	1	0	0	0	0
20	21	1	0	0	0	0
20	27	1	0	0	0	0

```
 20 35  1  0  0  0  0
 21 22  1  0  0  0  0
 21 23  1  0  0  0  0
 21 31  1  0  0  0  0
 22 28  1  0  0  0  0
 22 29  1  0  0  0  0
 22 30  1  0  0  0  0
 23 32  1  0  0  0  0
 23 33  1  0  0  0  0
 23 34  1  0  0  0  0
M  END
$$$$
4c


 40 41  0  0  0  0  0  0  0  0  0 V2000
    2.3567   -0.8467   -0.0002 C   0  0  0  0  0  0  0  0  0  0  0  0
    0.9455   -1.3651   -0.0005 C   0  0  0  0  0  0  0  0  0  0  0  0
    0.7555   -2.5662   -0.0011 O   0  0  0  0  0  0  0  0  0  0  0  0
   -0.1369   -0.3670   -0.0001 C   0  0  0  0  0  0  0  0  0  0  0  0
   -1.4081   -0.7922   -0.0007 C   0  0  0  0  0  0  0  0  0  0  0  0
   -2.6166    0.0231   -0.0006 C   0  0  0  0  0  0  0  0  0  0  0  0
    2.5679    0.4696    0.0003 N   0  0  0  0  0  0  0  0  0  0  0  0
    3.8293    0.8672    0.0005 C   0  0  0  0  0  0  0  0  0  0  0  0
    4.9028   -0.0326    0.0002 C   0  0  0  0  0  0  0  0  0  0  0  0
    4.6839   -1.3463   -0.0003 N   0  0  0  0  0  0  0  0  0  0  0  0
    3.4168   -1.7487   -0.0005 C   0  0  0  0  0  0  0  0  0  0  0  0
   -2.5980    1.4184   -0.0010 C   0  0  0  0  0  0  0  0  0  0  0  0
   -3.7801    2.1425   -0.0008 C   0  0  0  0  0  0  0  0  0  0  0  0
   -4.9989    1.4834   -0.0002 C   0  0  0  0  0  0  0  0  0  0  0  0
   -6.1535    2.1863   -0.0000 O   0  0  0  0  0  0  0  0  0  0  0  0
   -5.0331    0.0826    0.0002 C   0  0  0  0  0  0  0  0  0  0  0  0
   -6.2948   -0.4413    0.0008 O   0  0  0  0  0  0  0  0  0  0  0  0
   -6.4173   -1.8564    0.0016 C   0  0  0  0  0  0  0  0  0  0  0  0
```

```
 -3.8549   -0.6385   -0.0000 C   0  0  0  0  0  0  0  0  0  0  0  0
  6.3315    0.4485    0.0004 C   0  0  0  0  0  0  0  0  0  0  0  0
  6.6382    1.2603    1.2630 C   0  0  0  0  0  0  0  0  0  0  0  0
  6.6384    1.2608   -1.2619 C   0  0  0  0  0  0  0  0  0  0  0  0
  0.1457    0.6740    0.0007 H   0  0  0  0  0  0  0  0  0  0  0  0
 -1.5563   -1.8677   -0.0014 H   0  0  0  0  0  0  0  0  0  0  0  0
  4.0063    1.9356    0.0010 H   0  0  0  0  0  0  0  0  0  0  0  0
  6.4215    0.6851    2.1628 H   0  0  0  0  0  0  0  0  0  0  0  0
  6.0462    2.1768    1.2915 H   0  0  0  0  0  0  0  0  0  0  0  0
  7.6915    1.5420    1.2788 H   0  0  0  0  0  0  0  0  0  0  0  0
  6.4228    0.6857   -2.1619 H   0  0  0  0  0  0  0  0  0  0  0  0
  6.0457    2.1768   -1.2906 H   0  0  0  0  0  0  0  0  0  0  0  0
  7.6915    1.5434   -1.2767 H   0  0  0  0  0  0  0  0  0  0  0  0
  6.9430   -0.4537    0.0002 H   0  0  0  0  0  0  0  0  0  0  0  0
  3.2184   -2.8113   -0.0009 H   0  0  0  0  0  0  0  0  0  0  0  0
 -1.6562    1.9472   -0.0016 H   0  0  0  0  0  0  0  0  0  0  0  0
 -3.7806    3.2231   -0.0012 H   0  0  0  0  0  0  0  0  0  0  0  0
 -6.8835    1.5569    0.0005 H   0  0  0  0  0  0  0  0  0  0  0  0
 -7.4818   -2.0657    0.0021 H   0  0  0  0  0  0  0  0  0  0  0  0
 -5.9575   -2.2797    0.8955 H   0  0  0  0  0  0  0  0  0  0  0  0
 -5.9582   -2.2806   -0.8923 H   0  0  0  0  0  0  0  0  0  0  0  0
 -3.8696   -1.7189    0.0003 H   0  0  0  0  0  0  0  0  0  0  0  0
  1  2  1  0  0  0  0
  1  7  4  0  0  0  0
  1 11  4  0  0  0  0
  2  3  2  0  0  0  0
  2  4  1  0  0  0  0
  4  5  2  0  0  0  0
  4 23  1  0  0  0  0
  5  6  1  0  0  0  0
  5 24  1  0  0  0  0
  6 12  4  0  0  0  0
  6 19  4  0  0  0  0
  7  8  4  0  0  0  0
```

  8 9 4 0 0 0 0
  8 25 1 0 0 0 0
  9 10 4 0 0 0 0
  9 20 1 0 0 0 0
 10 11 4 0 0 0 0
 11 33 1 0 0 0 0
 12 13 4 0 0 0 0
 12 34 1 0 0 0 0
 13 14 4 0 0 0 0
 13 35 1 0 0 0 0
 14 15 1 0 0 0 0
 14 16 4 0 0 0 0
 15 36 1 0 0 0 0
 16 17 1 0 0 0 0
 16 19 4 0 0 0 0
 17 18 1 0 0 0 0
 18 37 1 0 0 0 0
 18 38 1 0 0 0 0
 18 39 1 0 0 0 0
 19 40 1 0 0 0 0
 20 21 1 0 0 0 0
 20 22 1 0 0 0 0
 20 32 1 0 0 0 0
 21 26 1 0 0 0 0
 21 27 1 0 0 0 0
 21 28 1 0 0 0 0
 22 29 1 0 0 0 0
 22 30 1 0 0 0 0
 22 31 1 0 0 0 0
M END
$$$$

4c_A

39 40 0 0 0 0 0 0 0 0 0 V2000

-2.3558	-0.8215	-0.0352	C	0	0	0	0	0	0	0	0	0	0	0	0
-0.9466	-1.3781	-0.0823	C	0	0	0	0	0	0	0	0	0	0	0	0
-0.8393	-2.6083	-0.1229	O	0	0	0	0	0	0	0	0	0	0	0	0
0.1217	-0.4313	-0.0756	C	0	0	0	0	0	0	0	0	0	0	0	0
1.4232	-0.8730	-0.1211	C	0	0	0	0	0	0	0	0	0	0	0	0
2.6153	-0.1182	-0.1216	C	0	0	0	0	0	0	0	0	0	0	0	0
-2.5704	0.4927	0.0122	N	0	0	0	0	0	0	0	0	0	0	0	0
-3.8336	0.8924	0.0511	C	0	0	0	0	0	0	0	0	0	0	0	0
-4.9091	0.0033	0.0441	C	0	0	0	0	0	0	0	0	0	0	0	0
-4.6959	-1.3122	-0.0035	N	0	0	0	0	0	0	0	0	0	0	0	0
-3.4298	-1.7152	-0.0426	C	0	0	0	0	0	0	0	0	0	0	0	0
2.6500	1.3004	-0.0709	C	0	0	0	0	0	0	0	0	0	0	0	0
3.8278	1.9877	-0.0741	C	0	0	0	0	0	0	0	0	0	0	0	0
5.1202	1.3359	-0.1422	C	0	0	0	0	0	0	0	0	0	0	0	0
6.1936	1.9678	-0.1755	O	0	0	0	0	0	0	0	0	0	0	0	0
5.0573	-0.1192	-0.1728	C	0	0	0	0	0	0	0	0	0	0	0	0
6.2273	-0.8442	-0.2732	O	0	0	0	0	0	0	0	0	0	0	0	0
7.0937	-0.6790	0.8433	C	0	0	0	0	0	0	0	0	0	0	0	0
3.8699	-0.7871	-0.1665	C	0	0	0	0	0	0	0	0	0	0	0	0
-6.3392	0.4862	0.0870	C	0	0	0	0	0	0	0	0	0	0	0	0
-6.6789	1.3237	-1.1497	C	0	0	0	0	0	0	0	0	0	0	0	0
-6.6245	1.2709	1.3711	C	0	0	0	0	0	0	0	0	0	0	0	0
-0.1336	0.6153	-0.0352	H	0	0	0	0	0	0	0	0	0	0	0	0
1.5401	-1.9527	-0.1617	H	0	0	0	0	0	0	0	0	0	0	0	0
-4.0036	1.9625	0.0894	H	0	0	0	0	0	0	0	0	0	0	0	0
-6.4856	0.7636	-2.0643	H	0	0	0	0	0	0	0	0	0	0	0	0
-6.0793	2.2354	-1.1769	H	0	0	0	0	0	0	0	0	0	0	0	0
-7.7311	1.6130	-1.1346	H	0	0	0	0	0	0	0	0	0	0	0	0
-6.3890	0.6748	2.2525	H	0	0	0	0	0	0	0	0	0	0	0	0
-6.0272	2.1836	1.4092	H	0	0	0	0	0	0	0	0	0	0	0	0
-7.6769	1.5559	1.4152	H	0	0	0	0	0	0	0	0	0	0	0	0
-6.9502	-0.4169	0.0811	H	0	0	0	0	0	0	0	0	0	0	0	0
-3.2278	-2.7758	-0.0817	H	0	0	0	0	0	0	0	0	0	0	0	0

1.7158	1.8466	-0.0310	H	0	0	0	0	0	0	0	0	0	0	0	0
3.8471	3.0698	-0.0454	H	0	0	0	0	0	0	0	0	0	0	0	0
7.9689	-1.2963	0.6488	H	0	0	0	0	0	0	0	0	0	0	0	0
6.5975	-1.0272	1.7545	H	0	0	0	0	0	0	0	0	0	0	0	0
7.3830	0.3646	0.9477	H	0	0	0	0	0	0	0	0	0	0	0	0
3.8948	-1.8703	-0.2084	H	0	0	0	0	0	0	0	0	0	0	0	0

1	2	1	0	0	0	0
1	7	4	0	0	0	0
1	11	4	0	0	0	0
2	3	2	0	0	0	0
2	4	1	0	0	0	0
4	5	2	0	0	0	0
4	23	1	0	0	0	0
5	6	1	0	0	0	0
5	24	1	0	0	0	0
6	12	4	0	0	0	0
6	19	4	0	0	0	0
7	8	4	0	0	0	0
8	9	4	0	0	0	0
8	25	1	0	0	0	0
9	10	4	0	0	0	0
9	20	1	0	0	0	0
10	11	4	0	0	0	0
11	33	1	0	0	0	0
12	13	4	0	0	0	0
12	34	1	0	0	0	0
13	14	4	0	0	0	0
13	35	1	0	0	0	0
14	15	1	0	0	0	0
14	16	4	0	0	0	0
16	17	1	0	0	0	0
16	19	4	0	0	0	0
17	18	1	0	0	0	0
18	36	1	0	0	0	0

```
 18 37  1  0  0  0  0
 18 38  1  0  0  0  0
 19 39  1  0  0  0  0
 20 21  1  0  0  0  0
 20 22  1  0  0  0  0
 20 32  1  0  0  0  0
 21 26  1  0  0  0  0
 21 27  1  0  0  0  0
 21 28  1  0  0  0  0
 22 29  1  0  0  0  0
 22 30  1  0  0  0  0
 22 31  1  0  0  0  0
M  END
$$$$
4c_NR


 39 40  0  0  0  0  0  0  0  0  0 V2000
   -2.3878   -0.8883   -0.0005 C   0  0  0  0  0  0  0  0  0  0  0  0
   -0.9988   -1.4558   -0.0006 C   0  0  0  0  0  0  0  0  0  0  0  0
   -0.8406   -2.6603   -0.0006 O   0  0  0  0  0  0  0  0  0  0  0  0
    0.1219   -0.4960   -0.0005 C   0  0  0  0  0  0  0  0  0  0  0  0
    1.3830   -0.9664   -0.0004 C   0  0  0  0  0  0  0  0  0  0  0  0
    2.5940   -0.1783   -0.0002 C   0  0  0  0  0  0  0  0  0  0  0  0
   -2.5469    0.4358   -0.0006 N   0  0  0  0  0  0  0  0  0  0  0  0
   -3.7913    0.8822   -0.0006 C   0  0  0  0  0  0  0  0  0  0  0  0
   -4.8995    0.0236   -0.0004 C   0  0  0  0  0  0  0  0  0  0  0  0
   -4.7313   -1.2973   -0.0003 N   0  0  0  0  0  0  0  0  0  0  0  0
   -3.4809   -1.7492   -0.0004 C   0  0  0  0  0  0  0  0  0  0  0  0
    2.5762    1.2532   -0.0004 C   0  0  0  0  0  0  0  0  0  0  0  0
    3.7322    1.9548   -0.0003 C   0  0  0  0  0  0  0  0  0  0  0  0
    5.0349    1.3097    0.0001 C   0  0  0  0  0  0  0  0  0  0  0  0
    6.0813    1.9690    0.0003 O   0  0  0  0  0  0  0  0  0  0  0  0
    5.0247   -0.1584    0.0003 C   0  0  0  0  0  0  0  0  0  0  0  0
```

6.1231	-0.9104	0.0006	O	0	0	0	0	0	0	0	0	0	0	0	0
7.4558	-0.3689	0.0007	C	0	0	0	0	0	0	0	0	0	0	0	0
3.8187	-0.8425	0.0001	C	0	0	0	0	0	0	0	0	0	0	0	0
-6.3083	0.5589	0.0000	C	0	0	0	0	0	0	0	0	0	0	0	0
-6.5842	1.3836	-1.2615	C	0	0	0	0	0	0	0	0	0	0	0	0
-6.5843	1.3797	1.2641	C	0	0	0	0	0	0	0	0	0	0	0	0
-0.1246	0.5536	-0.0006	H	0	0	0	0	0	0	0	0	0	0	0	0
1.5045	-2.0444	-0.0003	H	0	0	0	0	0	0	0	0	0	0	0	0
-3.9266	1.9565	-0.0007	H	0	0	0	0	0	0	0	0	0	0	0	0
-6.3918	0.8013	-2.1622	H	0	0	0	0	0	0	0	0	0	0	0	0
-5.9579	2.2769	-1.2902	H	0	0	0	0	0	0	0	0	0	0	0	0
-7.6257	1.7055	-1.2750	H	0	0	0	0	0	0	0	0	0	0	0	0
-6.3924	0.7944	2.1629	H	0	0	0	0	0	0	0	0	0	0	0	0
-5.9574	2.2726	1.2957	H	0	0	0	0	0	0	0	0	0	0	0	0
-7.6256	1.7021	1.2783	H	0	0	0	0	0	0	0	0	0	0	0	0
-6.9537	-0.3192	-0.0013	H	0	0	0	0	0	0	0	0	0	0	0	0
-3.3248	-2.8189	-0.0003	H	0	0	0	0	0	0	0	0	0	0	0	0
1.6301	1.7744	-0.0007	H	0	0	0	0	0	0	0	0	0	0	0	0
3.7499	3.0356	-0.0004	H	0	0	0	0	0	0	0	0	0	0	0	0
8.0956	-1.2452	0.0009	H	0	0	0	0	0	0	0	0	0	0	0	0
7.6228	0.2351	0.8870	H	0	0	0	0	0	0	0	0	0	0	0	0
7.6231	0.2349	-0.8857	H	0	0	0	0	0	0	0	0	0	0	0	0
3.8527	-1.9237	0.0002	H	0	0	0	0	0	0	0	0	0	0	0	0

1	2	1	0	0	0	0
1	7	4	0	0	0	0
1	11	4	0	0	0	0
2	3	2	0	0	0	0
2	4	1	0	0	0	0
4	5	2	0	0	0	0
4	23	1	0	0	0	0
5	6	1	0	0	0	0
5	24	1	0	0	0	0
6	12	4	0	0	0	0
6	19	4	0	0	0	0

```
  7  8  4  0  0  0  0
  8  9  4  0  0  0  0
  8 25  1  0  0  0  0
  9 10  4  0  0  0  0
  9 20  1  0  0  0  0
 10 11  4  0  0  0  0
 11 33  1  0  0  0  0
 12 13  4  0  0  0  0
 12 34  1  0  0  0  0
 13 14  4  0  0  0  0
 13 35  1  0  0  0  0
 14 15  1  0  0  0  0
 14 16  4  0  0  0  0
 16 17  1  0  0  0  0
 16 19  4  0  0  0  0
 17 18  1  0  0  0  0
 18 36  1  0  0  0  0
 18 37  1  0  0  0  0
 18 38  1  0  0  0  0
 19 39  1  0  0  0  0
 20 21  1  0  0  0  0
 20 22  1  0  0  0  0
 20 32  1  0  0  0  0
 21 26  1  0  0  0  0
 21 27  1  0  0  0  0
 21 28  1  0  0  0  0
 22 29  1  0  0  0  0
 22 30  1  0  0  0  0
 22 31  1  0  0  0  0
M  END
$$$$
4c_PR
```

```
40 41  0  0  0  0  0  0  0  0  0 V2000
   2.3492   -0.8986    0.0002 C   0  0  0  0  0  0  0  0  0  0  0  0
   0.9589   -1.4149    0.0002 C   0  0  0  0  0  0  0  0  0  0  0  0
   0.6994   -2.5966    0.0002 O   0  0  0  0  0  0  0  0  0  0  0  0
  -0.1420   -0.3973    0.0003 C   0  0  0  0  0  0  0  0  0  0  0  0
  -1.4230   -0.8454   -0.0001 C   0  0  0  0  0  0  0  0  0  0  0  0
  -2.5946   -0.0318   -0.0001 C   0  0  0  0  0  0  0  0  0  0  0  0
   2.5269    0.4272    0.0000 N   0  0  0  0  0  0  0  0  0  0  0  0
   3.7753    0.8515   -0.0000 C   0  0  0  0  0  0  0  0  0  0  0  0
   4.8714   -0.0333   -0.0000 C   0  0  0  0  0  0  0  0  0  0  0  0
   4.6783   -1.3498    0.0001 N   0  0  0  0  0  0  0  0  0  0  0  0
   3.4216   -1.7827    0.0002 C   0  0  0  0  0  0  0  0  0  0  0  0
  -2.5298    1.4015   -0.0000 C   0  0  0  0  0  0  0  0  0  0  0  0
  -3.6617    2.1567    0.0001 C   0  0  0  0  0  0  0  0  0  0  0  0
  -4.9126    1.5109    0.0001 C   0  0  0  0  0  0  0  0  0  0  0  0
  -6.0064    2.2222    0.0002 O   0  0  0  0  0  0  0  0  0  0  0  0
  -5.0002    0.0728   -0.0000 C   0  0  0  0  0  0  0  0  0  0  0  0
  -6.2549   -0.3624   -0.0000 O   0  0  0  0  0  0  0  0  0  0  0  0
  -6.5002   -1.7835   -0.0003 C   0  0  0  0  0  0  0  0  0  0  0  0
  -3.8515   -0.6753   -0.0002 C   0  0  0  0  0  0  0  0  0  0  0  0
   6.2851    0.4790   -0.0001 C   0  0  0  0  0  0  0  0  0  0  0  0
   6.5685    1.2987    1.2641 C   0  0  0  0  0  0  0  0  0  0  0  0
   6.5684    1.2984   -1.2645 C   0  0  0  0  0  0  0  0  0  0  0  0
   0.1453    0.6429    0.0008 H   0  0  0  0  0  0  0  0  0  0  0  0
  -1.5593   -1.9215   -0.0005 H   0  0  0  0  0  0  0  0  0  0  0  0
   3.9343    1.9218   -0.0001 H   0  0  0  0  0  0  0  0  0  0  0  0
   6.3667    0.7207    2.1652 H   0  0  0  0  0  0  0  0  0  0  0  0
   5.9612    2.2052    1.2907 H   0  0  0  0  0  0  0  0  0  0  0  0
   7.6154    1.6003    1.2779 H   0  0  0  0  0  0  0  0  0  0  0  0
   6.3665    0.7202   -2.1655 H   0  0  0  0  0  0  0  0  0  0  0  0
   5.9611    2.2049   -1.2912 H   0  0  0  0  0  0  0  0  0  0  0  0
   7.6153    1.6000   -1.2785 H   0  0  0  0  0  0  0  0  0  0  0  0
   6.9182   -0.4076   -0.0000 H   0  0  0  0  0  0  0  0  0  0  0  0
   3.2512   -2.8509    0.0003 H   0  0  0  0  0  0  0  0  0  0  0  0
```

```
 -1.5678    1.8900   -0.0000 H   0  0  0  0  0  0  0  0  0  0  0  0
 -3.6418    3.2367    0.0001 H   0  0  0  0  0  0  0  0  0  0  0  0
 -6.7889    1.6466    0.0002 H   0  0  0  0  0  0  0  0  0  0  0  0
 -7.5776   -1.8910   -0.0004 H   0  0  0  0  0  0  0  0  0  0  0  0
 -6.0745   -2.2253    0.8986 H   0  0  0  0  0  0  0  0  0  0  0  0
 -6.0743   -2.2249   -0.8993 H   0  0  0  0  0  0  0  0  0  0  0  0
 -3.8884   -1.7543   -0.0002 H   0  0  0  0  0  0  0  0  0  0  0  0
  1  2  1  0  0  0  0
  1  7  4  0  0  0  0
  1 11  4  0  0  0  0
  2  3  2  0  0  0  0
  2  4  1  0  0  0  0
  4  5  2  0  0  0  0
  4 23  1  0  0  0  0
  5  6  1  0  0  0  0
  5 24  1  0  0  0  0
  6 12  4  0  0  0  0
  6 19  4  0  0  0  0
  7  8  4  0  0  0  0
  8  9  4  0  0  0  0
  8 25  1  0  0  0  0
  9 10  4  0  0  0  0
  9 20  1  0  0  0  0
 10 11  4  0  0  0  0
 11 33  1  0  0  0  0
 12 13  4  0  0  0  0
 12 34  1  0  0  0  0
 13 14  4  0  0  0  0
 13 35  1  0  0  0  0
 14 15  1  0  0  0  0
 14 16  4  0  0  0  0
 15 36  1  0  0  0  0
 16 17  1  0  0  0  0
 16 19  4  0  0  0  0
```

```
 17 18  1  0  0  0  0
 18 37  1  0  0  0  0
 18 38  1  0  0  0  0
 18 39  1  0  0  0  0
 19 40  1  0  0  0  0
 20 21  1  0  0  0  0
 20 22  1  0  0  0  0
 20 32  1  0  0  0  0
 21 26  1  0  0  0  0
 21 27  1  0  0  0  0
 21 28  1  0  0  0  0
 22 29  1  0  0  0  0
 22 30  1  0  0  0  0
 22 31  1  0  0  0  0
M  END
$$$$
1


 29 30  0  0  0  0  0  0  0  0  0 V2000
   -2.6381   -0.2951    0.0150 C   0  0  0  0  0  0  0  0  0  0  0  0
   -1.3378   -1.0399    0.0020 C   0  0  0  0  0  0  0  0  0  0  0  0
   -1.3365   -2.2553    0.0266 O   0  0  0  0  0  0  0  0  0  0  0  0
   -0.0796   -0.2551   -0.0584 C   0  0  0  0  0  0  0  0  0  0  0  0
    1.0851   -0.9050    0.0704 C   0  0  0  0  0  0  0  0  0  0  0  0
    2.4498   -0.3897    0.0348 C   0  0  0  0  0  0  0  0  0  0  0  0
   -2.7320    1.0588    0.3355 C   0  0  0  0  0  0  0  0  0  0  0  0
   -3.9713    1.6870    0.3558 C   0  0  0  0  0  0  0  0  0  0  0  0
   -5.1199    0.9709    0.0443 C   0  0  0  0  0  0  0  0  0  0  0  0
   -5.0320   -0.3810   -0.2753 C   0  0  0  0  0  0  0  0  0  0  0  0
   -3.7983   -1.0117   -0.2820 C   0  0  0  0  0  0  0  0  0  0  0  0
    3.4950   -1.3116    0.1768 C   0  0  0  0  0  0  0  0  0  0  0  0
    4.8265   -0.9325    0.1578 C   0  0  0  0  0  0  0  0  0  0  0  0
    5.1447    0.4103   -0.0047 C   0  0  0  0  0  0  0  0  0  0  0  0
```

4.1344	1.3501	-0.1463	C	0	0	0	0	0	0	0	0	0	0	0	0
2.8004	0.9601	-0.1283	C	0	0	0	0	0	0	0	0	0	0	0	0
1.8069	1.8807	-0.2663	O	0	0	0	0	0	0	0	0	0	0	0	0
-1.8487	1.6247	0.5941	H	0	0	0	0	0	0	0	0	0	0	0	0
-5.9262	-0.9396	-0.5156	H	0	0	0	0	0	0	0	0	0	0	0	0
2.1914	2.7530	-0.3771	H	0	0	0	0	0	0	0	0	0	0	0	0
-0.1218	0.8057	-0.2258	H	0	0	0	0	0	0	0	0	0	0	0	0
1.0112	-1.9774	0.2219	H	0	0	0	0	0	0	0	0	0	0	0	0
-4.0395	2.7343	0.6158	H	0	0	0	0	0	0	0	0	0	0	0	0
-6.0826	1.4635	0.0538	H	0	0	0	0	0	0	0	0	0	0	0	0
-3.7052	-2.0631	-0.5140	H	0	0	0	0	0	0	0	0	0	0	0	0
3.2358	-2.3544	0.3043	H	0	0	0	0	0	0	0	0	0	0	0	0
5.6057	-1.6722	0.2695	H	0	0	0	0	0	0	0	0	0	0	0	0
6.1769	0.7313	-0.0216	H	0	0	0	0	0	0	0	0	0	0	0	0
4.3783	2.3982	-0.2729	H	0	0	0	0	0	0	0	0	0	0	0	0

1	2	1	0	0	0	0
1	7	4	0	0	0	0
1	11	4	0	0	0	0
2	3	2	0	0	0	0
2	4	1	0	0	0	0
4	5	2	0	0	0	0
4	21	1	0	0	0	0
5	6	1	0	0	0	0
5	22	1	0	0	0	0
6	12	4	0	0	0	0
6	16	4	0	0	0	0
7	8	4	0	0	0	0
7	18	1	0	0	0	0
8	9	4	0	0	0	0
8	23	1	0	0	0	0
9	10	4	0	0	0	0
9	24	1	0	0	0	0
10	11	4	0	0	0	0
10	19	1	0	0	0	0

```
 11 25  1  0  0  0  0
 12 13  4  0  0  0  0
 12 26  1  0  0  0  0
 13 14  4  0  0  0  0
 13 27  1  0  0  0  0
 14 15  4  0  0  0  0
 14 28  1  0  0  0  0
 15 16  4  0  0  0  0
 15 29  1  0  0  0  0
 16 17  1  0  0  0  0
 17 20  1  0  0  0  0
M  END
$$$$
1_A


 28 29  0  0  0  0  0  0  0  0  0 V2000
   -2.5893   -0.2885    0.0193 C   0  0  0  0  0  0  0  0  0  0  0  0
   -1.2862   -1.0607    0.0299 C   0  0  0  0  0  0  0  0  0  0  0  0
   -1.3526   -2.2883    0.0821 O   0  0  0  0  0  0  0  0  0  0  0  0
   -0.0578   -0.2997   -0.0315 C   0  0  0  0  0  0  0  0  0  0  0  0
    1.1409   -0.9442    0.0430 C   0  0  0  0  0  0  0  0  0  0  0  0
    2.4539   -0.3888    0.0102 C   0  0  0  0  0  0  0  0  0  0  0  0
   -2.6686    1.0844    0.2532 C   0  0  0  0  0  0  0  0  0  0  0  0
   -3.9016    1.7276    0.2493 C   0  0  0  0  0  0  0  0  0  0  0  0
   -5.0665    1.0100    0.0052 C   0  0  0  0  0  0  0  0  0  0  0  0
   -4.9947   -0.3603   -0.2281 C   0  0  0  0  0  0  0  0  0  0  0  0
   -3.7648   -1.0024   -0.2148 C   0  0  0  0  0  0  0  0  0  0  0  0
    3.5445   -1.2817    0.1020 C   0  0  0  0  0  0  0  0  0  0  0  0
    4.8548   -0.8666    0.0888 C   0  0  0  0  0  0  0  0  0  0  0  0
    5.1113    0.5198   -0.0186 C   0  0  0  0  0  0  0  0  0  0  0  0
    4.0932    1.4288   -0.1073 C   0  0  0  0  0  0  0  0  0  0  0  0
    2.6969    1.0444   -0.1011 C   0  0  0  0  0  0  0  0  0  0  0  0
    1.7889    1.9026   -0.1837 O   0  0  0  0  0  0  0  0  0  0  0  0
```

-1.7716	1.6526	0.4513	H	0	0	0	0	0	0	0	0	0	0	0	0
-5.8983	-0.9249	-0.4181	H	0	0	0	0	0	0	0	0	0	0	0	0
-0.0647	0.7691	-0.1483	H	0	0	0	0	0	0	0	0	0	0	0	0
1.0865	-2.0257	0.1467	H	0	0	0	0	0	0	0	0	0	0	0	0
-3.9497	2.7920	0.4377	H	0	0	0	0	0	0	0	0	0	0	0	0
-6.0239	1.5144	-0.0012	H	0	0	0	0	0	0	0	0	0	0	0	0
-3.6799	-2.0674	-0.3794	H	0	0	0	0	0	0	0	0	0	0	0	0
3.3183	-2.3399	0.1860	H	0	0	0	0	0	0	0	0	0	0	0	0
5.6672	-1.5765	0.1597	H	0	0	0	0	0	0	0	0	0	0	0	0
6.1380	0.8702	-0.0299	H	0	0	0	0	0	0	0	0	0	0	0	0
4.2921	2.4899	-0.1890	H	0	0	0	0	0	0	0	0	0	0	0	0

1	2	1	0	0	0	0
1	7	4	0	0	0	0
1	11	4	0	0	0	0
2	3	2	0	0	0	0
2	4	1	0	0	0	0
4	5	2	0	0	0	0
4	20	1	0	0	0	0
5	6	1	0	0	0	0
5	21	1	0	0	0	0
6	12	4	0	0	0	0
6	16	4	0	0	0	0
7	8	4	0	0	0	0
7	18	1	0	0	0	0
8	9	4	0	0	0	0
8	22	1	0	0	0	0
9	10	4	0	0	0	0
9	23	1	0	0	0	0
10	11	4	0	0	0	0
10	19	1	0	0	0	0
11	24	1	0	0	0	0
12	13	4	0	0	0	0
12	25	1	0	0	0	0
13	14	4	0	0	0	0

```
 13 26  1  0  0  0  0
 14 15  4  0  0  0  0
 14 27  1  0  0  0  0
 15 16  4  0  0  0  0
 15 28  1  0  0  0  0
 16 17  1  0  0  0  0
M  END
$$$$
1_NR


 28 29  0  0  0  0  0  0  0  0  0 V2000
   -2.6144   -0.2842    0.0064 C   0  0  0  0  0  0  0  0  0  0  0  0
   -1.3259   -1.0423   -0.0058 C   0  0  0  0  0  0  0  0  0  0  0  0
   -1.3266   -2.2575    0.0202 O   0  0  0  0  0  0  0  0  0  0  0  0
   -0.0530   -0.2755   -0.0644 C   0  0  0  0  0  0  0  0  0  0  0  0
    1.1082   -0.9439    0.0642 C   0  0  0  0  0  0  0  0  0  0  0  0
    2.4475   -0.4084    0.0326 C   0  0  0  0  0  0  0  0  0  0  0  0
   -2.6835    1.0878    0.2494 C   0  0  0  0  0  0  0  0  0  0  0  0
   -3.9157    1.7297    0.2707 C   0  0  0  0  0  0  0  0  0  0  0  0
   -5.0801    1.0090    0.0393 C   0  0  0  0  0  0  0  0  0  0  0  0
   -5.0165   -0.3604   -0.2040 C   0  0  0  0  0  0  0  0  0  0  0  0
   -3.7902   -1.0042   -0.2140 C   0  0  0  0  0  0  0  0  0  0  0  0
    3.5155   -1.2949    0.1497 C   0  0  0  0  0  0  0  0  0  0  0  0
    4.8343   -0.8635    0.1260 C   0  0  0  0  0  0  0  0  0  0  0  0
    5.1450    0.5099   -0.0170 C   0  0  0  0  0  0  0  0  0  0  0  0
    4.1440    1.4235   -0.1319 C   0  0  0  0  0  0  0  0  0  0  0  0
    2.7430    1.0278   -0.1116 C   0  0  0  0  0  0  0  0  0  0  0  0
    1.8587    1.8849   -0.2086 O   0  0  0  0  0  0  0  0  0  0  0  0
   -1.7890    1.6619    0.4414 H   0  0  0  0  0  0  0  0  0  0  0  0
   -5.9233   -0.9209   -0.3835 H   0  0  0  0  0  0  0  0  0  0  0  0
   -0.0611    0.7877   -0.2239 H   0  0  0  0  0  0  0  0  0  0  0  0
    1.0345   -2.0175    0.2066 H   0  0  0  0  0  0  0  0  0  0  0  0
   -3.9644    2.7916    0.4673 H   0  0  0  0  0  0  0  0  0  0  0  0
```

```
 -6.0371    1.5125    0.0502 H   0  0  0  0  0  0  0  0  0  0  0  0
 -3.7150   -2.0676   -0.3910 H   0  0  0  0  0  0  0  0  0  0  0  0
  3.3026   -2.3501    0.2597 H   0  0  0  0  0  0  0  0  0  0  0  0
  5.6336   -1.5848    0.2177 H   0  0  0  0  0  0  0  0  0  0  0  0
  6.1794    0.8243   -0.0333 H   0  0  0  0  0  0  0  0  0  0  0  0
  4.3335    2.4817   -0.2415 H   0  0  0  0  0  0  0  0  0  0  0  0
 1  2  1  0  0  0  0
 1  7  4  0  0  0  0
 1 11  4  0  0  0  0
 2  3  2  0  0  0  0
 2  4  1  0  0  0  0
 4  5  2  0  0  0  0
 4 20  1  0  0  0  0
 5  6  1  0  0  0  0
 5 21  1  0  0  0  0
 6 12  4  0  0  0  0
 6 16  4  0  0  0  0
 7  8  4  0  0  0  0
 7 18  1  0  0  0  0
 8  9  4  0  0  0  0
 8 22  1  0  0  0  0
 9 10  4  0  0  0  0
 9 23  1  0  0  0  0
10 11  4  0  0  0  0
10 19  1  0  0  0  0
11 24  1  0  0  0  0
12 13  4  0  0  0  0
12 25  1  0  0  0  0
13 14  4  0  0  0  0
13 26  1  0  0  0  0
14 15  4  0  0  0  0
14 27  1  0  0  0  0
15 16  4  0  0  0  0
15 28  1  0  0  0  0
```

```
 16 17  1  0  0  0  0
M  END
$$$$
1_PR


 29 30  0  0  0  0  0  0  0  0  0 V2000
   -2.6431   -0.2867   -0.0237 C   0  0  0  0  0  0  0  0  0  0  0  0
   -1.3544   -0.9914   -0.1274 C   0  0  0  0  0  0  0  0  0  0  0  0
   -1.2525   -2.1969   -0.2233 O   0  0  0  0  0  0  0  0  0  0  0  0
   -0.0815   -0.1922   -0.1653 C   0  0  0  0  0  0  0  0  0  0  0  0
    1.0995   -0.8751    0.0506 C   0  0  0  0  0  0  0  0  0  0  0  0
    2.4206   -0.3912    0.0584 C   0  0  0  0  0  0  0  0  0  0  0  0
   -2.7299    1.0699    0.3018 C   0  0  0  0  0  0  0  0  0  0  0  0
   -3.9726    1.6751    0.4140 C   0  0  0  0  0  0  0  0  0  0  0  0
   -5.1260    0.9324    0.1905 C   0  0  0  0  0  0  0  0  0  0  0  0
   -5.0440   -0.4200   -0.1331 C   0  0  0  0  0  0  0  0  0  0  0  0
   -3.8076   -1.0318   -0.2337 C   0  0  0  0  0  0  0  0  0  0  0  0
    3.4595   -1.3377    0.3147 C   0  0  0  0  0  0  0  0  0  0  0  0
    4.7699   -0.9703    0.3281 C   0  0  0  0  0  0  0  0  0  0  0  0
    5.1156    0.3829    0.0876 C   0  0  0  0  0  0  0  0  0  0  0  0
    4.1563    1.3399   -0.1628 C   0  0  0  0  0  0  0  0  0  0  0  0
    2.8121    0.9791   -0.1779 C   0  0  0  0  0  0  0  0  0  0  0  0
    1.8522    1.8645   -0.4044 O   0  0  0  0  0  0  0  0  0  0  0  0
   -1.8437    1.6592    0.4932 H   0  0  0  0  0  0  0  0  0  0  0  0
   -5.9454   -0.9909   -0.3032 H   0  0  0  0  0  0  0  0  0  0  0  0
    2.2082    2.7462   -0.5687 H   0  0  0  0  0  0  0  0  0  0  0  0
   -0.1013    0.8550   -0.4099 H   0  0  0  0  0  0  0  0  0  0  0  0
    0.9816   -1.9370    0.2389 H   0  0  0  0  0  0  0  0  0  0  0  0
   -4.0433    2.7211    0.6751 H   0  0  0  0  0  0  0  0  0  0  0  0
   -6.0937    1.4079    0.2716 H   0  0  0  0  0  0  0  0  0  0  0  0
   -3.7192   -2.0811   -0.4761 H   0  0  0  0  0  0  0  0  0  0  0  0
    3.1728   -2.3646    0.4935 H   0  0  0  0  0  0  0  0  0  0  0  0
    5.5463   -1.6962    0.5183 H   0  0  0  0  0  0  0  0  0  0  0  0
```

```
    6.1574    0.6732    0.1005 H   0  0  0  0  0  0  0  0  0  0  0  0
    4.4379    2.3691   -0.3428 H   0  0  0  0  0  0  0  0  0  0  0  0
  1  2  1  0  0  0  0
  1  7  4  0  0  0  0
  1 11  4  0  0  0  0
  2  3  2  0  0  0  0
  2  4  1  0  0  0  0
  4  5  2  0  0  0  0
  4 21  1  0  0  0  0
  5  6  1  0  0  0  0
  5 22  1  0  0  0  0
  6 12  4  0  0  0  0
  6 16  4  0  0  0  0
  7  8  4  0  0  0  0
  7 18  1  0  0  0  0
  8  9  4  0  0  0  0
  8 23  1  0  0  0  0
  9 10  4  0  0  0  0
  9 24  1  0  0  0  0
 10 11  4  0  0  0  0
 10 19  1  0  0  0  0
 11 25  1  0  0  0  0
 12 13  4  0  0  0  0
 12 26  1  0  0  0  0
 13 14  4  0  0  0  0
 13 27  1  0  0  0  0
 14 15  4  0  0  0  0
 14 28  1  0  0  0  0
 15 16  4  0  0  0  0
 15 29  1  0  0  0  0
 16 17  1  0  0  0  0
 17 20  1  0  0  0  0
M  END
$$$$
```

1a

```
 27 28  0  0  0  0  0  0  0  0  0 V2000
   -2.6369   -0.2664    0.0080 C   0  0  0  0  0  0  0  0  0  0  0  0
   -1.3558   -1.0264    0.2194 C   0  0  0  0  0  0  0  0  0  0  0  0
   -1.4021   -2.2039    0.5080 O   0  0  0  0  0  0  0  0  0  0  0  0
   -0.0951   -0.2751    0.0547 C   0  0  0  0  0  0  0  0  0  0  0  0
    1.0683   -0.9353    0.1824 C   0  0  0  0  0  0  0  0  0  0  0  0
    2.4221   -0.4124    0.0364 C   0  0  0  0  0  0  0  0  0  0  0  0
   -2.5746    1.0416   -0.2252 N   0  0  0  0  0  0  0  0  0  0  0  0
   -3.7324    1.6665   -0.3969 C   0  0  0  0  0  0  0  0  0  0  0  0
   -4.9447    0.9854   -0.3395 C   0  0  0  0  0  0  0  0  0  0  0  0
   -5.0124   -0.3222   -0.1090 N   0  0  0  0  0  0  0  0  0  0  0  0
   -3.8540   -0.9465    0.0670 C   0  0  0  0  0  0  0  0  0  0  0  0
    3.4308   -1.2976   -0.3702 C   0  0  0  0  0  0  0  0  0  0  0  0
    4.7276   -0.8714   -0.5849 C   0  0  0  0  0  0  0  0  0  0  0  0
    5.0472    0.4665   -0.3605 C   0  0  0  0  0  0  0  0  0  0  0  0
    4.0838    1.3535    0.0878 C   0  0  0  0  0  0  0  0  0  0  0  0
    2.7773    0.9224    0.2935 C   0  0  0  0  0  0  0  0  0  0  0  0
    1.8999    1.8403    0.7696 O   0  0  0  0  0  0  0  0  0  0  0  0
   -0.1743    0.7596   -0.2433 H   0  0  0  0  0  0  0  0  0  0  0  0
    0.9929   -1.9995    0.3857 H   0  0  0  0  0  0  0  0  0  0  0  0
   -3.7005    2.7316   -0.5855 H   0  0  0  0  0  0  0  0  0  0  0  0
   -5.8801    1.5104   -0.4843 H   0  0  0  0  0  0  0  0  0  0  0  0
   -3.8676   -2.0100    0.2599 H   0  0  0  0  0  0  0  0  0  0  0  0
    3.1653   -2.3336   -0.5368 H   0  0  0  0  0  0  0  0  0  0  0  0
    5.4834   -1.5681   -0.9167 H   0  0  0  0  0  0  0  0  0  0  0  0
    6.0577    0.8165   -0.5201 H   0  0  0  0  0  0  0  0  0  0  0  0
    4.3183    2.3871    0.2984 H   0  0  0  0  0  0  0  0  0  0  0  0
    1.1019    1.4001    1.0778 H   0  0  0  0  0  0  0  0  0  0  0  0
  1  2  1  0  0  0  0
  1  7  4  0  0  0  0
  1 11  4  0  0  0  0
```

```
  2  3  2  0  0  0  0
  2  4  1  0  0  0  0
  4  5  2  0  0  0  0
  4 18  1  0  0  0  0
  5  6  1  0  0  0  0
  5 19  1  0  0  0  0
  6 12  4  0  0  0  0
  6 16  4  0  0  0  0
  7  8  4  0  0  0  0
  8  9  4  0  0  0  0
  8 20  1  0  0  0  0
  9 10  4  0  0  0  0
  9 21  1  0  0  0  0
 10 11  4  0  0  0  0
 11 22  1  0  0  0  0
 12 13  4  0  0  0  0
 12 23  1  0  0  0  0
 13 14  4  0  0  0  0
 13 24  1  0  0  0  0
 14 15  4  0  0  0  0
 14 25  1  0  0  0  0
 15 16  4  0  0  0  0
 15 26  1  0  0  0  0
 16 17  1  0  0  0  0
 17 27  1  0  0  0  0
M  END
$$$$
1a_A


 26 27  0  0  0  0  0  0  0  0  0 V2000
   -2.6116   -0.2479   -0.0001 C   0  0  0  0  0  0  0  0  0  0  0  0
   -1.3003   -1.0130   -0.0003 C   0  0  0  0  0  0  0  0  0  0  0  0
   -1.3873   -2.2435   -0.0004 O   0  0  0  0  0  0  0  0  0  0  0  0
```

-0.0924	-0.2370	-0.0003	C	0	0	0	0	0	0	0	0	0	0	0	0
1.1068	-0.8976	-0.0002	C	0	0	0	0	0	0	0	0	0	0	0	0
2.4277	-0.3767	-0.0001	C	0	0	0	0	0	0	0	0	0	0	0	0
-2.6177	1.0811	-0.0006	N	0	0	0	0	0	0	0	0	0	0	0	0
-3.8077	1.6712	-0.0004	C	0	0	0	0	0	0	0	0	0	0	0	0
-4.9911	0.9464	0.0003	C	0	0	0	0	0	0	0	0	0	0	0	0
-5.0006	-0.3862	0.0008	N	0	0	0	0	0	0	0	0	0	0	0	0
-3.8104	-0.9740	0.0006	C	0	0	0	0	0	0	0	0	0	0	0	0
3.4968	-1.3047	-0.0001	C	0	0	0	0	0	0	0	0	0	0	0	0
4.8154	-0.9257	0.0001	C	0	0	0	0	0	0	0	0	0	0	0	0
5.1116	0.4602	0.0003	C	0	0	0	0	0	0	0	0	0	0	0	0
4.1219	1.4009	0.0003	C	0	0	0	0	0	0	0	0	0	0	0	0
2.7117	1.0557	0.0001	C	0	0	0	0	0	0	0	0	0	0	0	0
1.8313	1.9389	-0.0000	O	0	0	0	0	0	0	0	0	0	0	0	0
-0.1388	0.8378	-0.0002	H	0	0	0	0	0	0	0	0	0	0	0	0
1.0338	-1.9831	-0.0003	H	0	0	0	0	0	0	0	0	0	0	0	0
-3.8203	2.7545	-0.0008	H	0	0	0	0	0	0	0	0	0	0	0	0
-5.9503	1.4495	0.0005	H	0	0	0	0	0	0	0	0	0	0	0	0
-3.7669	-2.0534	0.0010	H	0	0	0	0	0	0	0	0	0	0	0	0
3.2419	-2.3597	-0.0002	H	0	0	0	0	0	0	0	0	0	0	0	0
5.6088	-1.6600	0.0001	H	0	0	0	0	0	0	0	0	0	0	0	0
6.1485	0.7800	0.0005	H	0	0	0	0	0	0	0	0	0	0	0	0
4.3492	2.4595	0.0005	H	0	0	0	0	0	0	0	0	0	0	0	0

1	2	1	0	0	0	0
1	7	4	0	0	0	0
1	11	4	0	0	0	0
2	3	2	0	0	0	0
2	4	1	0	0	0	0
4	5	2	0	0	0	0
4	18	1	0	0	0	0
5	6	1	0	0	0	0
5	19	1	0	0	0	0
6	12	4	0	0	0	0
6	16	4	0	0	0	0

```
  7  8  4  0  0  0  0
  8  9  4  0  0  0  0
  8 20  1  0  0  0  0
  9 10  4  0  0  0  0
  9 21  1  0  0  0  0
 10 11  4  0  0  0  0
 11 22  1  0  0  0  0
 12 13  4  0  0  0  0
 12 23  1  0  0  0  0
 13 14  4  0  0  0  0
 13 24  1  0  0  0  0
 14 15  4  0  0  0  0
 14 25  1  0  0  0  0
 15 16  4  0  0  0  0
 15 26  1  0  0  0  0
 16 17  1  0  0  0  0
M  END
$$$$
1a_NR


 26 27  0  0  0  0  0  0  0  0  0 V2000
   -2.6350   -0.2679   -0.0001 C   0  0  0  0  0  0  0  0  0  0  0  0
   -1.3429   -1.0366   -0.0005 C   0  0  0  0  0  0  0  0  0  0  0  0
   -1.3725   -2.2505   -0.0007 O   0  0  0  0  0  0  0  0  0  0  0  0
   -0.0872   -0.2543   -0.0004 C   0  0  0  0  0  0  0  0  0  0  0  0
    1.0760   -0.9340   -0.0000 C   0  0  0  0  0  0  0  0  0  0  0  0
    2.4189   -0.4078    0.0001 C   0  0  0  0  0  0  0  0  0  0  0  0
   -2.5906    1.0611   -0.0001 N   0  0  0  0  0  0  0  0  0  0  0  0
   -3.7583    1.6900    0.0002 C   0  0  0  0  0  0  0  0  0  0  0  0
   -4.9634    0.9935    0.0005 C   0  0  0  0  0  0  0  0  0  0  0  0
   -5.0136   -0.3352    0.0005 N   0  0  0  0  0  0  0  0  0  0  0  0
   -3.8446   -0.9644    0.0001 C   0  0  0  0  0  0  0  0  0  0  0  0
    3.4799   -1.3097    0.0005 C   0  0  0  0  0  0  0  0  0  0  0  0
```

4.8027 -0.8877 0.0006 C 0 0 0 0 0 0 0 0 0 0 0 0
5.1233 0.4906 0.0003 C 0 0 0 0 0 0 0 0 0 0 0 0
4.1285 1.4189 -0.0001 C 0 0 0 0 0 0 0 0 0 0 0 0
2.7241 1.0333 -0.0002 C 0 0 0 0 0 0 0 0 0 0 0 0
1.8455 1.8997 -0.0006 O 0 0 0 0 0 0 0 0 0 0 0 0
-0.1389 0.8203 -0.0007 H 0 0 0 0 0 0 0 0 0 0 0 0
1.0022 -2.0173 0.0002 H 0 0 0 0 0 0 0 0 0 0 0 0
-3.7402 2.7720 0.0002 H 0 0 0 0 0 0 0 0 0 0 0 0
-5.9071 1.5236 0.0008 H 0 0 0 0 0 0 0 0 0 0 0 0
-3.8440 -2.0453 0.0001 H 0 0 0 0 0 0 0 0 0 0 0 0
3.2602 -2.3693 0.0007 H 0 0 0 0 0 0 0 0 0 0 0 0
5.5968 -1.6203 0.0008 H 0 0 0 0 0 0 0 0 0 0 0 0
6.1601 0.7973 0.0004 H 0 0 0 0 0 0 0 0 0 0 0 0
4.3253 2.4815 -0.0003 H 0 0 0 0 0 0 0 0 0 0 0 0
1 2 1 0 0 0 0
1 7 4 0 0 0 0
1 11 4 0 0 0 0
2 3 2 0 0 0 0
2 4 1 0 0 0 0
4 5 2 0 0 0 0
4 18 1 0 0 0 0
5 6 1 0 0 0 0
5 19 1 0 0 0 0
6 12 4 0 0 0 0
6 16 4 0 0 0 0
7 8 4 0 0 0 0
8 9 4 0 0 0 0
8 20 1 0 0 0 0
9 10 4 0 0 0 0
9 21 1 0 0 0 0
10 11 4 0 0 0 0
11 22 1 0 0 0 0
12 13 4 0 0 0 0
12 23 1 0 0 0 0

13 14 4 0 0 0 0
13 24 1 0 0 0 0
14 15 4 0 0 0 0
14 25 1 0 0 0 0
15 16 4 0 0 0 0
15 26 1 0 0 0 0
16 17 1 0 0 0 0
M END
$$$$
1a_PR

27 28 0 0 0 0 0 0 0 0 0 V2000
   -2.6246   -0.2866   -0.0110 C   0 0 0 0 0 0 0 0 0 0 0 0
   -1.3785   -1.0757   0.1796 C   0 0 0 0 0 0 0 0 0 0 0 0
   -1.3832   -2.2514   0.4483 O   0 0 0 0 0 0 0 0 0 0 0 0
   -0.0796   -0.3332   0.0247 C   0 0 0 0 0 0 0 0 0 0 0 0
    1.0644   -1.0120   0.2413 C   0 0 0 0 0 0 0 0 0 0 0 0
    2.3990   -0.4880   0.0779 C   0 0 0 0 0 0 0 0 0 0 0 0
   -2.4970   1.0230   -0.2299 N   0 0 0 0 0 0 0 0 0 0 0 0
   -3.6224   1.7017   -0.3907 C   0 0 0 0 0 0 0 0 0 0 0 0
   -4.8668   1.0682   -0.3368 C   0 0 0 0 0 0 0 0 0 0 0 0
   -4.9921   -0.2347   -0.1192 N   0 0 0 0 0 0 0 0 0 0 0 0
   -3.8652   -0.9173   0.0470 C   0 0 0 0 0 0 0 0 0 0 0 0
    3.4371   -1.3178   -0.3395 C   0 0 0 0 0 0 0 0 0 0 0 0
    4.6859   -0.7926   -0.6171 C   0 0 0 0 0 0 0 0 0 0 0 0
    4.9575   0.5973   -0.4443 C   0 0 0 0 0 0 0 0 0 0 0 0
    3.9944   1.4372   0.0226 C   0 0 0 0 0 0 0 0 0 0 0 0
    2.7227   0.9027   0.3420 C   0 0 0 0 0 0 0 0 0 0 0 0
    1.8837   1.7053   0.9345 O   0 0 0 0 0 0 0 0 0 0 0 0
   -0.1345   0.6898   -0.3251 H   0 0 0 0 0 0 0 0 0 0 0 0
    0.9739   -2.0700   0.4707 H   0 0 0 0 0 0 0 0 0 0 0 0
   -3.5470   2.7657   -0.5689 H   0 0 0 0 0 0 0 0 0 0 0 0
   -5.7782   1.6351   -0.4743 H   0 0 0 0 0 0 0 0 0 0 0 0

-3.9332	-1.9814	0.2285	H	0	0	0	0	0	0	0	0	0	0	0	0
3.2377	-2.3681	-0.4983	H	0	0	0	0	0	0	0	0	0	0	0	0
5.4698	-1.4395	-0.9839	H	0	0	0	0	0	0	0	0	0	0	0	0
5.9443	0.9749	-0.6713	H	0	0	0	0	0	0	0	0	0	0	0	0
4.1693	2.4867	0.2097	H	0	0	0	0	0	0	0	0	0	0	0	0
1.0735	1.2535	1.2199	H	0	0	0	0	0	0	0	0	0	0	0	0

1	2	1	0	0	0	0
1	7	4	0	0	0	0
1	11	4	0	0	0	0
2	3	2	0	0	0	0
2	4	1	0	0	0	0
4	5	2	0	0	0	0
4	18	1	0	0	0	0
5	6	1	0	0	0	0
5	19	1	0	0	0	0
6	12	4	0	0	0	0
6	16	4	0	0	0	0
7	8	4	0	0	0	0
8	9	4	0	0	0	0
8	20	1	0	0	0	0
9	10	4	0	0	0	0
9	21	1	0	0	0	0
10	11	4	0	0	0	0
11	22	1	0	0	0	0
12	13	4	0	0	0	0
12	23	1	0	0	0	0
13	14	4	0	0	0	0
13	24	1	0	0	0	0
14	15	4	0	0	0	0
14	25	1	0	0	0	0
15	16	4	0	0	0	0
15	26	1	0	0	0	0
16	17	1	0	0	0	0
17	27	1	0	0	0	0

```
M  END
$$$$
3


 29 30  0  0  0  0  0  0  0  0  0 V2000
   -2.9315   -0.1887    0.0054 C   0  0  0  0  0  0  0  0  0  0  0  0
   -1.6664   -0.9897   -0.0206 C   0  0  0  0  0  0  0  0  0  0  0  0
   -1.7168   -2.2042   -0.0135 O   0  0  0  0  0  0  0  0  0  0  0  0
   -0.3767   -0.2630   -0.0758 C   0  0  0  0  0  0  0  0  0  0  0  0
    0.7667   -0.9445    0.0698 C   0  0  0  0  0  0  0  0  0  0  0  0
    2.1271   -0.4186    0.0177 C   0  0  0  0  0  0  0  0  0  0  0  0
   -4.1116   -0.8244   -0.3812 C   0  0  0  0  0  0  0  0  0  0  0  0
   -5.3144   -0.1364   -0.3633 C   0  0  0  0  0  0  0  0  0  0  0  0
   -5.3514    1.1887    0.0608 C   0  0  0  0  0  0  0  0  0  0  0  0
   -4.1829    1.8223    0.4646 C   0  0  0  0  0  0  0  0  0  0  0  0
   -2.9737    1.1387    0.4300 C   0  0  0  0  0  0  0  0  0  0  0  0
    3.1901   -1.2652    0.3521 C   0  0  0  0  0  0  0  0  0  0  0  0
    4.5017   -0.8252    0.3308 C   0  0  0  0  0  0  0  0  0  0  0  0
    4.7746    0.4865   -0.0388 C   0  0  0  0  0  0  0  0  0  0  0  0
    3.7341    1.3475   -0.3854 C   0  0  0  0  0  0  0  0  0  0  0  0
    2.4272    0.8945   -0.3572 C   0  0  0  0  0  0  0  0  0  0  0  0
    6.0760    0.8778   -0.0508 O   0  0  0  0  0  0  0  0  0  0  0  0
   -4.0594   -1.8586   -0.6910 H   0  0  0  0  0  0  0  0  0  0  0  0
   -4.2131    2.8468    0.8091 H   0  0  0  0  0  0  0  0  0  0  0  0
    1.6339    1.5714   -0.6415 H   0  0  0  0  0  0  0  0  0  0  0  0
   -0.3891    0.8025   -0.2512 H   0  0  0  0  0  0  0  0  0  0  0  0
    0.6717   -2.0101    0.2527 H   0  0  0  0  0  0  0  0  0  0  0  0
   -6.2242   -0.6307   -0.6744 H   0  0  0  0  0  0  0  0  0  0  0  0
   -6.2905    1.7246    0.0809 H   0  0  0  0  0  0  0  0  0  0  0  0
   -2.0744    1.6362    0.7639 H   0  0  0  0  0  0  0  0  0  0  0  0
    2.9768   -2.2874    0.6368 H   0  0  0  0  0  0  0  0  0  0  0  0
    5.3227   -1.4773    0.5910 H   0  0  0  0  0  0  0  0  0  0  0  0
    6.1433    1.7921   -0.3328 H   0  0  0  0  0  0  0  0  0  0  0  0
```

```
    3.9517    2.3665   -0.6818 H   0  0  0  0  0  0  0  0  0  0  0  0
  1  2  1  0  0  0  0
  1  7  4  0  0  0  0
  1 11  4  0  0  0  0
  2  3  2  0  0  0  0
  2  4  1  0  0  0  0
  4  5  2  0  0  0  0
  4 21  1  0  0  0  0
  5  6  1  0  0  0  0
  5 22  1  0  0  0  0
  6 12  4  0  0  0  0
  6 16  4  0  0  0  0
  7  8  4  0  0  0  0
  7 18  1  0  0  0  0
  8  9  4  0  0  0  0
  8 23  1  0  0  0  0
  9 10  4  0  0  0  0
  9 24  1  0  0  0  0
 10 11  4  0  0  0  0
 10 19  1  0  0  0  0
 11 25  1  0  0  0  0
 12 13  4  0  0  0  0
 12 26  1  0  0  0  0
 13 14  4  0  0  0  0
 13 27  1  0  0  0  0
 14 15  4  0  0  0  0
 14 17  1  0  0  0  0
 15 16  4  0  0  0  0
 15 29  1  0  0  0  0
 16 20  1  0  0  0  0
 17 28  1  0  0  0  0
M  END
$$$$
3_A
```

```
28 29  0  0  0  0  0  0  0  0  0 V2000
    2.8655    0.1900    0.0380 C   0  0  0  0  0  0  0  0  0  0  0  0
    1.5780    0.9838    0.0925 C   0  0  0  0  0  0  0  0  0  0  0  0
    1.6707    2.2115    0.1740 O   0  0  0  0  0  0  0  0  0  0  0  0
    0.3442    0.2532    0.0203 C   0  0  0  0  0  0  0  0  0  0  0  0
   -0.8569    0.9148    0.1090 C   0  0  0  0  0  0  0  0  0  0  0  0
   -2.1683    0.3981    0.0382 C   0  0  0  0  0  0  0  0  0  0  0  0
    4.0190    0.8520   -0.3821 C   0  0  0  0  0  0  0  0  0  0  0  0
    5.2345    0.1860   -0.4495 C   0  0  0  0  0  0  0  0  0  0  0  0
    5.3182   -1.1523   -0.0774 C   0  0  0  0  0  0  0  0  0  0  0  0
    4.1784   -1.8149    0.3626 C   0  0  0  0  0  0  0  0  0  0  0  0
    2.9589   -1.1490    0.4157 C   0  0  0  0  0  0  0  0  0  0  0  0
   -3.2788    1.2762    0.1672 C   0  0  0  0  0  0  0  0  0  0  0  0
   -4.5711    0.8423    0.1095 C   0  0  0  0  0  0  0  0  0  0  0  0
   -4.8986   -0.5561   -0.0890 C   0  0  0  0  0  0  0  0  0  0  0  0
   -3.7491   -1.4388   -0.2189 C   0  0  0  0  0  0  0  0  0  0  0  0
   -2.4683   -0.9794   -0.1578 C   0  0  0  0  0  0  0  0  0  0  0  0
   -6.0692   -0.9755   -0.1449 O   0  0  0  0  0  0  0  0  0  0  0  0
    3.9304    1.8967   -0.6453 H   0  0  0  0  0  0  0  0  0  0  0  0
    4.2387   -2.8509    0.6693 H   0  0  0  0  0  0  0  0  0  0  0  0
   -1.6503   -1.6823   -0.2630 H   0  0  0  0  0  0  0  0  0  0  0  0
    0.3793   -0.8131   -0.1417 H   0  0  0  0  0  0  0  0  0  0  0  0
   -0.7734    1.9884    0.2566 H   0  0  0  0  0  0  0  0  0  0  0  0
    6.1192    0.7095   -0.7878 H   0  0  0  0  0  0  0  0  0  0  0  0
    6.2657   -1.6729   -0.1235 H   0  0  0  0  0  0  0  0  0  0  0  0
    2.0827   -1.6683    0.7768 H   0  0  0  0  0  0  0  0  0  0  0  0
   -3.0726    2.3313    0.3169 H   0  0  0  0  0  0  0  0  0  0  0  0
   -5.4017    1.5289    0.2095 H   0  0  0  0  0  0  0  0  0  0  0  0
   -3.9630   -2.4894   -0.3694 H   0  0  0  0  0  0  0  0  0  0  0  0
  1  2  1  0  0  0  0
  1  7  4  0  0  0  0
  1 11  4  0  0  0  0
```

```
  2  3  2  0  0  0  0
  2  4  1  0  0  0  0
  4  5  2  0  0  0  0
  4 21  1  0  0  0  0
  5  6  1  0  0  0  0
  5 22  1  0  0  0  0
  6 12  4  0  0  0  0
  6 16  4  0  0  0  0
  7  8  4  0  0  0  0
  7 18  1  0  0  0  0
  8  9  4  0  0  0  0
  8 23  1  0  0  0  0
  9 10  4  0  0  0  0
  9 24  1  0  0  0  0
 10 11  4  0  0  0  0
 10 19  1  0  0  0  0
 11 25  1  0  0  0  0
 12 13  4  0  0  0  0
 12 26  1  0  0  0  0
 13 14  4  0  0  0  0
 13 27  1  0  0  0  0
 14 15  4  0  0  0  0
 14 17  1  0  0  0  0
 15 16  4  0  0  0  0
 15 28  1  0  0  0  0
 16 20  1  0  0  0  0
M  END
$$$$
3_NR


 28 29  0  0  0  0  0  0  0  0  0 V2000
   -2.8650   -0.1956    0.0146 C   0  0  0  0  0  0  0  0  0  0  0  0
   -1.5948   -0.9817    0.0246 C   0  0  0  0  0  0  0  0  0  0  0  0
```

-1.6198	-2.1960	0.0617	O	0	0	0	0	0	0	0	0	0	0	0	0
-0.3053	-0.2494	-0.0398	C	0	0	0	0	0	0	0	0	0	0	0	0
0.8505	-0.9352	0.0968	C	0	0	0	0	0	0	0	0	0	0	0	0
2.1801	-0.3976	0.0354	C	0	0	0	0	0	0	0	0	0	0	0	0
-4.0399	-0.8689	-0.3233	C	0	0	0	0	0	0	0	0	0	0	0	0
-5.2505	-0.1954	-0.3397	C	0	0	0	0	0	0	0	0	0	0	0	0
-5.3002	1.1538	-0.0017	C	0	0	0	0	0	0	0	0	0	0	0	0
-4.1373	1.8260	0.3526	C	0	0	0	0	0	0	0	0	0	0	0	0
-2.9205	1.1560	0.3544	C	0	0	0	0	0	0	0	0	0	0	0	0
3.2748	-1.2851	0.2241	C	0	0	0	0	0	0	0	0	0	0	0	0
4.5612	-0.8450	0.1801	C	0	0	0	0	0	0	0	0	0	0	0	0
4.8616	0.5587	-0.0639	C	0	0	0	0	0	0	0	0	0	0	0	0
3.7213	1.4488	-0.2571	C	0	0	0	0	0	0	0	0	0	0	0	0
2.4486	0.9821	-0.2079	C	0	0	0	0	0	0	0	0	0	0	0	0
6.0202	0.9765	-0.1064	O	0	0	0	0	0	0	0	0	0	0	0	0
-3.9781	-1.9195	-0.5690	H	0	0	0	0	0	0	0	0	0	0	0	0
-4.1773	2.8704	0.6289	H	0	0	0	0	0	0	0	0	0	0	0	0
1.6244	1.6645	-0.3583	H	0	0	0	0	0	0	0	0	0	0	0	0
-0.3187	0.8133	-0.2246	H	0	0	0	0	0	0	0	0	0	0	0	0
0.7599	-2.0019	0.2701	H	0	0	0	0	0	0	0	0	0	0	0	0
-6.1561	-0.7191	-0.6120	H	0	0	0	0	0	0	0	0	0	0	0	0
-6.2452	1.6792	-0.0102	H	0	0	0	0	0	0	0	0	0	0	0	0
-2.0279	1.6884	0.6506	H	0	0	0	0	0	0	0	0	0	0	0	0
3.0602	-2.3303	0.4059	H	0	0	0	0	0	0	0	0	0	0	0	0
5.4027	-1.5078	0.3220	H	0	0	0	0	0	0	0	0	0	0	0	0
3.9453	2.4900	-0.4414	H	0	0	0	0	0	0	0	0	0	0	0	0

1	2	1	0	0	0	0
1	7	4	0	0	0	0
1	11	4	0	0	0	0
2	3	2	0	0	0	0
2	4	1	0	0	0	0
4	5	2	0	0	0	0
4	21	1	0	0	0	0
5	6	1	0	0	0	0

```
  5 22  1  0  0  0  0
  6 12  4  0  0  0  0
  6 16  4  0  0  0  0
  7  8  4  0  0  0  0
  7 18  1  0  0  0  0
  8  9  4  0  0  0  0
  8 23  1  0  0  0  0
  9 10  4  0  0  0  0
  9 24  1  0  0  0  0
 10 11  4  0  0  0  0
 10 19  1  0  0  0  0
 11 25  1  0  0  0  0
 12 13  4  0  0  0  0
 12 26  1  0  0  0  0
 13 14  4  0  0  0  0
 13 27  1  0  0  0  0
 14 15  4  0  0  0  0
 14 17  1  0  0  0  0
 15 16  4  0  0  0  0
 15 28  1  0  0  0  0
 16 20  1  0  0  0  0
M  END
$$$$
3_PR


 29 30  0  0  0  0  0  0  0  0  0 V2000
    2.9259    0.1848   -0.0088 C   0  0  0  0  0  0  0  0  0  0  0  0
    1.6628    0.9458   -0.0417 C   0  0  0  0  0  0  0  0  0  0  0  0
    1.6151    2.1572   -0.0654 O   0  0  0  0  0  0  0  0  0  0  0  0
    0.3567    0.2021   -0.0896 C   0  0  0  0  0  0  0  0  0  0  0  0
   -0.8010    0.9195    0.0812 C   0  0  0  0  0  0  0  0  0  0  0  0
   -2.1151    0.4068    0.0350 C   0  0  0  0  0  0  0  0  0  0  0  0
    4.1095    0.8923   -0.2408 C   0  0  0  0  0  0  0  0  0  0  0  0
```

5.3254	0.2331	-0.2105	C	0	0	0	0	0	0	0	0	0	0	0	0
5.3693	-1.1316	0.0650	C	0	0	0	0	0	0	0	0	0	0	0	0
4.1980	-1.8379	0.3114	C	0	0	0	0	0	0	0	0	0	0	0	0
2.9754	-1.1842	0.2691	C	0	0	0	0	0	0	0	0	0	0	0	0
-3.1986	1.3098	0.2656	C	0	0	0	0	0	0	0	0	0	0	0	0
-4.4900	0.8849	0.2364	C	0	0	0	0	0	0	0	0	0	0	0	0
-4.7572	-0.4810	-0.0280	C	0	0	0	0	0	0	0	0	0	0	0	0
-3.7043	-1.4030	-0.2634	C	0	0	0	0	0	0	0	0	0	0	0	0
-2.4170	-0.9655	-0.2334	C	0	0	0	0	0	0	0	0	0	0	0	0
-6.0198	-0.8366	-0.0442	O	0	0	0	0	0	0	0	0	0	0	0	0
4.0517	1.9519	-0.4449	H	0	0	0	0	0	0	0	0	0	0	0	0
4.2386	-2.8939	0.5366	H	0	0	0	0	0	0	0	0	0	0	0	0
-1.6184	-1.6681	-0.4180	H	0	0	0	0	0	0	0	0	0	0	0	0
0.3509	-0.8564	-0.2980	H	0	0	0	0	0	0	0	0	0	0	0	0
-0.6779	1.9807	0.2677	H	0	0	0	0	0	0	0	0	0	0	0	0
6.2399	0.7769	-0.3984	H	0	0	0	0	0	0	0	0	0	0	0	0
6.3203	-1.6449	0.0911	H	0	0	0	0	0	0	0	0	0	0	0	0
2.0783	-1.7484	0.4822	H	0	0	0	0	0	0	0	0	0	0	0	0
-2.9724	2.3484	0.4654	H	0	0	0	0	0	0	0	0	0	0	0	0
-5.3251	1.5486	0.4073	H	0	0	0	0	0	0	0	0	0	0	0	0
-6.1499	-1.7745	-0.2312	H	0	0	0	0	0	0	0	0	0	0	0	0
-3.9361	-2.4404	-0.4670	H	0	0	0	0	0	0	0	0	0	0	0	0

1	2	1	0	0	0	0
1	7	4	0	0	0	0
1	11	4	0	0	0	0
2	3	2	0	0	0	0
2	4	1	0	0	0	0
4	5	2	0	0	0	0
4	21	1	0	0	0	0
5	6	1	0	0	0	0
5	22	1	0	0	0	0
6	12	4	0	0	0	0
6	16	4	0	0	0	0
7	8	4	0	0	0	0

```
  7 18  1  0  0  0  0
  8  9  4  0  0  0  0
  8 23  1  0  0  0  0
  9 10  4  0  0  0  0
  9 24  1  0  0  0  0
 10 11  4  0  0  0  0
 10 19  1  0  0  0  0
 11 25  1  0  0  0  0
 12 13  4  0  0  0  0
 12 26  1  0  0  0  0
 13 14  4  0  0  0  0
 13 27  1  0  0  0  0
 14 15  4  0  0  0  0
 14 17  1  0  0  0  0
 15 16  4  0  0  0  0
 15 29  1  0  0  0  0
 16 20  1  0  0  0  0
 17 28  1  0  0  0  0
M  END
$$$$
3a


 27 28  0  0  0  0  0  0  0  0  0 V2000
    2.9458    0.1678   -0.0001 C   0  0  0  0  0  0  0  0  0  0  0  0
    1.6799    0.9851   -0.0004 C   0  0  0  0  0  0  0  0  0  0  0  0
    1.7635    2.1974   -0.0005 O   0  0  0  0  0  0  0  0  0  0  0  0
    0.4047    0.2518   -0.0004 C   0  0  0  0  0  0  0  0  0  0  0  0
   -0.7412    0.9478   -0.0001 C   0  0  0  0  0  0  0  0  0  0  0  0
   -2.0983    0.4169   -0.0001 C   0  0  0  0  0  0  0  0  0  0  0  0
    2.8604   -1.1596    0.0001 N   0  0  0  0  0  0  0  0  0  0  0  0
    4.0090   -1.8247    0.0004 C   0  0  0  0  0  0  0  0  0  0  0  0
    5.2351   -1.1675    0.0004 C   0  0  0  0  0  0  0  0  0  0  0  0
    5.3268    0.1591    0.0002 N   0  0  0  0  0  0  0  0  0  0  0  0
```

```
  4.1780    0.8242   -0.0000 C   0  0  0  0  0  0  0  0  0  0  0  0
 -2.3856   -0.9557   -0.0006 C   0  0  0  0  0  0  0  0  0  0  0  0
 -3.6872   -1.4128   -0.0005 C   0  0  0  0  0  0  0  0  0  0  0  0
 -4.7412   -0.4992    0.0001 C   0  0  0  0  0  0  0  0  0  0  0  0
 -6.0018   -1.0051    0.0003 O   0  0  0  0  0  0  0  0  0  0  0  0
 -4.4830    0.8668    0.0007 C   0  0  0  0  0  0  0  0  0  0  0  0
 -3.1699    1.3122    0.0005 C   0  0  0  0  0  0  0  0  0  0  0  0
  0.4498   -0.8258   -0.0005 H   0  0  0  0  0  0  0  0  0  0  0  0
 -0.6516    2.0299    0.0001 H   0  0  0  0  0  0  0  0  0  0  0  0
  3.9581   -2.9058    0.0005 H   0  0  0  0  0  0  0  0  0  0  0  0
  6.1616   -1.7273    0.0006 H   0  0  0  0  0  0  0  0  0  0  0  0
  4.2101    1.9045   -0.0002 H   0  0  0  0  0  0  0  0  0  0  0  0
 -1.5785   -1.6744   -0.0010 H   0  0  0  0  0  0  0  0  0  0  0  0
 -3.9152   -2.4690   -0.0010 H   0  0  0  0  0  0  0  0  0  0  0  0
 -6.6436   -0.2922   -0.0007 H   0  0  0  0  0  0  0  0  0  0  0  0
 -5.3019    1.5754    0.0012 H   0  0  0  0  0  0  0  0  0  0  0  0
 -2.9693    2.3757    0.0009 H   0  0  0  0  0  0  0  0  0  0  0  0
  1  2  1  0  0  0  0
  1  7  4  0  0  0  0
  1 11  4  0  0  0  0
  2  3  2  0  0  0  0
  2  4  1  0  0  0  0
  4  5  2  0  0  0  0
  4 18  1  0  0  0  0
  5  6  1  0  0  0  0
  5 19  1  0  0  0  0
  6 12  4  0  0  0  0
  6 17  4  0  0  0  0
  7  8  4  0  0  0  0
  8  9  4  0  0  0  0
  8 20  1  0  0  0  0
  9 10  4  0  0  0  0
  9 21  1  0  0  0  0
 10 11  4  0  0  0  0
```

```
 11 22  1  0  0  0  0
 12 13  4  0  0  0  0
 12 23  1  0  0  0  0
 13 14  4  0  0  0  0
 13 24  1  0  0  0  0
 14 15  1  0  0  0  0
 14 16  4  0  0  0  0
 15 25  1  0  0  0  0
 16 17  4  0  0  0  0
 16 26  1  0  0  0  0
 17 27  1  0  0  0  0
M  END
$$$$
3a_A


 26 27  0  0  0  0  0  0  0  0  0 V2000
    2.8728    0.1498    0.0002 C   0  0  0  0  0  0  0  0  0  0  0  0
    1.5912    0.9619    0.0004 C   0  0  0  0  0  0  0  0  0  0  0  0
    1.7202    2.1921    0.0009 O   0  0  0  0  0  0  0  0  0  0  0  0
    0.3678    0.2334   -0.0001 C   0  0  0  0  0  0  0  0  0  0  0  0
   -0.8307    0.9146    0.0000 C   0  0  0  0  0  0  0  0  0  0  0  0
   -2.1416    0.4038   -0.0000 C   0  0  0  0  0  0  0  0  0  0  0  0
    2.8374   -1.1803    0.0001 N   0  0  0  0  0  0  0  0  0  0  0  0
    4.0087   -1.8081   -0.0002 C   0  0  0  0  0  0  0  0  0  0  0  0
    5.2145   -1.1216   -0.0003 C   0  0  0  0  0  0  0  0  0  0  0  0
    5.2654    0.2099   -0.0004 N   0  0  0  0  0  0  0  0  0  0  0  0
    4.0952    0.8359   -0.0002 C   0  0  0  0  0  0  0  0  0  0  0  0
   -2.4500   -0.9880    0.0005 C   0  0  0  0  0  0  0  0  0  0  0  0
   -3.7327   -1.4417    0.0004 C   0  0  0  0  0  0  0  0  0  0  0  0
   -4.8762   -0.5403   -0.0001 C   0  0  0  0  0  0  0  0  0  0  0  0
   -6.0486   -0.9541    0.0000 O   0  0  0  0  0  0  0  0  0  0  0  0
   -4.5408    0.8717   -0.0007 C   0  0  0  0  0  0  0  0  0  0  0  0
   -3.2473    1.3006   -0.0005 C   0  0  0  0  0  0  0  0  0  0  0  0
```

0.4215 -0.8432 -0.0009 H 0 0 0 0 0 0 0 0 0 0 0 0
-0.7394 1.9978 -0.0000 H 0 0 0 0 0 0 0 0 0 0 0 0
3.9887 -2.8913 -0.0004 H 0 0 0 0 0 0 0 0 0 0 0 0
6.1570 -1.6551 -0.0006 H 0 0 0 0 0 0 0 0 0 0 0 0
4.0851 1.9161 -0.0002 H 0 0 0 0 0 0 0 0 0 0 0 0
-1.6345 -1.7009 0.0011 H 0 0 0 0 0 0 0 0 0 0 0 0
-3.9545 -2.5013 0.0010 H 0 0 0 0 0 0 0 0 0 0 0 0
-5.3681 1.5693 -0.0012 H 0 0 0 0 0 0 0 0 0 0 0 0
-3.0335 2.3646 -0.0009 H 0 0 0 0 0 0 0 0 0 0 0 0
1 2 1 0 0 0 0
1 7 4 0 0 0 0
1 11 4 0 0 0 0
2 3 2 0 0 0 0
2 4 1 0 0 0 0
4 5 2 0 0 0 0
4 18 1 0 0 0 0
5 6 1 0 0 0 0
5 19 1 0 0 0 0
6 12 4 0 0 0 0
6 17 4 0 0 0 0
7 8 4 0 0 0 0
8 9 4 0 0 0 0
8 20 1 0 0 0 0
9 10 4 0 0 0 0
9 21 1 0 0 0 0
10 11 4 0 0 0 0
11 22 1 0 0 0 0
12 13 4 0 0 0 0
12 23 1 0 0 0 0
13 14 4 0 0 0 0
13 24 1 0 0 0 0
14 15 1 0 0 0 0
14 16 4 0 0 0 0
16 17 4 0 0 0 0

```
 16 25  1  0  0  0  0
 17 26  1  0  0  0  0
M  END
$$$$
3a_NR


 26 27  0  0  0  0  0  0  0  0  0 V2000
    2.8775    0.1784   -0.0000 C   0  0  0  0  0  0  0  0  0  0  0  0
    1.6133    0.9907   -0.0001 C   0  0  0  0  0  0  0  0  0  0  0  0
    1.6783    2.2032   -0.0000 O   0  0  0  0  0  0  0  0  0  0  0  0
    0.3359    0.2548   -0.0003 C   0  0  0  0  0  0  0  0  0  0  0  0
   -0.8209    0.9555   -0.0002 C   0  0  0  0  0  0  0  0  0  0  0  0
   -2.1473    0.4119   -0.0003 C   0  0  0  0  0  0  0  0  0  0  0  0
    2.7875   -1.1491   -0.0001 N   0  0  0  0  0  0  0  0  0  0  0  0
    3.9336   -1.8172    0.0000 C   0  0  0  0  0  0  0  0  0  0  0  0
    5.1616   -1.1613    0.0002 C   0  0  0  0  0  0  0  0  0  0  0  0
    5.2563    0.1645    0.0003 N   0  0  0  0  0  0  0  0  0  0  0  0
    4.1096    0.8334    0.0003 C   0  0  0  0  0  0  0  0  0  0  0  0
   -2.4083   -0.9910   -0.0004 C   0  0  0  0  0  0  0  0  0  0  0  0
   -3.6793   -1.4640   -0.0003 C   0  0  0  0  0  0  0  0  0  0  0  0
   -4.8243   -0.5586    0.0002 C   0  0  0  0  0  0  0  0  0  0  0  0
   -5.9812   -0.9815    0.0006 O   0  0  0  0  0  0  0  0  0  0  0  0
   -4.5306    0.8684    0.0001 C   0  0  0  0  0  0  0  0  0  0  0  0
   -3.2464    1.3151   -0.0000 C   0  0  0  0  0  0  0  0  0  0  0  0
    0.3820   -0.8222   -0.0005 H   0  0  0  0  0  0  0  0  0  0  0  0
   -0.7347    2.0369   -0.0000 H   0  0  0  0  0  0  0  0  0  0  0  0
    3.8806   -2.8980    0.0001 H   0  0  0  0  0  0  0  0  0  0  0  0
    6.0868   -1.7230    0.0002 H   0  0  0  0  0  0  0  0  0  0  0  0
    4.1457    1.9137    0.0005 H   0  0  0  0  0  0  0  0  0  0  0  0
   -1.5784   -1.6829   -0.0007 H   0  0  0  0  0  0  0  0  0  0  0  0
   -3.8985   -2.5225   -0.0004 H   0  0  0  0  0  0  0  0  0  0  0  0
   -5.3762    1.5413    0.0003 H   0  0  0  0  0  0  0  0  0  0  0  0
   -3.0365    2.3769   -0.0000 H   0  0  0  0  0  0  0  0  0  0  0  0
```

```
  1  2  1  0  0  0  0
  1  7  4  0  0  0  0
  1 11  4  0  0  0  0
  2  3  2  0  0  0  0
  2  4  1  0  0  0  0
  4  5  2  0  0  0  0
  4 18  1  0  0  0  0
  5  6  1  0  0  0  0
  5 19  1  0  0  0  0
  6 12  4  0  0  0  0
  6 17  4  0  0  0  0
  7  8  4  0  0  0  0
  8  9  4  0  0  0  0
  8 20  1  0  0  0  0
  9 10  4  0  0  0  0
  9 21  1  0  0  0  0
 10 11  4  0  0  0  0
 11 22  1  0  0  0  0
 12 13  4  0  0  0  0
 12 23  1  0  0  0  0
 13 14  4  0  0  0  0
 13 24  1  0  0  0  0
 14 15  1  0  0  0  0
 14 16  4  0  0  0  0
 16 17  4  0  0  0  0
 16 25  1  0  0  0  0
 17 26  1  0  0  0  0
M  END
$$$$
3a_PR


 27 28  0  0  0  0  0  0  0  0  0 V2000
    2.9393    0.1927   -0.0003 C   0  0  0  0  0  0  0  0  0  0  0  0
```

1.6879 0.9945 -0.0002 C 0 0 0 0 0 0 0 0 0 0 0 0
1.6848 2.2034 0.0001 O 0 0 0 0 0 0 0 0 0 0 0 0
0.3953 0.2401 -0.0002 C 0 0 0 0 0 0 0 0 0 0 0 0
-0.7706 0.9618 0.0004 C 0 0 0 0 0 0 0 0 0 0 0 0
-2.0749 0.4223 0.0006 C 0 0 0 0 0 0 0 0 0 0 0 0
2.8221 -1.1360 -0.0005 N 0 0 0 0 0 0 0 0 0 0 0 0
3.9539 -1.8220 -0.0004 C 0 0 0 0 0 0 0 0 0 0 0 0
5.1946 -1.1786 -0.0002 C 0 0 0 0 0 0 0 0 0 0 0 0
5.3097 0.1432 0.0001 N 0 0 0 0 0 0 0 0 0 0 0 0
4.1757 0.8343 0.0002 C 0 0 0 0 0 0 0 0 0 0 0 0
-2.3481 -0.9824 0.0012 C 0 0 0 0 0 0 0 0 0 0 0 0
-3.6265 -1.4399 0.0008 C 0 0 0 0 0 0 0 0 0 0 0 0
-4.7004 -0.5104 -0.0002 C 0 0 0 0 0 0 0 0 0 0 0 0
-5.9077 -1.0210 -0.0007 O 0 0 0 0 0 0 0 0 0 0 0 0
-4.4626 0.8864 -0.0006 C 0 0 0 0 0 0 0 0 0 0 0 0
-3.1770 1.3331 -0.0000 C 0 0 0 0 0 0 0 0 0 0 0 0
0.4433 -0.8380 -0.0006 H 0 0 0 0 0 0 0 0 0 0 0 0
-0.6673 2.0419 0.0006 H 0 0 0 0 0 0 0 0 0 0 0 0
3.8875 -2.9015 -0.0004 H 0 0 0 0 0 0 0 0 0 0 0 0
6.1113 -1.7538 -0.0004 H 0 0 0 0 0 0 0 0 0 0 0 0
4.2348 1.9144 0.0007 H 0 0 0 0 0 0 0 0 0 0 0 0
-1.5321 -1.6892 0.0022 H 0 0 0 0 0 0 0 0 0 0 0 0
-3.8650 -2.4937 0.0013 H 0 0 0 0 0 0 0 0 0 0 0 0
-6.6046 -0.3527 -0.0014 H 0 0 0 0 0 0 0 0 0 0 0 0
-5.2964 1.5764 -0.0012 H 0 0 0 0 0 0 0 0 0 0 0 0
-2.9712 2.3949 -0.0001 H 0 0 0 0 0 0 0 0 0 0 0 0
1 2 1 0 0 0 0
1 7 4 0 0 0 0
1 11 4 0 0 0 0
2 3 2 0 0 0 0
2 4 1 0 0 0 0
4 5 2 0 0 0 0
4 18 1 0 0 0 0
5 6 1 0 0 0 0

```
  5 19  1  0  0  0  0
  6 12  4  0  0  0  0
  6 17  4  0  0  0  0
  7  8  4  0  0  0  0
  8  9  4  0  0  0  0
  8 20  1  0  0  0  0
  9 10  4  0  0  0  0
  9 21  1  0  0  0  0
 10 11  4  0  0  0  0
 11 22  1  0  0  0  0
 12 13  4  0  0  0  0
 12 23  1  0  0  0  0
 13 14  4  0  0  0  0
 13 24  1  0  0  0  0
 14 15  1  0  0  0  0
 14 16  4  0  0  0  0
 15 25  1  0  0  0  0
 16 17  4  0  0  0  0
 16 26  1  0  0  0  0
 17 27  1  0  0  0  0
M  END
$$$$
2


 29 30  0  0  0  0  0  0  0  0  0 V2000
   -2.8812   -0.2591    0.0106 C   0  0  0  0  0  0  0  0  0  0  0  0
   -1.5497   -0.9415   -0.0268 C   0  0  0  0  0  0  0  0  0  0  0  0
   -1.4853   -2.1546   -0.0108 O   0  0  0  0  0  0  0  0  0  0  0  0
   -0.3283   -0.1010   -0.1040 C   0  0  0  0  0  0  0  0  0  0  0  0
    0.8714   -0.6769    0.0227 C   0  0  0  0  0  0  0  0  0  0  0  0
    2.1793   -0.0213   -0.0438 C   0  0  0  0  0  0  0  0  0  0  0  0
   -4.0076   -1.0148   -0.3174 C   0  0  0  0  0  0  0  0  0  0  0  0
   -5.2676   -0.4392   -0.2890 C   0  0  0  0  0  0  0  0  0  0  0  0
```

-5.4152 0.8932 0.0856 C 0 0 0 0 0 0 0 0 0 0 0 0
-4.3004 1.6465 0.4306 C 0 0 0 0 0 0 0 0 0 0 0 0
-3.0348 1.0748 0.3869 C 0 0 0 0 0 0 0 0 0 0 0 0
2.3385 1.3441 -0.2941 C 0 0 0 0 0 0 0 0 0 0 0 0
3.6107 1.8939 -0.3394 C 0 0 0 0 0 0 0 0 0 0 0 0
4.7379 1.1076 -0.1409 C 0 0 0 0 0 0 0 0 0 0 0 0
4.5839 -0.2510 0.1054 C 0 0 0 0 0 0 0 0 0 0 0 0
3.3141 -0.8119 0.1533 C 0 0 0 0 0 0 0 0 0 0 0 0
5.7137 -0.9890 0.2924 O 0 0 0 0 0 0 0 0 0 0 0 0
-3.8685 -2.0508 -0.5913 H 0 0 0 0 0 0 0 0 0 0 0 0
-4.4155 2.6772 0.7354 H 0 0 0 0 0 0 0 0 0 0 0 0
3.1968 -1.8723 0.3461 H 0 0 0 0 0 0 0 0 0 0 0 0
-0.4360 0.9577 -0.2840 H 0 0 0 0 0 0 0 0 0 0 0 0
0.8777 -1.7484 0.1938 H 0 0 0 0 0 0 0 0 0 0 0 0
-6.1360 -1.0261 -0.5548 H 0 0 0 0 0 0 0 0 0 0 0 0
-6.3989 1.3415 0.1127 H 0 0 0 0 0 0 0 0 0 0 0 0
-2.1782 1.6673 0.6750 H 0 0 0 0 0 0 0 0 0 0 0 0
1.4774 1.9752 -0.4562 H 0 0 0 0 0 0 0 0 0 0 0 0
3.7312 2.9506 -0.5340 H 0 0 0 0 0 0 0 0 0 0 0 0
5.7344 1.5235 -0.1748 H 0 0 0 0 0 0 0 0 0 0 0 0
5.4835 -1.9082 0.4405 H 0 0 0 0 0 0 0 0 0 0 0 0
1 2 1 0 0 0 0
1 7 4 0 0 0 0
1 11 4 0 0 0 0
2 3 2 0 0 0 0
2 4 1 0 0 0 0
4 5 2 0 0 0 0
4 21 1 0 0 0 0
5 6 1 0 0 0 0
5 22 1 0 0 0 0
6 12 4 0 0 0 0
6 16 4 0 0 0 0
7 8 4 0 0 0 0
7 18 1 0 0 0 0

```
  8  9  4  0  0  0  0
  8 23  1  0  0  0  0
  9 10  4  0  0  0  0
  9 24  1  0  0  0  0
 10 11  4  0  0  0  0
 10 19  1  0  0  0  0
 11 25  1  0  0  0  0
 12 13  4  0  0  0  0
 12 26  1  0  0  0  0
 13 14  4  0  0  0  0
 13 27  1  0  0  0  0
 14 15  4  0  0  0  0
 14 28  1  0  0  0  0
 15 16  4  0  0  0  0
 15 17  1  0  0  0  0
 16 20  1  0  0  0  0
 17 29  1  0  0  0  0
M  END
$$$$
2_A


 28 29  0  0  0  0  0  0  0  0  0 V2000
   -2.8224   -0.2613    0.0253 C   0  0  0  0  0  0  0  0  0  0  0  0
   -1.4911   -0.9728    0.0096 C   0  0  0  0  0  0  0  0  0  0  0  0
   -1.4871   -2.1942    0.0551 O   0  0  0  0  0  0  0  0  0  0  0  0
   -0.2875   -0.1525   -0.0789 C   0  0  0  0  0  0  0  0  0  0  0  0
    0.9358   -0.7136    0.0301 C   0  0  0  0  0  0  0  0  0  0  0  0
    2.2261   -0.0512   -0.0478 C   0  0  0  0  0  0  0  0  0  0  0  0
   -3.9497   -0.9782   -0.3743 C   0  0  0  0  0  0  0  0  0  0  0  0
   -5.2018   -0.3817   -0.3705 C   0  0  0  0  0  0  0  0  0  0  0  0
   -5.3435    0.9365    0.0532 C   0  0  0  0  0  0  0  0  0  0  0  0
   -4.2280    1.6511    0.4716 C   0  0  0  0  0  0  0  0  0  0  0  0
   -2.9710    1.0574    0.4513 C   0  0  0  0  0  0  0  0  0  0  0  0
```

2.3497	1.3258	-0.3139	C	0	0	0	0	0	0	0	0	0	0	0	0
3.6323	1.8622	-0.3608	C	0	0	0	0	0	0	0	0	0	0	0	0
4.7638	1.0883	-0.1545	C	0	0	0	0	0	0	0	0	0	0	0	0
4.7002	-0.3210	0.1206	C	0	0	0	0	0	0	0	0	0	0	0	0
3.3575	-0.8382	0.1581	C	0	0	0	0	0	0	0	0	0	0	0	0
5.7161	-1.0398	0.3111	O	0	0	0	0	0	0	0	0	0	0	0	0
-3.8148	-2.0060	-0.6805	H	0	0	0	0	0	0	0	0	0	0	0	0
-4.3351	2.6709	0.8159	H	0	0	0	0	0	0	0	0	0	0	0	0
3.2454	-1.8973	0.3606	H	0	0	0	0	0	0	0	0	0	0	0	0
-0.3966	0.9069	-0.2528	H	0	0	0	0	0	0	0	0	0	0	0	0
0.9588	-1.7851	0.2032	H	0	0	0	0	0	0	0	0	0	0	0	0
-6.0689	-0.9426	-0.6932	H	0	0	0	0	0	0	0	0	0	0	0	0
-6.3201	1.4021	0.0621	H	0	0	0	0	0	0	0	0	0	0	0	0
-2.1103	1.6155	0.7916	H	0	0	0	0	0	0	0	0	0	0	0	0
1.4839	1.9480	-0.4868	H	0	0	0	0	0	0	0	0	0	0	0	0
3.7533	2.9205	-0.5677	H	0	0	0	0	0	0	0	0	0	0	0	0
5.7505	1.5334	-0.1976	H	0	0	0	0	0	0	0	0	0	0	0	0

1	2	1	0	0	0	0
1	7	4	0	0	0	0
1	11	4	0	0	0	0
2	3	2	0	0	0	0
2	4	1	0	0	0	0
4	5	2	0	0	0	0
4	21	1	0	0	0	0
5	6	1	0	0	0	0
5	22	1	0	0	0	0
6	12	4	0	0	0	0
6	16	4	0	0	0	0
7	8	4	0	0	0	0
7	18	1	0	0	0	0
8	9	4	0	0	0	0
8	23	1	0	0	0	0
9	10	4	0	0	0	0
9	24	1	0	0	0	0

```
 10 11  4  0  0  0  0
 10 19  1  0  0  0  0
 11 25  1  0  0  0  0
 12 13  4  0  0  0  0
 12 26  1  0  0  0  0
 13 14  4  0  0  0  0
 13 27  1  0  0  0  0
 14 15  4  0  0  0  0
 14 28  1  0  0  0  0
 15 16  4  0  0  0  0
 15 17  1  0  0  0  0
 16 20  1  0  0  0  0
M  END
$$$$
2_NR


 28 29  0  0  0  0  0  0  0  0  0 V2000
   -2.8341   -0.2526    0.0098 C   0  0  0  0  0  0  0  0  0  0  0  0
   -1.5167   -0.9575   -0.0420 C   0  0  0  0  0  0  0  0  0  0  0  0
   -1.4680   -2.1700   -0.0461 O   0  0  0  0  0  0  0  0  0  0  0  0
   -0.2771   -0.1368   -0.1114 C   0  0  0  0  0  0  0  0  0  0  0  0
    0.9101   -0.7364    0.0155 C   0  0  0  0  0  0  0  0  0  0  0  0
    2.2297   -0.1026   -0.0460 C   0  0  0  0  0  0  0  0  0  0  0  0
   -3.9737   -0.9817   -0.3322 C   0  0  0  0  0  0  0  0  0  0  0  0
   -5.2228   -0.3837   -0.2923 C   0  0  0  0  0  0  0  0  0  0  0  0
   -5.3457    0.9438    0.1081 C   0  0  0  0  0  0  0  0  0  0  0  0
   -4.2176    1.6699    0.4677 C   0  0  0  0  0  0  0  0  0  0  0  0
   -2.9628    1.0765    0.4122 C   0  0  0  0  0  0  0  0  0  0  0  0
    2.4014    1.2706   -0.3480 C   0  0  0  0  0  0  0  0  0  0  0  0
    3.6722    1.8540   -0.3948 C   0  0  0  0  0  0  0  0  0  0  0  0
    4.7940    1.1015   -0.1504 C   0  0  0  0  0  0  0  0  0  0  0  0
    4.6788   -0.3076    0.1576 C   0  0  0  0  0  0  0  0  0  0  0  0
    3.3458   -0.8703    0.1950 C   0  0  0  0  0  0  0  0  0  0  0  0
```

```
    5.6815   -1.0067    0.3825 O   0  0  0  0  0  0  0  0  0  0  0  0
   -3.8539   -2.0146   -0.6265 H   0  0  0  0  0  0  0  0  0  0  0  0
   -4.3144    2.6960    0.7937 H   0  0  0  0  0  0  0  0  0  0  0  0
    3.2666   -1.9244    0.4241 H   0  0  0  0  0  0  0  0  0  0  0  0
   -0.3695    0.9248   -0.2849 H   0  0  0  0  0  0  0  0  0  0  0  0
    0.9026   -1.8076    0.1855 H   0  0  0  0  0  0  0  0  0  0  0  0
   -6.1018   -0.9493   -0.5688 H   0  0  0  0  0  0  0  0  0  0  0  0
   -6.3210    1.4094    0.1444 H   0  0  0  0  0  0  0  0  0  0  0  0
   -2.0956    1.6473    0.7129 H   0  0  0  0  0  0  0  0  0  0  0  0
    1.5374    1.8843   -0.5551 H   0  0  0  0  0  0  0  0  0  0  0  0
    3.7626    2.9054   -0.6291 H   0  0  0  0  0  0  0  0  0  0  0  0
    5.7899    1.5194   -0.1793 H   0  0  0  0  0  0  0  0  0  0  0  0
  1  2  1  0  0  0  0
  1  7  4  0  0  0  0
  1 11  4  0  0  0  0
  2  3  2  0  0  0  0
  2  4  1  0  0  0  0
  4  5  2  0  0  0  0
  4 21  1  0  0  0  0
  5  6  1  0  0  0  0
  5 22  1  0  0  0  0
  6 12  4  0  0  0  0
  6 16  4  0  0  0  0
  7  8  4  0  0  0  0
  7 18  1  0  0  0  0
  8  9  4  0  0  0  0
  8 23  1  0  0  0  0
  9 10  4  0  0  0  0
  9 24  1  0  0  0  0
 10 11  4  0  0  0  0
 10 19  1  0  0  0  0
 11 25  1  0  0  0  0
 12 13  4  0  0  0  0
 12 26  1  0  0  0  0
```

```
 13 14  4  0  0  0  0
 13 27  1  0  0  0  0
 14 15  4  0  0  0  0
 14 28  1  0  0  0  0
 15 16  4  0  0  0  0
 15 17  1  0  0  0  0
 16 20  1  0  0  0  0
M  END
$$$$
2_PR


 29 30  0  0  0  0  0  0  0  0  0 V2000
   -2.8741   -0.2529   -0.0156 C   0  0  0  0  0  0  0  0  0  0  0  0
   -1.5628   -0.9213   -0.1056 C   0  0  0  0  0  0  0  0  0  0  0  0
   -1.4305   -2.1234   -0.1732 O   0  0  0  0  0  0  0  0  0  0  0  0
   -0.3116   -0.0795   -0.1537 C   0  0  0  0  0  0  0  0  0  0  0  0
    0.8831   -0.7047    0.0032 C   0  0  0  0  0  0  0  0  0  0  0  0
    2.1705   -0.0847   -0.0426 C   0  0  0  0  0  0  0  0  0  0  0  0
   -4.0121   -1.0299   -0.2534 C   0  0  0  0  0  0  0  0  0  0  0  0
   -5.2687   -0.4585   -0.1656 C   0  0  0  0  0  0  0  0  0  0  0  0
   -5.3988    0.8859    0.1745 C   0  0  0  0  0  0  0  0  0  0  0  0
   -4.2728    1.6601    0.4273 C   0  0  0  0  0  0  0  0  0  0  0  0
   -3.0100    1.0957    0.3260 C   0  0  0  0  0  0  0  0  0  0  0  0
    2.3257    1.3169   -0.3412 C   0  0  0  0  0  0  0  0  0  0  0  0
    3.5786    1.8972   -0.3807 C   0  0  0  0  0  0  0  0  0  0  0  0
    4.6947    1.1196   -0.1320 C   0  0  0  0  0  0  0  0  0  0  0  0
    4.5540   -0.2754    0.1609 C   0  0  0  0  0  0  0  0  0  0  0  0
    5.6923   -0.9109    0.3762 O   0  0  0  0  0  0  0  0  0  0  0  0
    3.3033   -0.8638    0.2026 C   0  0  0  0  0  0  0  0  0  0  0  0
   -0.3947    0.9787   -0.3481 H   0  0  0  0  0  0  0  0  0  0  0  0
    0.8474   -1.7736    0.1820 H   0  0  0  0  0  0  0  0  0  0  0  0
   -3.8874   -2.0730   -0.5068 H   0  0  0  0  0  0  0  0  0  0  0  0
   -6.1483   -1.0557   -0.3582 H   0  0  0  0  0  0  0  0  0  0  0  0
```

-6.3820	1.3300	0.2456	H	0	0	0	0	0	0	0	0	0	0	0	0
-4.3796	2.6995	0.7026	H	0	0	0	0	0	0	0	0	0	0	0	0
-2.1483	1.7110	0.5444	H	0	0	0	0	0	0	0	0	0	0	0	0
1.4544	1.9194	-0.5439	H	0	0	0	0	0	0	0	0	0	0	0	0
3.6894	2.9473	-0.6061	H	0	0	0	0	0	0	0	0	0	0	0	0
5.6956	1.5279	-0.1523	H	0	0	0	0	0	0	0	0	0	0	0	0
5.5699	-1.8481	0.5692	H	0	0	0	0	0	0	0	0	0	0	0	0
3.1951	-1.9176	0.4232	H	0	0	0	0	0	0	0	0	0	0	0	0

1	2	1	0	0	0	0
1	7	4	0	0	0	0
1	11	4	0	0	0	0
2	3	2	0	0	0	0
2	4	1	0	0	0	0
4	5	2	0	0	0	0
4	18	1	0	0	0	0
5	6	1	0	0	0	0
5	19	1	0	0	0	0
6	12	4	0	0	0	0
6	17	4	0	0	0	0
7	8	4	0	0	0	0
7	20	1	0	0	0	0
8	9	4	0	0	0	0
8	21	1	0	0	0	0
9	10	4	0	0	0	0
9	22	1	0	0	0	0
10	11	4	0	0	0	0
10	23	1	0	0	0	0
11	24	1	0	0	0	0
12	13	4	0	0	0	0
12	25	1	0	0	0	0
13	14	4	0	0	0	0
13	26	1	0	0	0	0
14	15	4	0	0	0	0
14	27	1	0	0	0	0

```
 15 16  1  0  0  0  0
 15 17  4  0  0  0  0
 16 28  1  0  0  0  0
 17 29  1  0  0  0  0
M  END
$$$$
2a


 27 28  0  0  0  0  0  0  0  0  0 V2000
   -2.8948   -0.2479   -0.0000 C   0  0  0  0  0  0  0  0  0  0  0  0
   -1.5647   -0.9537   -0.0001 C   0  0  0  0  0  0  0  0  0  0  0  0
   -1.5385   -2.1674   -0.0002 O   0  0  0  0  0  0  0  0  0  0  0  0
   -0.3558   -0.1094   -0.0000 C   0  0  0  0  0  0  0  0  0  0  0  0
    0.8463   -0.6976   -0.0001 C   0  0  0  0  0  0  0  0  0  0  0  0
    2.1490   -0.0310   -0.0001 C   0  0  0  0  0  0  0  0  0  0  0  0
   -2.9180    1.0820    0.0003 N   0  0  0  0  0  0  0  0  0  0  0  0
   -4.1165    1.6512    0.0004 C   0  0  0  0  0  0  0  0  0  0  0  0
   -5.2849    0.8955    0.0002 C   0  0  0  0  0  0  0  0  0  0  0  0
   -5.2679   -0.4340   -0.0001 N   0  0  0  0  0  0  0  0  0  0  0  0
   -4.0687   -1.0034   -0.0003 C   0  0  0  0  0  0  0  0  0  0  0  0
    2.2909    1.3589   -0.0003 C   0  0  0  0  0  0  0  0  0  0  0  0
    3.5579    1.9221   -0.0004 C   0  0  0  0  0  0  0  0  0  0  0  0
    4.6952    1.1252   -0.0001 C   0  0  0  0  0  0  0  0  0  0  0  0
    4.5580   -0.2575    0.0002 C   0  0  0  0  0  0  0  0  0  0  0  0
    5.6987   -1.0021    0.0003 O   0  0  0  0  0  0  0  0  0  0  0  0
    3.2938   -0.8326    0.0001 C   0  0  0  0  0  0  0  0  0  0  0  0
   -0.4969    0.9596    0.0001 H   0  0  0  0  0  0  0  0  0  0  0  0
    0.8602   -1.7829   -0.0002 H   0  0  0  0  0  0  0  0  0  0  0  0
   -4.1541    2.7328    0.0007 H   0  0  0  0  0  0  0  0  0  0  0  0
   -4.0128   -2.0829   -0.0005 H   0  0  0  0  0  0  0  0  0  0  0  0
    1.4193    1.9965   -0.0005 H   0  0  0  0  0  0  0  0  0  0  0  0
    3.6665    2.9979   -0.0006 H   0  0  0  0  0  0  0  0  0  0  0  0
    5.6878    1.5515   -0.0000 H   0  0  0  0  0  0  0  0  0  0  0  0
```

```
   5.4812   -1.9362    0.0025 H   0  0  0  0  0  0  0  0  0  0  0  0
   3.1885   -1.9117    0.0001 H   0  0  0  0  0  0  0  0  0  0  0  0
  -6.2539    1.3776    0.0004 H   0  0  0  0  0  0  0  0  0  0  0  0
  1  2  1  0  0  0  0
  1  7  4  0  0  0  0
  1 11  4  0  0  0  0
  2  3  2  0  0  0  0
  2  4  1  0  0  0  0
  4  5  2  0  0  0  0
  4 18  1  0  0  0  0
  5  6  1  0  0  0  0
  5 19  1  0  0  0  0
  6 12  4  0  0  0  0
  6 17  4  0  0  0  0
  7  8  4  0  0  0  0
  8  9  4  0  0  0  0
  8 20  1  0  0  0  0
  9 10  4  0  0  0  0
  9 27  1  0  0  0  0
 10 11  4  0  0  0  0
 11 21  1  0  0  0  0
 12 13  4  0  0  0  0
 12 22  1  0  0  0  0
 13 14  4  0  0  0  0
 13 23  1  0  0  0  0
 14 15  4  0  0  0  0
 14 24  1  0  0  0  0
 15 16  1  0  0  0  0
 15 17  4  0  0  0  0
 16 25  1  0  0  0  0
 17 26  1  0  0  0  0
M  END
$$$$
2a_A
```

```
26 27  0  0  0  0  0  0  0  0  0 V2000
    2.8367   -0.2292   -0.0001 C   0  0  0  0  0  0  0  0  0  0  0  0
    1.5013   -0.9493   -0.0000 C   0  0  0  0  0  0  0  0  0  0  0  0
    1.5287   -2.1726    0.0000 O   0  0  0  0  0  0  0  0  0  0  0  0
    0.3110   -0.1250   -0.0000 C   0  0  0  0  0  0  0  0  0  0  0  0
   -0.9124   -0.7051    0.0001 C   0  0  0  0  0  0  0  0  0  0  0  0
   -2.2033   -0.0471    0.0001 C   0  0  0  0  0  0  0  0  0  0  0  0
    2.8771    1.0994   -0.0002 N   0  0  0  0  0  0  0  0  0  0  0  0
    4.0828    1.6572   -0.0002 C   0  0  0  0  0  0  0  0  0  0  0  0
    5.2445    0.8970   -0.0001 C   0  0  0  0  0  0  0  0  0  0  0  0
    5.2166   -0.4343   -0.0000 N   0  0  0  0  0  0  0  0  0  0  0  0
    4.0113   -0.9902    0.0000 C   0  0  0  0  0  0  0  0  0  0  0  0
   -2.3288    1.3555    0.0001 C   0  0  0  0  0  0  0  0  0  0  0  0
   -3.6132    1.8875    0.0001 C   0  0  0  0  0  0  0  0  0  0  0  0
   -4.7439    1.0846    0.0001 C   0  0  0  0  0  0  0  0  0  0  0  0
   -4.6791   -0.3515    0.0001 C   0  0  0  0  0  0  0  0  0  0  0  0
   -5.6957   -1.0934    0.0000 O   0  0  0  0  0  0  0  0  0  0  0  0
   -3.3347   -0.8633    0.0001 C   0  0  0  0  0  0  0  0  0  0  0  0
    0.4434    0.9445   -0.0001 H   0  0  0  0  0  0  0  0  0  0  0  0
   -0.9313   -1.7908    0.0001 H   0  0  0  0  0  0  0  0  0  0  0  0
    4.1274    2.7392   -0.0003 H   0  0  0  0  0  0  0  0  0  0  0  0
    3.9401   -2.0684    0.0001 H   0  0  0  0  0  0  0  0  0  0  0  0
   -1.4608    1.9981    0.0001 H   0  0  0  0  0  0  0  0  0  0  0  0
   -3.7376    2.9654    0.0001 H   0  0  0  0  0  0  0  0  0  0  0  0
   -5.7326    1.5274    0.0001 H   0  0  0  0  0  0  0  0  0  0  0  0
   -3.2189   -1.9412    0.0001 H   0  0  0  0  0  0  0  0  0  0  0  0
    6.2174    1.3723   -0.0001 H   0  0  0  0  0  0  0  0  0  0  0  0
  1  2  1  0  0  0  0
  1  7  4  0  0  0  0
  1 11  4  0  0  0  0
  2  3  2  0  0  0  0
  2  4  1  0  0  0  0
```

```
  4  5  2  0  0  0  0
  4 18  1  0  0  0  0
  5  6  1  0  0  0  0
  5 19  1  0  0  0  0
  6 12  4  0  0  0  0
  6 17  4  0  0  0  0
  7  8  4  0  0  0  0
  8  9  4  0  0  0  0
  8 20  1  0  0  0  0
  9 10  4  0  0  0  0
  9 26  1  0  0  0  0
 10 11  4  0  0  0  0
 11 21  1  0  0  0  0
 12 13  4  0  0  0  0
 12 22  1  0  0  0  0
 13 14  4  0  0  0  0
 13 23  1  0  0  0  0
 14 15  4  0  0  0  0
 14 24  1  0  0  0  0
 15 16  1  0  0  0  0
 15 17  4  0  0  0  0
 17 25  1  0  0  0  0
M  END
$$$$
2a_NR


 26 27  0  0  0  0  0  0  0  0  0 V2000
    2.8472   -0.2433   -0.0001 C   0  0  0  0  0  0  0  0  0  0  0  0
    1.5323   -0.9741    0.0000 C   0  0  0  0  0  0  0  0  0  0  0  0
    1.5228   -2.1863    0.0001 O   0  0  0  0  0  0  0  0  0  0  0  0
    0.3048   -0.1505   -0.0000 C   0  0  0  0  0  0  0  0  0  0  0  0
   -0.8851   -0.7605    0.0001 C   0  0  0  0  0  0  0  0  0  0  0  0
   -2.1984   -0.1128    0.0001 C   0  0  0  0  0  0  0  0  0  0  0  0
```

```
  2.8421    1.0871   -0.0002 N   0  0  0  0  0  0  0  0  0  0  0  0
  4.0283    1.6813   -0.0002 C   0  0  0  0  0  0  0  0  0  0  0  0
  5.2121    0.9494   -0.0002 C   0  0  0  0  0  0  0  0  0  0  0  0
  5.2227   -0.3800   -0.0000 N   0  0  0  0  0  0  0  0  0  0  0  0
  4.0359   -0.9747    0.0000 C   0  0  0  0  0  0  0  0  0  0  0  0
 -2.3465    1.2960    0.0002 C   0  0  0  0  0  0  0  0  0  0  0  0
 -3.6105    1.8953    0.0001 C   0  0  0  0  0  0  0  0  0  0  0  0
 -4.7470    1.1258    0.0000 C   0  0  0  0  0  0  0  0  0  0  0  0
 -4.6554   -0.3184    0.0000 C   0  0  0  0  0  0  0  0  0  0  0  0
 -5.6719   -1.0334   -0.0001 O   0  0  0  0  0  0  0  0  0  0  0  0
 -3.3286   -0.8974    0.0000 C   0  0  0  0  0  0  0  0  0  0  0  0
  0.4317    0.9205   -0.0001 H   0  0  0  0  0  0  0  0  0  0  0  0
 -0.8876   -1.8455    0.0002 H   0  0  0  0  0  0  0  0  0  0  0  0
  4.0438    2.7633   -0.0003 H   0  0  0  0  0  0  0  0  0  0  0  0
  4.0029   -2.0552    0.0001 H   0  0  0  0  0  0  0  0  0  0  0  0
 -1.4682    1.9241    0.0002 H   0  0  0  0  0  0  0  0  0  0  0  0
 -3.6833    2.9739    0.0002 H   0  0  0  0  0  0  0  0  0  0  0  0
 -5.7382    1.5556    0.0000 H   0  0  0  0  0  0  0  0  0  0  0  0
 -3.2669   -1.9774    0.0000 H   0  0  0  0  0  0  0  0  0  0  0  0
  6.1709    1.4515   -0.0002 H   0  0  0  0  0  0  0  0  0  0  0  0
1  2  1  0  0  0  0
1  7  4  0  0  0  0
1 11  4  0  0  0  0
2  3  2  0  0  0  0
2  4  1  0  0  0  0
4  5  2  0  0  0  0
4 18  1  0  0  0  0
5  6  1  0  0  0  0
5 19  1  0  0  0  0
6 12  4  0  0  0  0
6 17  4  0  0  0  0
7  8  4  0  0  0  0
8  9  4  0  0  0  0
8 20  1  0  0  0  0
```

```
  9 10  4  0  0  0  0
  9 26  1  0  0  0  0
 10 11  4  0  0  0  0
 11 21  1  0  0  0  0
 12 13  4  0  0  0  0
 12 22  1  0  0  0  0
 13 14  4  0  0  0  0
 13 23  1  0  0  0  0
 14 15  4  0  0  0  0
 14 24  1  0  0  0  0
 15 16  1  0  0  0  0
 15 17  4  0  0  0  0
 17 25  1  0  0  0  0
M  END
$$$$
2a_PR


 27 28  0  0  0  0  0  0  0  0  0 V2000
   -2.8875   -0.2713   -0.0000 C   0  0  0  0  0  0  0  0  0  0  0  0
   -1.5843   -0.9886    0.0000 C   0  0  0  0  0  0  0  0  0  0  0  0
   -1.5013   -2.1929    0.0000 O   0  0  0  0  0  0  0  0  0  0  0  0
   -0.3446   -0.1405   -0.0000 C   0  0  0  0  0  0  0  0  0  0  0  0
    0.8561   -0.7653    0.0000 C   0  0  0  0  0  0  0  0  0  0  0  0
    2.1330   -0.1140    0.0000 C   0  0  0  0  0  0  0  0  0  0  0  0
   -2.8591    1.0624   -0.0001 N   0  0  0  0  0  0  0  0  0  0  0  0
   -4.0337    1.6725   -0.0001 C   0  0  0  0  0  0  0  0  0  0  0  0
   -5.2292    0.9491   -0.0001 C   0  0  0  0  0  0  0  0  0  0  0  0
   -5.2564   -0.3776   -0.0001 N   0  0  0  0  0  0  0  0  0  0  0  0
   -4.0795   -0.9925   -0.0000 C   0  0  0  0  0  0  0  0  0  0  0  0
    2.2410    1.3246   -0.0001 C   0  0  0  0  0  0  0  0  0  0  0  0
    3.4781    1.9449   -0.0001 C   0  0  0  0  0  0  0  0  0  0  0  0
    4.6221    1.1726    0.0000 C   0  0  0  0  0  0  0  0  0  0  0  0
    4.5264   -0.2581    0.0001 C   0  0  0  0  0  0  0  0  0  0  0  0
```

```
  5.6857   -0.8863    0.0002 O   0  0  0  0  0  0  0  0  0  0  0  0
  3.2911   -0.8871    0.0001 C   0  0  0  0  0  0  0  0  0  0  0  0
 -0.4829    0.9295   -0.0000 H   0  0  0  0  0  0  0  0  0  0  0  0
  0.8465   -1.8500    0.0001 H   0  0  0  0  0  0  0  0  0  0  0  0
 -4.0385    2.7541   -0.0001 H   0  0  0  0  0  0  0  0  0  0  0  0
 -4.0676   -2.0740   -0.0000 H   0  0  0  0  0  0  0  0  0  0  0  0
  1.3444    1.9245   -0.0001 H   0  0  0  0  0  0  0  0  0  0  0  0
  3.5499    3.0222   -0.0001 H   0  0  0  0  0  0  0  0  0  0  0  0
  5.6120    1.6073    0.0000 H   0  0  0  0  0  0  0  0  0  0  0  0
  5.5955   -1.8472    0.0002 H   0  0  0  0  0  0  0  0  0  0  0  0
  3.2216   -1.9669    0.0002 H   0  0  0  0  0  0  0  0  0  0  0  0
 -6.1817    1.4627   -0.0002 H   0  0  0  0  0  0  0  0  0  0  0  0
 1  2  1  0  0  0  0
 1  7  4  0  0  0  0
 1 11  4  0  0  0  0
 2  3  2  0  0  0  0
 2  4  1  0  0  0  0
 4  5  2  0  0  0  0
 4 18  1  0  0  0  0
 5  6  1  0  0  0  0
 5 19  1  0  0  0  0
 6 12  4  0  0  0  0
 6 17  4  0  0  0  0
 7  8  4  0  0  0  0
 8  9  4  0  0  0  0
 8 20  1  0  0  0  0
 9 10  4  0  0  0  0
 9 27  1  0  0  0  0
10 11  4  0  0  0  0
11 21  1  0  0  0  0
12 13  4  0  0  0  0
12 22  1  0  0  0  0
13 14  4  0  0  0  0
13 23  1  0  0  0  0
```

```
 14 15  4  0  0  0  0
 14 24  1  0  0  0  0
 15 16  1  0  0  0  0
 15 17  4  0  0  0  0
 16 25  1  0  0  0  0
 17 26  1  0  0  0  0
M  END
$$$$
 apigenin


 31 33  0  0  0  0  0  0  0  0  0 V2000
    3.5127    0.5110   -0.0092 C   0  0  0  0  0  0  0  0  0  0  0  0
    2.0971    0.4951   -0.0043 C   0  0  0  0  0  0  0  0  0  0  0  0
    1.4381   -0.7381    0.0208 C   0  0  0  0  0  0  0  0  0  0  0  0
    2.1230   -1.9403    0.0430 C   0  0  0  0  0  0  0  0  0  0  0  0
    3.5099   -1.8885    0.0387 C   0  0  0  0  0  0  0  0  0  0  0  0
    4.2122   -0.6805    0.0128 C   0  0  0  0  0  0  0  0  0  0  0  0
    0.0868   -0.7941    0.0261 O   0  0  0  0  0  0  0  0  0  0  0  0
   -0.6948    0.3260    0.0058 C   0  0  0  0  0  0  0  0  0  0  0  0
   -0.1146    1.5525   -0.0232 C   0  0  0  0  0  0  0  0  0  0  0  0
    1.3255    1.7023   -0.0267 C   0  0  0  0  0  0  0  0  0  0  0  0
   -2.1229    0.0056    0.0052 C   0  0  0  0  0  0  0  0  0  0  0  0
   -3.1047    0.9892    0.1678 C   0  0  0  0  0  0  0  0  0  0  0  0
   -4.4480    0.6503    0.1647 C   0  0  0  0  0  0  0  0  0  0  0  0
   -4.8353   -0.6747   -0.0013 C   0  0  0  0  0  0  0  0  0  0  0  0
   -3.8715   -1.6652   -0.1624 C   0  0  0  0  0  0  0  0  0  0  0  0
   -2.5331   -1.3258   -0.1562 C   0  0  0  0  0  0  0  0  0  0  0  0
   -6.1381   -1.0592   -0.0141 O   0  0  0  0  0  0  0  0  0  0  0  0
   -0.8105    2.7123   -0.0551 O   0  0  0  0  0  0  0  0  0  0  0  0
    1.8117    2.8482   -0.0562 O   0  0  0  0  0  0  0  0  0  0  0  0
    4.1618   -3.0745    0.0614 O   0  0  0  0  0  0  0  0  0  0  0  0
    4.1816    1.6662   -0.0349 O   0  0  0  0  0  0  0  0  0  0  0  0
   -0.1354    3.4103   -0.0820 H   0  0  0  0  0  0  0  0  0  0  0  0
```

```
 -1.7918   -2.0998   -0.2801 H   0  0  0  0  0  0  0  0  0  0  0  0
 -4.1905   -2.6895   -0.2901 H   0  0  0  0  0  0  0  0  0  0  0  0
 -6.7066   -0.2969    0.1128 H   0  0  0  0  0  0  0  0  0  0  0  0
 -2.8220    2.0201    0.2976 H   0  0  0  0  0  0  0  0  0  0  0  0
  1.6022   -2.8844    0.0648 H   0  0  0  0  0  0  0  0  0  0  0  0
  5.1105   -2.9298    0.0573 H   0  0  0  0  0  0  0  0  0  0  0  0
  5.2935   -0.6554    0.0089 H   0  0  0  0  0  0  0  0  0  0  0  0
  3.5289    2.3972   -0.0505 H   0  0  0  0  0  0  0  0  0  0  0  0
 -5.1970    1.4223    0.2927 H   0  0  0  0  0  0  0  0  0  0  0  0
  1  2  4  0  0  0  0
  1  6  4  0  0  0  0
  1 21  1  0  0  0  0
  2  3  1  0  0  0  0
  2 10  4  0  0  0  0
  3  4  1  0  0  0  0
  3  7  2  0  0  0  0
  4  5  4  0  0  0  0
  4 27  1  0  0  0  0
  5  6  4  0  0  0  0
  5 20  1  0  0  0  0
  6 29  1  0  0  0  0
  7  8  4  0  0  0  0
  8  9  4  0  0  0  0
  8 11  1  0  0  0  0
  9 10  4  0  0  0  0
  9 18  1  0  0  0  0
 10 19  1  0  0  0  0
 11 12  4  0  0  0  0
 11 16  4  0  0  0  0
 12 13  4  0  0  0  0
 12 26  1  0  0  0  0
 13 14  4  0  0  0  0
 13 31  1  0  0  0  0
 14 15  4  0  0  0  0
```

```
 14 17  1  0  0  0  0
 15 16  4  0  0  0  0
 15 24  1  0  0  0  0
 16 23  1  0  0  0  0
 17 25  1  0  0  0  0
 18 22  1  0  0  0  0
 20 28  1  0  0  0  0
 21 30  1  0  0  0  0
M  END
$$$$
 apigenin_4pA


 30 32  0  0  0  0  0  0  0  0  0 V2000
    2.5727   -1.3657    0.0000 C   0  0  0  0  0  0  0  0  0  0  0  0
    2.1466   -0.0042   -0.0000 C   0  0  0  0  0  0  0  0  0  0  0  0
    3.1714    0.9885   -0.0001 C   0  0  0  0  0  0  0  0  0  0  0  0
    4.4925    0.6527   -0.0001 C   0  0  0  0  0  0  0  0  0  0  0  0
    4.9523   -0.7223   -0.0000 C   0  0  0  0  0  0  0  0  0  0  0  0
    3.8894   -1.7115    0.0000 C   0  0  0  0  0  0  0  0  0  0  0  0
    0.7665    0.3119   -0.0000 C   0  0  0  0  0  0  0  0  0  0  0  0
   -0.0459   -0.8015   -0.0001 O   0  0  0  0  0  0  0  0  0  0  0  0
   -1.3933   -0.7404   -0.0000 C   0  0  0  0  0  0  0  0  0  0  0  0
   -2.0435    0.4891    0.0000 C   0  0  0  0  0  0  0  0  0  0  0  0
   -1.2550    1.6991    0.0000 C   0  0  0  0  0  0  0  0  0  0  0  0
    0.1437    1.5560   -0.0000 C   0  0  0  0  0  0  0  0  0  0  0  0
   -2.0910   -1.9430   -0.0000 C   0  0  0  0  0  0  0  0  0  0  0  0
   -3.4743   -1.8799    0.0000 C   0  0  0  0  0  0  0  0  0  0  0  0
   -4.1669   -0.6678    0.0001 C   0  0  0  0  0  0  0  0  0  0  0  0
   -3.4517    0.5203    0.0001 C   0  0  0  0  0  0  0  0  0  0  0  0
   -4.0946    1.6952    0.0001 O   0  0  0  0  0  0  0  0  0  0  0  0
   -4.1513   -3.0682    0.0000 O   0  0  0  0  0  0  0  0  0  0  0  0
    0.8531    2.7261    0.0000 O   0  0  0  0  0  0  0  0  0  0  0  0
   -1.7909    2.8508    0.0001 O   0  0  0  0  0  0  0  0  0  0  0  0
```

```
 6.1565  -1.0330  -0.0000 O  0 0 0 0 0 0 0 0 0 0 0 0
 0.1664   3.4108   0.0001 H  0 0 0 0 0 0 0 0 0 0 0 0
 1.8227  -2.1438   0.0001 H  0 0 0 0 0 0 0 0 0 0 0 0
 4.1916  -2.7510   0.0001 H  0 0 0 0 0 0 0 0 0 0 0 0
 2.8901   2.0289  -0.0002 H  0 0 0 0 0 0 0 0 0 0 0 0
-1.5731  -2.8891  -0.0000 H  0 0 0 0 0 0 0 0 0 0 0 0
-5.0927  -2.8885   0.0002 H  0 0 0 0 0 0 0 0 0 0 0 0
-5.2486  -0.6336   0.0001 H  0 0 0 0 0 0 0 0 0 0 0 0
-3.3844   2.3952   0.0001 H  0 0 0 0 0 0 0 0 0 0 0 0
 5.2562   1.4198  -0.0002 H  0 0 0 0 0 0 0 0 0 0 0 0
  1  2  4  0  0  0  0
  1  6  4  0  0  0  0
  1 23  1  0  0  0  0
  2  3  4  0  0  0  0
  2  7  1  0  0  0  0
  3  4  4  0  0  0  0
  3 25  1  0  0  0  0
  4  5  4  0  0  0  0
  4 30  1  0  0  0  0
  5  6  4  0  0  0  0
  5 21  1  0  0  0  0
  6 24  1  0  0  0  0
  7  8  4  0  0  0  0
  7 12  4  0  0  0  0
  8  9  2  0  0  0  0
  9 10  1  0  0  0  0
  9 13  1  0  0  0  0
 10 11  4  0  0  0  0
 10 16  4  0  0  0  0
 11 12  4  0  0  0  0
 11 20  1  0  0  0  0
 12 19  1  0  0  0  0
 13 14  4  0  0  0  0
 13 26  1  0  0  0  0
```

```
 14 15  4  0  0  0  0
 14 18  1  0  0  0  0
 15 16  4  0  0  0  0
 15 28  1  0  0  0  0
 16 17  1  0  0  0  0
 17 29  1  0  0  0  0
 18 27  1  0  0  0  0
 19 22  1  0  0  0  0
M  END
$$$$
 apigenin_4pNR


 30 32  0  0  0  0  0  0  0  0  0 V2000
    2.5622   -1.3782   -0.0000 C   0  0  0  0  0  0  0  0  0  0  0  0
    2.1663   -0.0089   -0.0002 C   0  0  0  0  0  0  0  0  0  0  0  0
    3.1701    1.0013   -0.0002 C   0  0  0  0  0  0  0  0  0  0  0  0
    4.4894    0.6661   -0.0000 C   0  0  0  0  0  0  0  0  0  0  0  0
    4.9246   -0.7203    0.0003 C   0  0  0  0  0  0  0  0  0  0  0  0
    3.8759   -1.7276    0.0002 C   0  0  0  0  0  0  0  0  0  0  0  0
    0.7597    0.3110   -0.0003 C   0  0  0  0  0  0  0  0  0  0  0  0
   -0.0389   -0.8021   -0.0005 O   0  0  0  0  0  0  0  0  0  0  0  0
   -1.3898   -0.7366   -0.0003 C   0  0  0  0  0  0  0  0  0  0  0  0
   -2.0454    0.5015   -0.0001 C   0  0  0  0  0  0  0  0  0  0  0  0
   -1.2715    1.7020   -0.0001 C   0  0  0  0  0  0  0  0  0  0  0  0
    0.1769    1.5489   -0.0002 C   0  0  0  0  0  0  0  0  0  0  0  0
   -2.0802   -1.9331   -0.0002 C   0  0  0  0  0  0  0  0  0  0  0  0
   -3.4689   -1.8750    0.0000 C   0  0  0  0  0  0  0  0  0  0  0  0
   -4.1667   -0.6643    0.0002 C   0  0  0  0  0  0  0  0  0  0  0  0
   -3.4623    0.5239    0.0002 C   0  0  0  0  0  0  0  0  0  0  0  0
   -4.1277    1.6801    0.0005 O   0  0  0  0  0  0  0  0  0  0  0  0
   -4.1214   -3.0572    0.0000 O   0  0  0  0  0  0  0  0  0  0  0  0
    0.8584    2.7034   -0.0001 O   0  0  0  0  0  0  0  0  0  0  0  0
   -1.7400    2.8531    0.0002 O   0  0  0  0  0  0  0  0  0  0  0  0
```

```
  6.1205   -1.0280    0.0005 O   0  0  0  0  0  0  0  0  0  0  0  0
  0.1798    3.4020    0.0001 H   0  0  0  0  0  0  0  0  0  0  0  0
  1.8006   -2.1416   -0.0000 H   0  0  0  0  0  0  0  0  0  0  0  0
  4.1904   -2.7616    0.0004 H   0  0  0  0  0  0  0  0  0  0  0  0
  2.8810    2.0380   -0.0004 H   0  0  0  0  0  0  0  0  0  0  0  0
 -1.5655   -2.8806   -0.0004 H   0  0  0  0  0  0  0  0  0  0  0  0
 -5.0707   -2.9147    0.0004 H   0  0  0  0  0  0  0  0  0  0  0  0
 -5.2477   -0.6346    0.0004 H   0  0  0  0  0  0  0  0  0  0  0  0
 -3.4805    2.4134    0.0005 H   0  0  0  0  0  0  0  0  0  0  0  0
  5.2639    1.4200   -0.0001 H   0  0  0  0  0  0  0  0  0  0  0  0
 1  2  4  0  0  0  0
 1  6  4  0  0  0  0
 1 23  1  0  0  0  0
 2  3  4  0  0  0  0
 2  7  1  0  0  0  0
 3  4  4  0  0  0  0
 3 25  1  0  0  0  0
 4  5  4  0  0  0  0
 4 30  1  0  0  0  0
 5  6  4  0  0  0  0
 5 21  1  0  0  0  0
 6 24  1  0  0  0  0
 7  8  4  0  0  0  0
 7 12  4  0  0  0  0
 8  9  2  0  0  0  0
 9 10  1  0  0  0  0
 9 13  1  0  0  0  0
10 11  4  0  0  0  0
10 16  4  0  0  0  0
11 12  4  0  0  0  0
11 20  1  0  0  0  0
12 19  1  0  0  0  0
13 14  4  0  0  0  0
13 26  1  0  0  0  0
```

```
 14 15  4  0  0  0  0
 14 18  1  0  0  0  0
 15 16  4  0  0  0  0
 15 28  1  0  0  0  0
 16 17  1  0  0  0  0
 17 29  1  0  0  0  0
 18 27  1  0  0  0  0
 19 22  1  0  0  0  0
M  END
$$$$
 apigenin_PR


 31 33  0  0  0  0  0  0  0  0  0 V2000
    2.5432   -1.3605    0.0000 C   0  0  0  0  0  0  0  0  0  0  0  0
    2.1220   -0.0003    0.0000 C   0  0  0  0  0  0  0  0  0  0  0  0
    3.1136    1.0171   -0.0000 C   0  0  0  0  0  0  0  0  0  0  0  0
    4.4435    0.6920   -0.0000 C   0  0  0  0  0  0  0  0  0  0  0  0
    4.8340   -0.6588   -0.0000 C   0  0  0  0  0  0  0  0  0  0  0  0
    3.8676   -1.6846    0.0000 C   0  0  0  0  0  0  0  0  0  0  0  0
    0.7308    0.2846    0.0000 C   0  0  0  0  0  0  0  0  0  0  0  0
   -0.0673   -0.7931    0.0000 O   0  0  0  0  0  0  0  0  0  0  0  0
   -1.4395   -0.7339    0.0000 C   0  0  0  0  0  0  0  0  0  0  0  0
   -2.1036    0.5063    0.0000 C   0  0  0  0  0  0  0  0  0  0  0  0
   -1.3513    1.7043   -0.0000 C   0  0  0  0  0  0  0  0  0  0  0  0
    0.1185    1.5508    0.0000 C   0  0  0  0  0  0  0  0  0  0  0  0
   -2.1069   -1.9246   -0.0000 C   0  0  0  0  0  0  0  0  0  0  0  0
   -3.5151   -1.8824   -0.0000 C   0  0  0  0  0  0  0  0  0  0  0  0
   -4.2215   -0.6839   -0.0000 C   0  0  0  0  0  0  0  0  0  0  0  0
   -3.5264    0.5142    0.0000 C   0  0  0  0  0  0  0  0  0  0  0  0
   -4.2034    1.6503    0.0000 O   0  0  0  0  0  0  0  0  0  0  0  0
   -4.1216   -3.0699   -0.0000 O   0  0  0  0  0  0  0  0  0  0  0  0
    0.7808    2.6762    0.0000 O   0  0  0  0  0  0  0  0  0  0  0  0
   -1.7813    2.8612   -0.0000 O   0  0  0  0  0  0  0  0  0  0  0  0
```

6.1014	-1.0384	-0.0000	O	0	0	0	0	0	0	0	0	0	0	0	0
0.1035	3.3906	-0.0000	H	0	0	0	0	0	0	0	0	0	0	0	0
1.8019	-2.1439	0.0000	H	0	0	0	0	0	0	0	0	0	0	0	0
4.2047	-2.7108	0.0000	H	0	0	0	0	0	0	0	0	0	0	0	0
6.7105	-0.2918	-0.0000	H	0	0	0	0	0	0	0	0	0	0	0	0
2.8324	2.0558	-0.0000	H	0	0	0	0	0	0	0	0	0	0	0	0
-1.5913	-2.8722	-0.0000	H	0	0	0	0	0	0	0	0	0	0	0	0
-5.0799	-2.9829	-0.0000	H	0	0	0	0	0	0	0	0	0	0	0	0
-5.3025	-0.6616	-0.0000	H	0	0	0	0	0	0	0	0	0	0	0	0
-3.5967	2.4129	-0.0000	H	0	0	0	0	0	0	0	0	0	0	0	0
5.1944	1.4712	-0.0000	H	0	0	0	0	0	0	0	0	0	0	0	0

1	2	4	0	0	0	0
1	6	4	0	0	0	0
1	23	1	0	0	0	0
2	3	4	0	0	0	0
2	7	1	0	0	0	0
3	4	4	0	0	0	0
3	26	1	0	0	0	0
4	5	4	0	0	0	0
4	31	1	0	0	0	0
5	6	4	0	0	0	0
5	21	1	0	0	0	0
6	24	1	0	0	0	0
7	8	4	0	0	0	0
7	12	4	0	0	0	0
8	9	2	0	0	0	0
9	10	1	0	0	0	0
9	13	1	0	0	0	0
10	11	4	0	0	0	0
10	16	4	0	0	0	0
11	12	4	0	0	0	0
11	20	1	0	0	0	0
12	19	1	0	0	0	0
13	14	4	0	0	0	0

```
 13 27  1  0  0  0  0
 14 15  4  0  0  0  0
 14 18  1  0  0  0  0
 15 16  4  0  0  0  0
 15 29  1  0  0  0  0
 16 17  1  0  0  0  0
 17 30  1  0  0  0  0
 18 28  1  0  0  0  0
 19 22  1  0  0  0  0
 21 25  1  0  0  0  0
M  END
$$$$
 quercetin


 32 34  0  0  0  0  0  0  0  0  0 V2000
   -2.8666    0.7607    0.0663 C   0  0  0  0  0  0  0  0  0  0  0  0
   -1.8358   -0.1880   -0.0138 C   0  0  0  0  0  0  0  0  0  0  0  0
   -2.1715   -1.5428   -0.0986 C   0  0  0  0  0  0  0  0  0  0  0  0
   -3.4972   -1.9462   -0.1072 C   0  0  0  0  0  0  0  0  0  0  0  0
   -4.5123   -1.0073   -0.0279 C   0  0  0  0  0  0  0  0  0  0  0  0
   -4.1813    0.3469    0.0601 C   0  0  0  0  0  0  0  0  0  0  0  0
   -0.4274    0.2098   -0.0074 C   0  0  0  0  0  0  0  0  0  0  0  0
    0.0874    1.4659   -0.0255 C   0  0  0  0  0  0  0  0  0  0  0  0
    1.5173    1.6932   -0.0253 C   0  0  0  0  0  0  0  0  0  0  0  0
    2.3525    0.5297   -0.0064 C   0  0  0  0  0  0  0  0  0  0  0  0
    1.7597   -0.7368    0.0133 C   0  0  0  0  0  0  0  0  0  0  0  0
    0.4135   -0.8651    0.0121 O   0  0  0  0  0  0  0  0  0  0  0  0
    2.5071   -1.9011    0.0355 C   0  0  0  0  0  0  0  0  0  0  0  0
    3.8896   -1.7757    0.0379 C   0  0  0  0  0  0  0  0  0  0  0  0
    4.5267   -0.5318    0.0185 C   0  0  0  0  0  0  0  0  0  0  0  0
    3.7650    0.6210   -0.0043 C   0  0  0  0  0  0  0  0  0  0  0  0
    1.9399    2.8644   -0.0461 O   0  0  0  0  0  0  0  0  0  0  0  0
   -0.6671    2.5903   -0.0492 O   0  0  0  0  0  0  0  0  0  0  0  0
```

```
  4.3708    1.8104   -0.0241 O   0  0  0  0  0  0  0  0  0  0  0  0
  4.6029   -2.9252    0.0607 O   0  0  0  0  0  0  0  0  0  0  0  0
 -5.8066   -1.3970   -0.0354 O   0  0  0  0  0  0  0  0  0  0  0  0
 -5.2534    1.1982    0.1375 O   0  0  0  0  0  0  0  0  0  0  0  0
 -0.0259    3.3203   -0.0678 H   0  0  0  0  0  0  0  0  0  0  0  0
 -1.3919   -2.2846   -0.1607 H   0  0  0  0  0  0  0  0  0  0  0  0
 -3.7640   -2.9909   -0.1754 H   0  0  0  0  0  0  0  0  0  0  0  0
 -6.3625   -0.6135    0.0358 H   0  0  0  0  0  0  0  0  0  0  0  0
 -4.9586    2.1086    0.2029 H   0  0  0  0  0  0  0  0  0  0  0  0
 -2.6402    1.8132    0.1342 H   0  0  0  0  0  0  0  0  0  0  0  0
  2.0369   -2.8711    0.0520 H   0  0  0  0  0  0  0  0  0  0  0  0
  5.5428   -2.7311    0.0613 H   0  0  0  0  0  0  0  0  0  0  0  0
  5.6050   -0.4492    0.0199 H   0  0  0  0  0  0  0  0  0  0  0  0
  3.6797    2.5051   -0.0384 H   0  0  0  0  0  0  0  0  0  0  0  0
  1  2  4  0  0  0  0
  1  6  4  0  0  0  0
  1 28  1  0  0  0  0
  2  3  4  0  0  0  0
  2  7  1  0  0  0  0
  3  4  4  0  0  0  0
  3 24  1  0  0  0  0
  4  5  4  0  0  0  0
  4 25  1  0  0  0  0
  5  6  4  0  0  0  0
  5 21  1  0  0  0  0
  6 22  1  0  0  0  0
  7  8  4  0  0  0  0
  7 12  4  0  0  0  0
  8  9  4  0  0  0  0
  8 18  1  0  0  0  0
  9 10  4  0  0  0  0
  9 17  1  0  0  0  0
 10 11  1  0  0  0  0
 10 16  4  0  0  0  0
```

```
 11 12  2  0  0  0  0
 11 13  1  0  0  0  0
 13 14  4  0  0  0  0
 13 29  1  0  0  0  0
 14 15  4  0  0  0  0
 14 20  1  0  0  0  0
 15 16  4  0  0  0  0
 15 31  1  0  0  0  0
 16 19  1  0  0  0  0
 18 23  1  0  0  0  0
 19 32  1  0  0  0  0
 20 30  1  0  0  0  0
 21 26  1  0  0  0  0
 22 27  1  0  0  0  0
M  END
$$$$
 q_4pA


 31 33  0  0  0  0  0  0  0  0  0 V2000
    2.9221     0.7794    -0.0006 C   0  0  0  0  0  0  0  0  0  0  0  0
    1.8571    -0.1736     0.0002 C   0  0  0  0  0  0  0  0  0  0  0  0
    2.2004    -1.5472     0.0009 C   0  0  0  0  0  0  0  0  0  0  0  0
    3.5059    -1.9717     0.0010 C   0  0  0  0  0  0  0  0  0  0  0  0
    4.5912    -1.0451     0.0003 C   0  0  0  0  0  0  0  0  0  0  0  0
    4.2134     0.3547    -0.0005 C   0  0  0  0  0  0  0  0  0  0  0  0
    0.4867     0.2104     0.0000 C   0  0  0  0  0  0  0  0  0  0  0  0
   -0.0696     1.4815     0.0002 C   0  0  0  0  0  0  0  0  0  0  0  0
   -1.4645     1.6931     0.0000 C   0  0  0  0  0  0  0  0  0  0  0  0
   -2.3102     0.5239     0.0002 C   0  0  0  0  0  0  0  0  0  0  0  0
   -1.7193    -0.7357    -0.0002 C   0  0  0  0  0  0  0  0  0  0  0  0
   -0.3781    -0.8612    -0.0003 O   0  0  0  0  0  0  0  0  0  0  0  0
   -2.4734    -1.9040    -0.0004 C   0  0  0  0  0  0  0  0  0  0  0  0
   -3.8521    -1.7751    -0.0004 C   0  0  0  0  0  0  0  0  0  0  0  0
```

-4.4861	-0.5309	-0.0002	C	0	0	0	0	0	0	0	0	0	0	0	0
-3.7159	0.6218	0.0002	C	0	0	0	0	0	0	0	0	0	0	0	0
-1.9403	2.8681	0.0000	O	0	0	0	0	0	0	0	0	0	0	0	0
0.6937	2.6136	0.0005	O	0	0	0	0	0	0	0	0	0	0	0	0
-4.3036	1.8247	0.0005	O	0	0	0	0	0	0	0	0	0	0	0	0
-4.5845	-2.9289	-0.0007	O	0	0	0	0	0	0	0	0	0	0	0	0
5.8148	-1.3350	0.0002	O	0	0	0	0	0	0	0	0	0	0	0	0
5.2701	1.2151	-0.0012	O	0	0	0	0	0	0	0	0	0	0	0	0
0.0448	3.3335	0.0002	H	0	0	0	0	0	0	0	0	0	0	0	0
1.4103	-2.2829	0.0015	H	0	0	0	0	0	0	0	0	0	0	0	0
3.7479	-3.0262	0.0016	H	0	0	0	0	0	0	0	0	0	0	0	0
6.0333	0.6089	-0.0009	H	0	0	0	0	0	0	0	0	0	0	0	0
2.7182	1.8369	-0.0012	H	0	0	0	0	0	0	0	0	0	0	0	0
-2.0009	-2.8732	-0.0005	H	0	0	0	0	0	0	0	0	0	0	0	0
-5.5166	-2.7054	0.0014	H	0	0	0	0	0	0	0	0	0	0	0	0
-5.5649	-0.4452	-0.0004	H	0	0	0	0	0	0	0	0	0	0	0	0
-3.5647	2.4921	0.0007	H	0	0	0	0	0	0	0	0	0	0	0	0

1	2	4	0	0	0	0
1	6	4	0	0	0	0
1	27	1	0	0	0	0
2	3	4	0	0	0	0
2	7	1	0	0	0	0
3	4	4	0	0	0	0
3	24	1	0	0	0	0
4	5	4	0	0	0	0
4	25	1	0	0	0	0
5	6	4	0	0	0	0
5	21	1	0	0	0	0
6	22	1	0	0	0	0
7	8	4	0	0	0	0
7	12	4	0	0	0	0
8	9	4	0	0	0	0
8	18	1	0	0	0	0
9	10	4	0	0	0	0

```
  9 17  1  0  0  0  0
 10 11  1  0  0  0  0
 10 16  4  0  0  0  0
 11 12  2  0  0  0  0
 11 13  1  0  0  0  0
 13 14  4  0  0  0  0
 13 28  1  0  0  0  0
 14 15  4  0  0  0  0
 14 20  1  0  0  0  0
 15 16  4  0  0  0  0
 15 30  1  0  0  0  0
 16 19  1  0  0  0  0
 18 23  1  0  0  0  0
 19 31  1  0  0  0  0
 20 29  1  0  0  0  0
 22 26  1  0  0  0  0
M  END
$$$$
 q_4pNR


 31 33  0  0  0  0  0  0  0  0  0 V2000
    2.9039    0.7923   -0.0001 C   0  0  0  0  0  0  0  0  0  0  0  0
    1.8793   -0.1646    0.0001 C   0  0  0  0  0  0  0  0  0  0  0  0
    2.2038   -1.5624    0.0002 C   0  0  0  0  0  0  0  0  0  0  0  0
    3.4905   -1.9928    0.0001 C   0  0  0  0  0  0  0  0  0  0  0  0
    4.5786   -1.0481    0.0000 C   0  0  0  0  0  0  0  0  0  0  0  0
    4.2134    0.3693   -0.0001 C   0  0  0  0  0  0  0  0  0  0  0  0
    0.4804    0.2177    0.0001 C   0  0  0  0  0  0  0  0  0  0  0  0
   -0.0416    1.4784    0.0000 C   0  0  0  0  0  0  0  0  0  0  0  0
   -1.4809    1.6988    0.0001 C   0  0  0  0  0  0  0  0  0  0  0  0
   -2.3089    0.5342    0.0002 C   0  0  0  0  0  0  0  0  0  0  0  0
   -1.7109   -0.7323    0.0001 C   0  0  0  0  0  0  0  0  0  0  0  0
   -0.3649   -0.8591    0.0002 O   0  0  0  0  0  0  0  0  0  0  0  0
```

```
-2.4550 -1.8970 0.0000 C 0 0 0 0 0 0 0 0 0 0 0 0
-3.8392 -1.7759 -0.0001 C 0 0 0 0 0 0 0 0 0 0 0 0
-4.4808 -0.5342 -0.0001 C 0 0 0 0 0 0 0 0 0 0 0 0
-3.7232 0.6208 0.0000 C 0 0 0 0 0 0 0 0 0 0 0 0
-1.8979 2.8694 0.0000 O 0 0 0 0 0 0 0 0 0 0 0 0
0.6969 2.5987 -0.0001 O 0 0 0 0 0 0 0 0 0 0 0 0
-4.3341 1.8065 -0.0000 O 0 0 0 0 0 0 0 0 0 0 0 0
-4.5456 -2.9270 -0.0002 O 0 0 0 0 0 0 0 0 0 0 0 0
5.7811 -1.3431 -0.0001 O 0 0 0 0 0 0 0 0 0 0 0 0
5.2267 1.2279 -0.0002 O 0 0 0 0 0 0 0 0 0 0 0 0
0.0550 3.3302 -0.0001 H 0 0 0 0 0 0 0 0 0 0 0 0
1.3987 -2.2790 0.0003 H 0 0 0 0 0 0 0 0 0 0 0 0
3.7427 -3.0435 0.0002 H 0 0 0 0 0 0 0 0 0 0 0 0
6.0356 0.6860 -0.0002 H 0 0 0 0 0 0 0 0 0 0 0 0
2.6954 1.8479 -0.0001 H 0 0 0 0 0 0 0 0 0 0 0 0
-1.9834 -2.8665 -0.0000 H 0 0 0 0 0 0 0 0 0 0 0 0
-5.4873 -2.7407 -0.0006 H 0 0 0 0 0 0 0 0 0 0 0 0
-5.5593 -0.4551 -0.0002 H 0 0 0 0 0 0 0 0 0 0 0 0
-3.6519 2.5080 -0.0001 H 0 0 0 0 0 0 0 0 0 0 0 0
1 2 4 0 0 0 0
1 6 4 0 0 0 0
1 27 1 0 0 0 0
2 3 4 0 0 0 0
2 7 1 0 0 0 0
3 4 4 0 0 0 0
3 24 1 0 0 0 0
4 5 4 0 0 0 0
4 25 1 0 0 0 0
5 6 4 0 0 0 0
5 21 1 0 0 0 0
6 22 1 0 0 0 0
7 8 4 0 0 0 0
7 12 4 0 0 0 0
8 9 4 0 0 0 0
```

```
  8 18  1  0  0  0  0
  9 10  4  0  0  0  0
  9 17  1  0  0  0  0
 10 11  1  0  0  0  0
 10 16  4  0  0  0  0
 11 12  2  0  0  0  0
 11 13  1  0  0  0  0
 13 14  4  0  0  0  0
 13 28  1  0  0  0  0
 14 15  4  0  0  0  0
 14 20  1  0  0  0  0
 15 16  4  0  0  0  0
 15 30  1  0  0  0  0
 16 19  1  0  0  0  0
 18 23  1  0  0  0  0
 19 31  1  0  0  0  0
 20 29  1  0  0  0  0
 22 26  1  0  0  0  0
M  END
$$$$
 q_PR


 32 34  0  0  0  0  0  0  0  0  0 V2000
    3.7762    0.6237   -0.0001 C   0  0  0  0  0  0  0  0  0  0  0  0
    2.3555    0.5397    0.0000 C   0  0  0  0  0  0  0  0  0  0  0  0
    1.7573   -0.7343    0.0000 C   0  0  0  0  0  0  0  0  0  0  0  0
    2.4897   -1.8881    0.0000 C   0  0  0  0  0  0  0  0  0  0  0  0
    3.8909   -1.7703   -0.0000 C   0  0  0  0  0  0  0  0  0  0  0  0
    4.5333   -0.5345   -0.0001 C   0  0  0  0  0  0  0  0  0  0  0  0
    0.3935   -0.8673    0.0001 O   0  0  0  0  0  0  0  0  0  0  0  0
   -0.4590    0.1763    0.0000 C   0  0  0  0  0  0  0  0  0  0  0  0
    0.0807    1.4653    0.0000 C   0  0  0  0  0  0  0  0  0  0  0  0
    1.5407    1.6960    0.0000 C   0  0  0  0  0  0  0  0  0  0  0  0
```

```
 -1.8361  -0.1770   0.0000 C   0  0  0  0  0  0  0  0  0  0  0  0
 -2.8729   0.7927   0.0001 C   0  0  0  0  0  0  0  0  0  0  0  0
 -4.1753   0.3847   0.0001 C   0  0  0  0  0  0  0  0  0  0  0  0
 -4.5019  -1.0016  -0.0000 C   0  0  0  0  0  0  0  0  0  0  0  0
 -3.4834  -1.9644  -0.0001 C   0  0  0  0  0  0  0  0  0  0  0  0
 -2.1784  -1.5611  -0.0001 C   0  0  0  0  0  0  0  0  0  0  0  0
 -5.7583  -1.3834  -0.0000 O   0  0  0  0  0  0  0  0  0  0  0  0
 -5.2622   1.1844   0.0001 O   0  0  0  0  0  0  0  0  0  0  0  0
 -0.6375   2.5609  -0.0000 O   0  0  0  0  0  0  0  0  0  0  0  0
  1.9082   2.8749  -0.0002 O   0  0  0  0  0  0  0  0  0  0  0  0
  4.5634  -2.9225  -0.0000 O   0  0  0  0  0  0  0  0  0  0  0  0
  4.3911   1.7959  -0.0001 O   0  0  0  0  0  0  0  0  0  0  0  0
  0.0092   3.3031  -0.0000 H   0  0  0  0  0  0  0  0  0  0  0  0
 -1.3912  -2.2971  -0.0001 H   0  0  0  0  0  0  0  0  0  0  0  0
 -3.7615  -3.0082  -0.0002 H   0  0  0  0  0  0  0  0  0  0  0  0
 -6.3508  -0.6164   0.0001 H   0  0  0  0  0  0  0  0  0  0  0  0
 -5.0428   2.1207   0.0006 H   0  0  0  0  0  0  0  0  0  0  0  0
 -2.6475   1.8463   0.0002 H   0  0  0  0  0  0  0  0  0  0  0  0
  2.0254  -2.8615   0.0001 H   0  0  0  0  0  0  0  0  0  0  0  0
  5.5150  -2.7809  -0.0001 H   0  0  0  0  0  0  0  0  0  0  0  0
  5.6116  -0.4552  -0.0001 H   0  0  0  0  0  0  0  0  0  0  0  0
  3.7428   2.5229  -0.0000 H   0  0  0  0  0  0  0  0  0  0  0  0
1  2  4  0  0  0  0
1  6  4  0  0  0  0
1 22  1  0  0  0  0
2  3  1  0  0  0  0
2 10  4  0  0  0  0
3  4  1  0  0  0  0
3  7  2  0  0  0  0
4  5  4  0  0  0  0
4 29  1  0  0  0  0
5  6  4  0  0  0  0
5 21  1  0  0  0  0
6 31  1  0  0  0  0
```

```
  7  8  4  0  0  0  0
  8  9  4  0  0  0  0
  8 11  1  0  0  0  0
  9 10  4  0  0  0  0
  9 19  1  0  0  0  0
 10 20  1  0  0  0  0
 11 12  4  0  0  0  0
 11 16  4  0  0  0  0
 12 13  4  0  0  0  0
 12 28  1  0  0  0  0
 13 14  4  0  0  0  0
 13 18  1  0  0  0  0
 14 15  4  0  0  0  0
 14 17  1  0  0  0  0
 15 16  4  0  0  0  0
 15 25  1  0  0  0  0
 16 24  1  0  0  0  0
 17 26  1  0  0  0  0
 18 27  1  0  0  0  0
 19 23  1  0  0  0  0
 21 30  1  0  0  0  0
 22 32  1  0  0  0  0
M  END
$$$$
 VitC


 20 20  0  0  0  0  0  0  0  0  0 V2000
   -0.1870   -0.3021    0.6653 C   0  0  0  0  0  0  0  0  0  0  0  0
    0.6376    0.9137    0.3486 C   0  0  0  0  0  0  0  0  0  0  0  0
    1.8271    0.5276   -0.1058 C   0  0  0  0  0  0  0  0  0  0  0  0
    1.8759   -0.9358   -0.0861 C   0  0  0  0  0  0  0  0  0  0  0  0
    0.6968   -1.4026    0.3833 O   0  0  0  0  0  0  0  0  0  0  0  0
    0.1505    2.1479    0.5710 O   0  0  0  0  0  0  0  0  0  0  0  0
```

2.8768	1.2804	-0.4913	O	0	0	0	0	0	0	0	0	0	0	0	0
2.8063	-1.6154	-0.4151	O	0	0	0	0	0	0	0	0	0	0	0	0
-1.4489	-0.4804	-0.1654	C	0	0	0	0	0	0	0	0	0	0	0	0
-2.3698	0.7290	-0.1183	C	0	0	0	0	0	0	0	0	0	0	0	0
-2.1103	-1.6053	0.3688	O	0	0	0	0	0	0	0	0	0	0	0	0
-3.5570	0.3093	-0.7872	O	0	0	0	0	0	0	0	0	0	0	0	0
-0.4510	-0.3482	1.7229	H	0	0	0	0	0	0	0	0	0	0	0	0
0.8248	2.8000	0.3538	H	0	0	0	0	0	0	0	0	0	0	0	0
3.5924	0.6773	-0.7302	H	0	0	0	0	0	0	0	0	0	0	0	0
-1.1505	-0.6631	-1.2038	H	0	0	0	0	0	0	0	0	0	0	0	0
-1.9179	1.5810	-0.6239	H	0	0	0	0	0	0	0	0	0	0	0	0
-2.5835	0.9892	0.9221	H	0	0	0	0	0	0	0	0	0	0	0	0
-2.9833	-1.6288	-0.0354	H	0	0	0	0	0	0	0	0	0	0	0	0
-4.2439	0.9660	-0.6715	H	0	0	0	0	0	0	0	0	0	0	0	0

1	2	1	0	0	0	0
1	5	1	0	0	0	0
1	9	1	0	0	0	0
1	13	1	0	0	0	0
2	3	2	0	0	0	0
2	6	1	0	0	0	0
3	4	1	0	0	0	0
3	7	1	0	0	0	0
4	5	1	0	0	0	0
4	8	1	0	0	0	0
6	14	1	0	0	0	0
7	15	1	0	0	0	0
9	10	1	0	0	0	0
9	11	1	0	0	0	0
9	16	1	0	0	0	0
10	12	1	0	0	0	0
10	17	1	0	0	0	0
10	18	1	0	0	0	0
11	19	1	0	0	0	0
12	20	1	0	0	0	0

```
M  END
$$$$
 VitC_A


 19 19  0  0  1  0  0  0  0  0999 V2000
   -0.2004   -0.1529    0.6766 C   0  0  3  0  0  0  0  0  0  0  0  0
    0.6317    1.0833    0.3045 C   0  0  0  0  0  0  0  0  0  0  0  0
    1.8537    0.5599   -0.0959 C   0  0  0  0  0  0  0  0  0  0  0  0
    1.8645   -0.8468   -0.0056 C   0  0  0  0  0  0  0  0  0  0  0  0
    0.6363   -1.3027    0.4703 O   0  0  0  0  0  0  0  0  0  0  0  0
    0.1651    2.2318    0.3879 O   0  5  0  0  0  0  0  0  0  0  0  0
    2.9716    1.2353   -0.5428 O   0  0  0  0  0  0  0  0  0  0  0  0
    2.7608   -1.6305   -0.2786 O   0  0  0  0  0  0  0  0  0  0  0  0
   -1.4275   -0.3281   -0.1979 C   0  0  3  0  0  0  0  0  0  0  0  0
   -2.5177    0.6803    0.0906 C   0  0  0  0  0  0  0  0  0  0  0  0
   -2.0073   -1.6180    0.0397 O   0  0  0  0  0  0  0  0  0  0  0  0
   -3.6796    0.3784   -0.6772 O   0  0  0  0  0  0  0  0  0  0  0  0
   -0.4881   -0.1242    1.7297 H   0  0  0  0  0  0  0  0  0  0  0  0
    3.6119    0.5422   -0.7355 H   0  0  0  0  0  0  0  0  0  0  0  0
   -1.1231   -0.2578   -1.2474 H   0  0  0  0  0  0  0  0  0  0  0  0
   -2.1769    1.6781   -0.1665 H   0  0  0  0  0  0  0  0  0  0  0  0
   -2.7527    0.6483    1.1598 H   0  0  0  0  0  0  0  0  0  0  0  0
   -1.2755   -2.2430    0.0354 H   0  0  0  0  0  0  0  0  0  0  0  0
   -3.7962   -0.5730   -0.6036 H   0  0  0  0  0  0  0  0  0  0  0  0
  1 13  1  0  0  0  0
  1  9  1  0  0  0  0
  1  2  1  0  0  0  0
  1  5  1  0  0  0  0
  2  3  2  0  0  0  0
  2  6  1  0  0  0  0
  3  4  1  0  0  0  0
  3  7  1  0  0  0  0
  4  5  1  0  0  0  0
```

```
  4  8  2  0  0  0  0
  7 14  1  0  0  0  0
  9 15  1  0  0  0  0
  9 10  1  0  0  0  0
  9 11  1  0  0  0  0
 10 16  1  0  0  0  0
 10 17  1  0  0  0  0
 10 12  1  0  0  0  0
 11 18  1  0  0  0  0
 12 19  1  0  0  0  0
M  CHG  1   6  -1
M  END
$$$$
 VitC_NR


 19 19  0  0  0  0  0  0  0  0  0 V2000
   -0.1915   -0.2540    0.6592 C   0  0  0  0  0  0  0  0  0  0  0  0
    0.5858    1.0316    0.3610 C   0  0  0  0  0  0  0  0  0  0  0  0
    1.8636    0.5800   -0.0936 C   0  0  0  0  0  0  0  0  0  0  0  0
    1.9019   -0.8826   -0.0628 C   0  0  0  0  0  0  0  0  0  0  0  0
    0.7230   -1.3436    0.3808 O   0  0  0  0  0  0  0  0  0  0  0  0
    0.1696    2.1641    0.5065 O   0  0  0  0  0  0  0  0  0  0  0  0
    2.8953    1.2885   -0.4764 O   0  0  0  0  0  0  0  0  0  0  0  0
    2.8476   -1.5497   -0.3808 O   0  0  0  0  0  0  0  0  0  0  0  0
   -1.4431   -0.4401   -0.1793 C   0  0  0  0  0  0  0  0  0  0  0  0
   -2.4131    0.7246   -0.0455 C   0  0  0  0  0  0  0  0  0  0  0  0
   -2.0493   -1.6264    0.2811 O   0  0  0  0  0  0  0  0  0  0  0  0
   -3.5824    0.2951   -0.7400 O   0  0  0  0  0  0  0  0  0  0  0  0
   -0.4527   -0.3087    1.7157 H   0  0  0  0  0  0  0  0  0  0  0  0
    3.6083    0.6709   -0.7102 H   0  0  0  0  0  0  0  0  0  0  0  0
   -1.1512   -0.5426   -1.2314 H   0  0  0  0  0  0  0  0  0  0  0  0
   -2.0023    1.6306   -0.4866 H   0  0  0  0  0  0  0  0  0  0  0  0
   -2.6323    0.8991    1.0111 H   0  0  0  0  0  0  0  0  0  0  0  0
```

```
  -2.9303   -1.6512   -0.1064 H   0  0  0  0  0  0  0  0  0  0  0  0
  -4.2924    0.9209   -0.5955 H   0  0  0  0  0  0  0  0  0  0  0  0
  1  2  1  0  0  0  0
  1  5  1  0  0  0  0
  1  9  1  0  0  0  0
  1 13  1  0  0  0  0
  2  3  1  0  0  0  0
  2  6  1  0  0  0  0
  3  4  1  0  0  0  0
  3  7  1  0  0  0  0
  4  5  1  0  0  0  0
  4  8  1  0  0  0  0
  7 14  1  0  0  0  0
  9 10  1  0  0  0  0
  9 11  1  0  0  0  0
  9 15  1  0  0  0  0
 10 12  1  0  0  0  0
 10 16  1  0  0  0  0
 10 17  1  0  0  0  0
 11 18  1  0  0  0  0
 12 19  1  0  0  0  0
M  END
$$$$
 VitC_PR


 20 20  0  0  0  0  0  0  0  0  0 V2000
  -0.1984   -0.2366    0.6604 C   0  0  0  0  0  0  0  0  0  0  0  0
   0.6267    0.9676    0.3313 C   0  0  0  0  0  0  0  0  0  0  0  0
   1.8751    0.5211   -0.1190 C   0  0  0  0  0  0  0  0  0  0  0  0
   1.8748   -0.9733   -0.0510 C   0  0  0  0  0  0  0  0  0  0  0  0
   0.6886   -1.3532    0.4231 O   0  0  0  0  0  0  0  0  0  0  0  0
   0.1987    2.1709    0.4814 O   0  0  0  0  0  0  0  0  0  0  0  0
   2.8674    1.2389   -0.5049 O   0  0  0  0  0  0  0  0  0  0  0  0
```

```
  2.8033   -1.6391   -0.3678 O   0  0  0  0  0  0  0  0  0  0  0  0
 -1.4635   -0.4242   -0.1882 C   0  0  0  0  0  0  0  0  0  0  0  0
 -2.4715    0.7095   -0.0281 C   0  0  0  0  0  0  0  0  0  0  0  0
 -1.9954   -1.6258    0.2812 O   0  0  0  0  0  0  0  0  0  0  0  0
 -3.5949    0.2419   -0.7458 O   0  0  0  0  0  0  0  0  0  0  0  0
 -0.4740   -0.2462    1.7178 H   0  0  0  0  0  0  0  0  0  0  0  0
  0.8456    2.8560    0.2412 H   0  0  0  0  0  0  0  0  0  0  0  0
  3.6277    0.6763   -0.7601 H   0  0  0  0  0  0  0  0  0  0  0  0
 -1.1718   -0.5025   -1.2431 H   0  0  0  0  0  0  0  0  0  0  0  0
 -2.0956    1.6435   -0.4506 H   0  0  0  0  0  0  0  0  0  0  0  0
 -2.7036    0.8473    1.0322 H   0  0  0  0  0  0  0  0  0  0  0  0
 -2.8699   -1.7313   -0.1135 H   0  0  0  0  0  0  0  0  0  0  0  0
 -4.3583    0.8025   -0.5944 H   0  0  0  0  0  0  0  0  0  0  0  0
  1  2  1  0  0  0  0
  1  5  1  0  0  0  0
  1  9  1  0  0  0  0
  1 13  1  0  0  0  0
  2  3  2  0  0  0  0
  2  6  1  0  0  0  0
  3  4  1  0  0  0  0
  3  7  1  0  0  0  0
  4  5  1  0  0  0  0
  4  8  1  0  0  0  0
  6 14  1  0  0  0  0
  7 15  1  0  0  0  0
  9 10  1  0  0  0  0
  9 11  1  0  0  0  0
  9 16  1  0  0  0  0
 10 12  1  0  0  0  0
 10 17  1  0  0  0  0
 10 18  1  0  0  0  0
 11 19  1  0  0  0  0
 12 20  1  0  0  0  0
M  END
```

$$$$
